# Tuning the Properties of 5‐Azido and 5‐Nitramino‐tetrazoles by Diverse Functionalization – General Concepts for Future Energetic Materials

**DOI:** 10.1002/chem.202200772

**Published:** 2022-05-19

**Authors:** Maximilian Benz, Thomas M. Klapötke, Tobias Lenz, Jörg Stierstorfer

**Affiliations:** ^1^ Department of Chemistry Ludwig Maximilian University of Munich Butenandtstr. 5–13 D-81337 Munich Germany

**Keywords:** azides, energetic materials, NMR spectroscopy, tetrazoles, X-ray diffraction

## Abstract

5‐Azido and 5‐nitraminotetrazole backbones are established heterocyclic motifs in the research field of energetic materials synthesis. Despite the high energy content of the compounds, the problem with many derivatives is that their sensitivities are far too high. Functionalization of one of the ring nitrogen atoms is the aim of this study to adjust the sensitivity by inserting nitratoethyl, azidoethyl and methyl groups. In this context, derivatives of 2‐(2‐azidoethyl)‐5‐nitraminotetrazoles (**2**, **2** **a**–**2** **d**), as well as 1‐nitrato and 1‐azidoethyl substituted 5‐azidotetrazole (**7** and **10**) and the methylation products of 5‐azidotetrazole (5‐azido‐1‐methyl‐tetrazole, **11** and 5‐azido‐2‐methyl‐tetrazole, **12**) were prepared. The obtained nitrogen‐rich compounds were extensively characterized through multinuclear NMR spectroscopy and IR spectroscopy. The structural confinement was checked by X‐ray diffraction experiments. The pure samples (verified by elemental analysis) were investigated regarding their behavior toward friction, impact (BAM methods) and electrostatic discharge as well as heating (DTA and DSC). For all metal‐free compounds the detonation properties were computed with the EXPLO5 code using their density and heat of formation, calculated based on CBS‐4 M level of theory.

## Graphical abstract

**Derivatization of 5‐azido** and 5‐nitraminotetrazole with an azidoethyl, nitratoethyl or methyl group gives access to less acidic high explosives with versatile properties. Salt formation of the nitraminotetrazoles with nitrogen‐rich and metal cations was performed to achieve less acidity, lower vapor pressure and higher thermal stability. The modification of the nitramino and azidotetrazole backbones show and nice way to tune the compounds’ properties and are valuable for the design of future energetic materials.

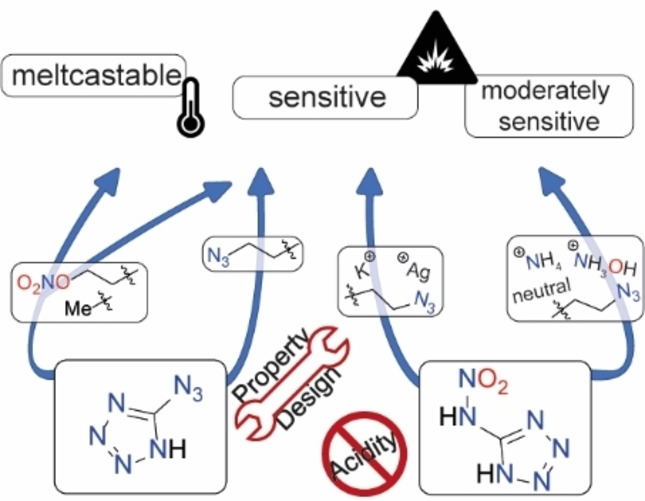

## Introduction

**Figure 1:**
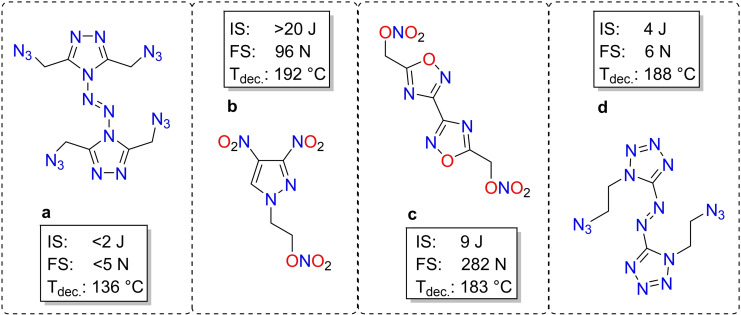
Representatives of energetic N‐alkyl substituted azoles. 3,3’,5,5’‐Tetra(azidomethyl)‐4,4’‐azo‐1,2,4‐triazole (a), 1(2‐nitratoethyl)‐3,4‐dinitropyrazole (b), bis(1,2,4‐oxadiazole)bis(methylene) dinitrate (c) and 1,1’‐bis(2‐azidoethyl)‐azotetrazole (d).

The application of energetic materials is spread over various fields and thus a material must fulfill suitable criteria.^[1]^ Explosives are divided into three main categories. A) Sensitive explosives, such as sensitizers, igniters or detonants; B) less sensitive but powerful explosives, secondary explosives; and C) insensitive, tertiary explosives. Many of the materials used were developed before or at the beginning of the 20th century, with only a little focus on sustainability or environmental impact.^[2]^ The common primary explosives are often heavy metal based, as there are lead azide or styphnate. Their use is found to contaminate training grounds and application sites.^[3]^ The secondary explosive trinitrotoluene, which is widely used due to its advantageous melt castable properties, shows to have disadvantageous environmental impact and is a possible carcinogenic, as stated by the U.S. Environmental Protection Agency.^[4a]^ The urgent need for sustainable and environmentally friendly replacements poses major challenges for the energetic materials community.^[4b]^ A state‐of‐the‐art synthetic approach consists of derivatization of known oxygen‐ and nitrogen‐rich azoles in combination with short alkyl, azidoalkyl or nitratoalkyl chains. Sabatini et al. found that bis‐5‐(nitratomethyl)bis(1,2,4‐oxadiazole) (c) is suitable to replace trinitrotoluene while showing significantly higher performance data.^[5]^ Furthermore furazanes^[6]^, 1,2,3‐triazoles^[7]^, 1,2,4‐triazoles^[8]^, tetrazoles^[9]^ and pyrazoles^[10]^ were functionalized by energetic azido or nitratoalkyl side chains (Figure 1).

**Figure 2:**
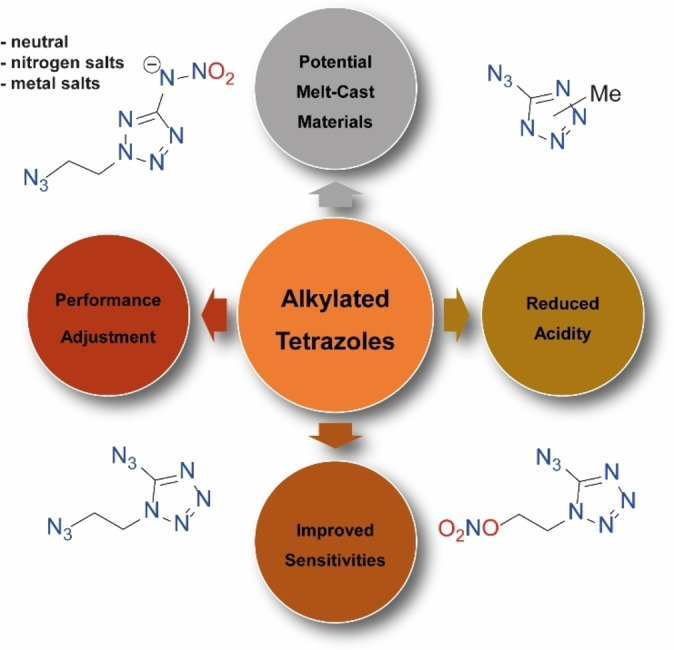
Derivatization of 5‐azido‐ and 5‐nitraminotetrazole reveals new applications.

In the search for ever higher nitrogen and energy contents, the energetic compounds, 5‐nitramino‐^[11]^ and 5‐azidotetrazole^[12]^, arouse attention. Both molecules are intensively studied as free acids^[13]^ or salinized,^[14]^ and are promising building blocks due to their straight forward accessibility. Here we report different approaches to reduce the acidity of those high energy and nitrogen‐rich tetrazole building blocks. Insights into the changes of the sensitivity are gained. For the 5‐nitraminotetrazole replacement of the acidic tetrazole proton by an azidoethyl function led to a monobasic acid (**2**) that was further salinized (**2** **a–d**) to reduce the acidity, lower the vapor pressure and increase the thermal stability. Also melt‐castable primary explosives would be of great interest. Therefore, the acidic proton of 5‐azidotetrazole was exchanged by an azidoethyl (**7**), nitratoethyl (**10**) or methyl (**11**, **12**) functionality. Functionalization reveals compounds that are suitable for various applications (Figure 2).

## Synthesis

**Scheme 1:**
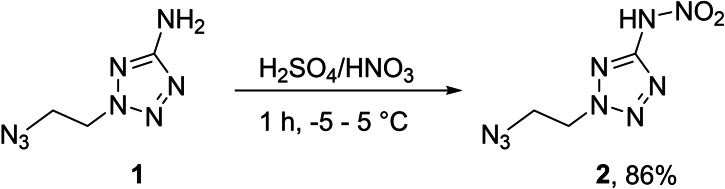
Nitration of **1** to form the nitramino derivative **2** using mixed acid at low temperatures.

With the synthetic protocol for 2‐(2‐azidoethyl)‐5‐aminotetrazole (**1**) in hand, which was recently published by our group^[9a]^, we were investigating suitable conditions to perform the nitration of the amino function to the respective nitraminotetrazole **2**. We found a pre‐cooled mixture of sulfuric acid and fuming nitric acid (ratio 8 : 3) to work best and this yields pure **2** without further purification in 86 % yield (Scheme 1).

**Scheme 2:**
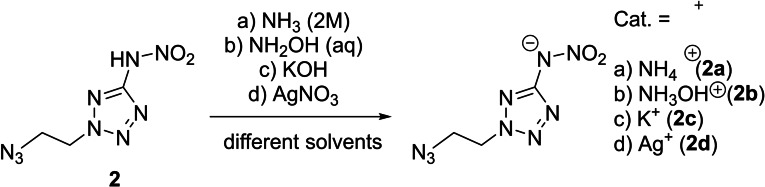
Preparation of ionic derivatives **2** **a**–**2** **d** starting from the free nitramino derivative **2** through reaction with the respective cation containing reagents.

The nitrogen‐rich salts **2** **a** and **2** **b** as well as the metal salts **2** **c** and **2** **d** were obtained by simple acid‐base reactions of the free nitramine **2** and the respective base, or in the case of **2** **d** with AgNO_3_ solution (Scheme 2). All compounds were obtained elemental analysis pure and could be analyzed without further purification. In attempting to prepare the hydrazinium salt of **2**, pure **1** was isolated. As has already occurred for other aromatic nitramines^[9b]^, the reaction with aqueous hydrazine leads to a reduction of the nitramine function to the amine.

**Scheme 3:**
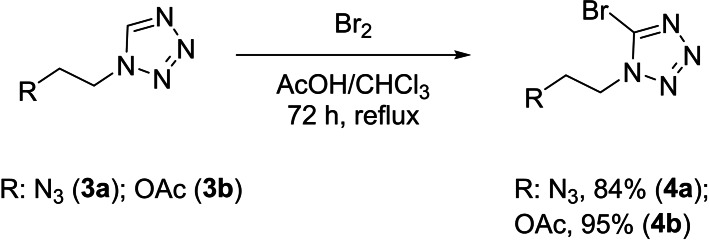
Bromination of 1‐functionalized 5*H*‐tetrazoles.

To achieve functionalized 5‐azidotetrazoles classic bromination of 5*H*‐tetrazoles was performed for three days at reflux (Scheme 3). The 1‐(2‐azidoethyl)‐tetrazole (**3** **a**) was reacted to 1‐(2‐azidoethyl)‐5‐bromotetrazole (**4** **a**) in very good yields. The bromination of 1‐(2‐acetoxyethyl)‐tetrazole (**3** **b**) was performed similar to the literature,^[15]^ and 5‐bromo‐1‐(2‐acetoxyethyl)‐tetrazole (**4** **b**) was yielded in excellent yields (Scheme 3).

**Scheme 4:**
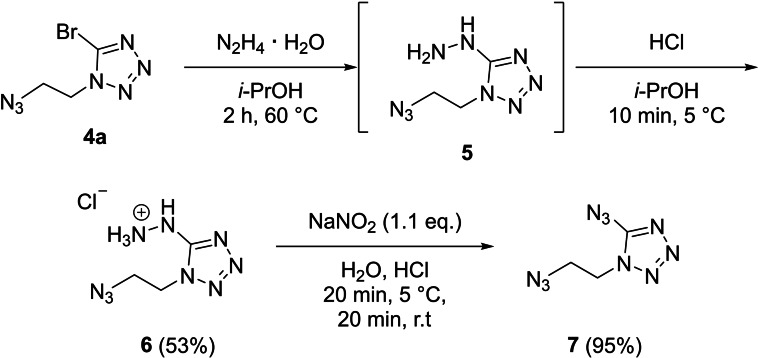
Reaction pathway towards 1‐(2‐azidoethyl)‐5‐azidotetrazole (**7**) by reacting the 5‐position of the tetrazole from bromine to hydrazine to azide.

Substitution at the 5‐bromo position was then performed by hydrazine. The strong nucleophilicity of hydrazine and the low solubility of the resulting hydrazinium bromide in isopropanol makes bromine‐hydrazine exchange possible, even at room temperature. The reaction was performed either at room temperature for three days or at elevated temperature for shorter periods of time. Compound **4** **a** can be reacted at 60 °C for 2 h to synthesize 1‐(2‐azidoethyl)‐5‐hydrazineyltetrazole (**5**). However slow decomposition at room temperature complicated the purification and therefore only a crystal structure and ^1^H and ^13^C NMR were measured. To stabilize and purify compound **5** it was precipitated as hydrochloride salt, 1‐(2‐azidoethyl)‐5‐hydraziniumtetrazol chloride (**6**), from a concentrated isopropanol solution. The overall reaction from compound **4** **a** to compound **6** proceeds in acceptable yields. Finally, 1‐(2‐azidoethyl)‐5‐azidotetrazole (**7**) was synthesized through reaction of **6** with sodium nitrite under acidic conditions in excellent yields after column chromatography (Scheme 4).

**Scheme 5:**
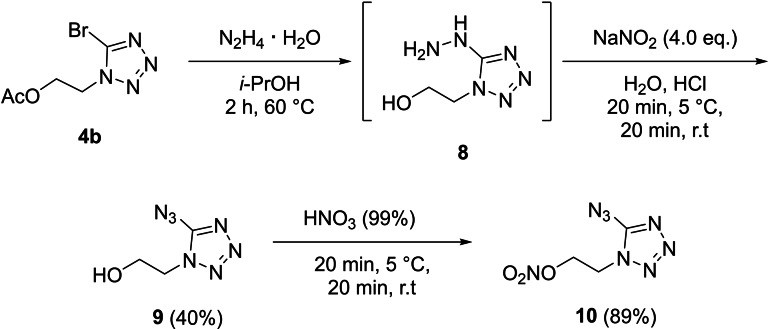
Reaction pathway towards 5‐Azido‐1‐(2‐nitratoethyl)‐tetrazole (**10**) by bromine‐hydrazine exchange followed by reaction with sodium nitrite and the nitration of the hydroxy group to its organic nitrate.

Reaction of compound **4** **b** with hydrazine over three days at ambient temperature lead to 5‐hydrazineyl‐1‐(2‐hydroxyethyl)‐tetrazole (**8**). The acetoxy moiety of **4** **b** reacts with hydrazine to acetohydrazide. Several attempts to purify compound (**8**) failed, only a crystal structure and ^1^H and ^13^C NMR were measured. Also, a crystal structure of the hydrochloride was observed by a similar procedure than for **6** (can be found in the Supporting Information). The crude of **5** was reacted with sodium nitrite under acidic conditions to give 5‐azido‐1‐(2‐hydroxyethyl)‐tetrazole (**9**). After column chromatography the overall yield from **4** **b** to **9** is 40 %. In the last step of the synthesis towards 5‐azido‐1‐(2‐nitratoethyl)‐tetrazole (**10**), compound **9** was nitrated using white fuming nitric acid in very good yields (Scheme 5).

**Scheme 6:**
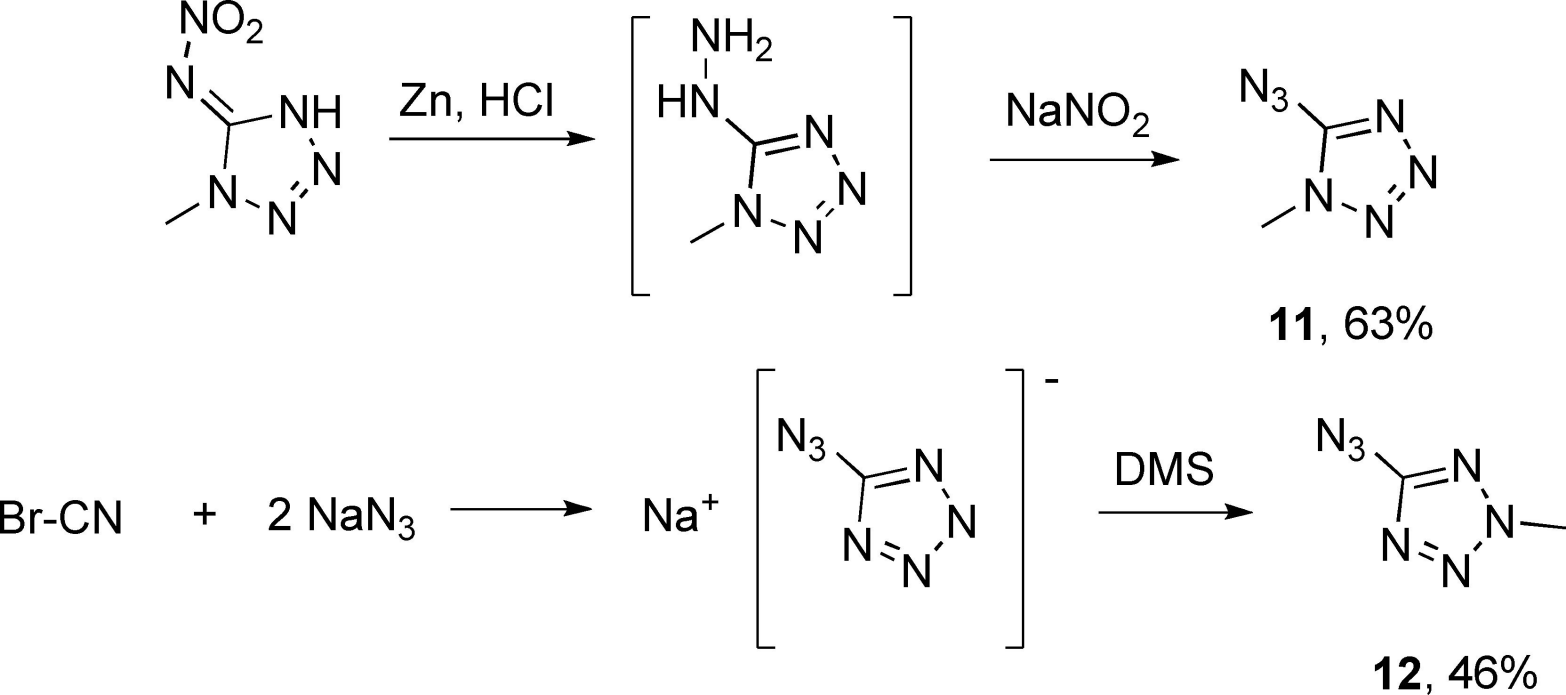
Pathway for selective synthesis of 5‐azido‐1‐methyltetrazole (**11**) through diazotization of 5‐hydrazino‐1‐methyltetrazole and 5‐azido‐2‐methyltetrazole (**12**) through methylation of 5‐azidotetrazole.

For the synthesis of the two isomers of 5‐azido‐methyltetrazole, various synthetic pathways had to be pursued as depicted in Scheme 6. Methylation of in situ generated 5‐azidotetrazole with dimethyl sulfate produces only the 5‐azido‐2‐methyltetrazole (**12**) isomer due to the two‐position directing effect of the azido group. Therefore, an alternative route toward the 5‐azido‐1‐methyltetrazole (**11**) had to be established starting with a 1‐methyl substituted tetrazole derivative and subsequent generation of the azide function at position 5.

Thus, we started with 1‐methyl‐5‐nitrimino‐4*H*‐tetrazole,^[16]^ and were able to reduce the nitrimino function with zinc dust under acidic conditions to the respective 5‐hydrazino‐1‐methyltetrazole, which was directly converted to 5‐azido‐1‐methyltetrazole (**11**) through diazotization using one equivalent of sodium nitrite.

## Crystal Structures

Crystals suitable for X‐ray diffraction experiments could be obtained by recrystallization from different common solvents for **2** **a**, **2** **b**, **2** **d** and **10–12**. Deposition Numbers 2157201 (for **2** **a**), 2157198 (for **2** **b**), 2157203 (for **2** **d**), 2157204 (for **5**), 2157196 (for **6**), 2157199 (for **7**), 2157197 (for **8** ⋅ HCl), 2157202 (for **9**), 2157200 (for **10**), 707542 (for **11**) and 707543 (for **12**) contain(s) the supplementary crystallographic data for this paper. These data are provided free of charge by the joint Cambridge Crystallographic Data Centre and Fachinformationszentrum Karlsruhe Access Structures service. Crystalline diazido derivative **7** could be generated and picked by cooling of a saturated solution of **7** in Et_2_O at −30 °C since it is a liquid at room temperature. Additional information about the depicted crystal structures and the measuring and computation methods as well as further crystal structures for various precursor compounds (**5**, **6**, **8**, **9**) can be found in the Supporting Information.

Ammonium and hydroxylammonium salts **2** **a** and **2** **b** crystallize both in the triclinic space group *P*−1. As expected, the hydroxylammonium derivative has a slightly higher room temperature density (1.624 g cm^−3^ for **2** **b**) then the respective ammonium salt (1.575 g cm^−3^ for **2** **a**). The densities are in a range that was expected, when comparing them with related crystal structure densities. As shown in detail in previous studies[^9a^, ^9b^, ^9d^] the densities of *N*‐azidoethyl substituted tetrazoles are always well below those of *N*‐nitratoethyl substituted ones. Compared with the room temperature densities of the hydroxylammonium salts of 1‐and 2‐nitratoethyl‐5‐nitraminotetrazole (density for both is 1.720 g cm^−3^)^[9e]^, the density of **2** **b** is about 0.1 g cm^−3^ lower, which was to be expected due to the substitution at position two.

**Figure 3:**
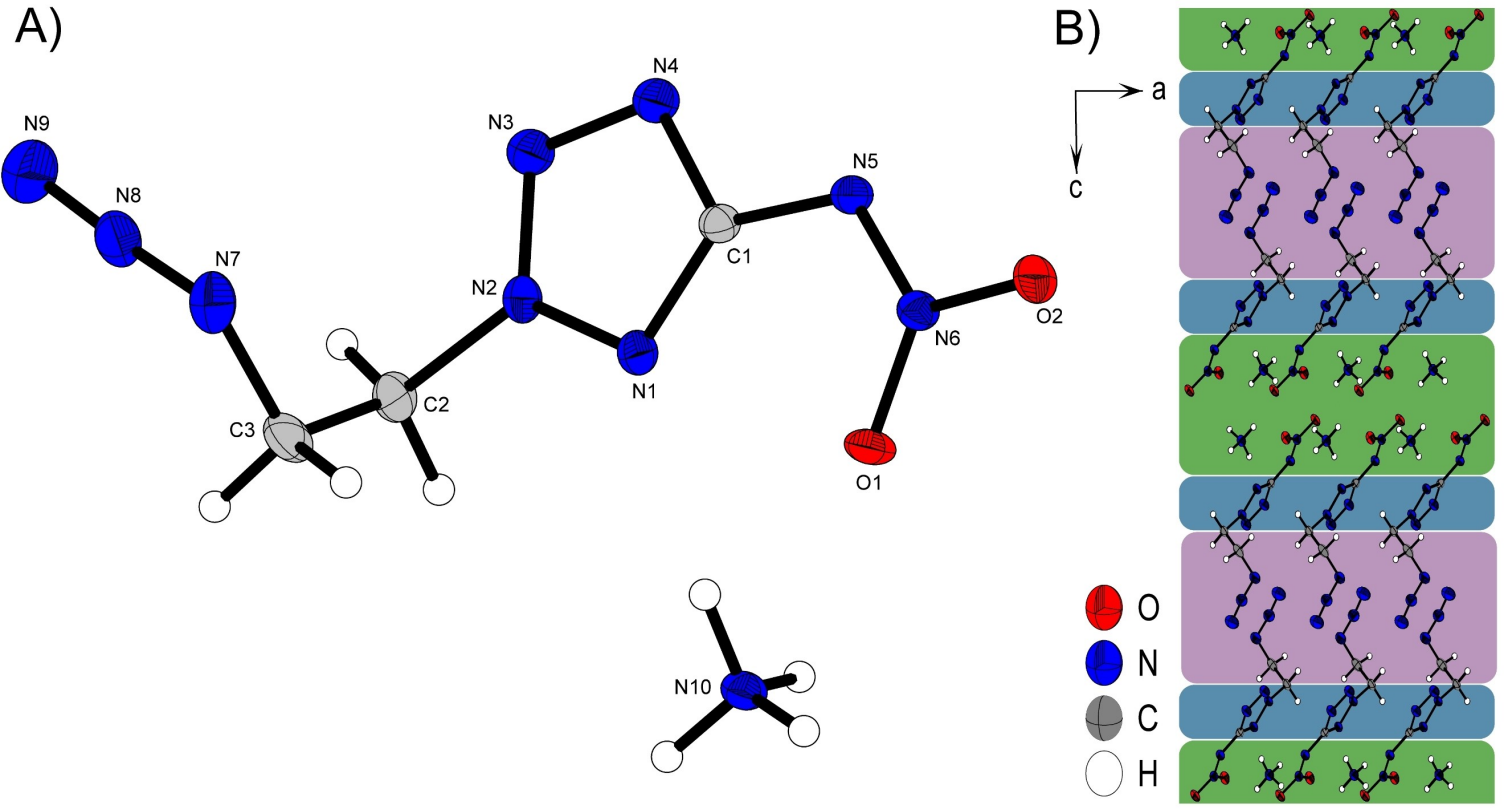
A) Molecular unit of **2** **a** with thermal ellipsoids drawn at the 50 % probability level. Selected bond distances (Å) and angles [°]: C1‐N5 1.382(2), C2‐N2 1.467(2), N7‐N8 1.241(2), N8‐N9 1.128(2), N10‐H10A⋅⋅⋅O1 2.10(2), N10‐H10B⋅⋅⋅O2 2.02(2), N10‐H10C⋅⋅⋅N1 2.12(2), N10‐H10D⋅⋅⋅N5 2.05(2), N7‐N8‐N9 173.6(2), N7‐C3‐C2‐N2 62.08(2), O1‐N6‐N5‐C1 1.7(2); B) Layer strcutre of **2** **a** with view along the *b* axis. Green, blue and red regions are labeled based on their different components for intermolecular bond formation.

**Figure 4:**
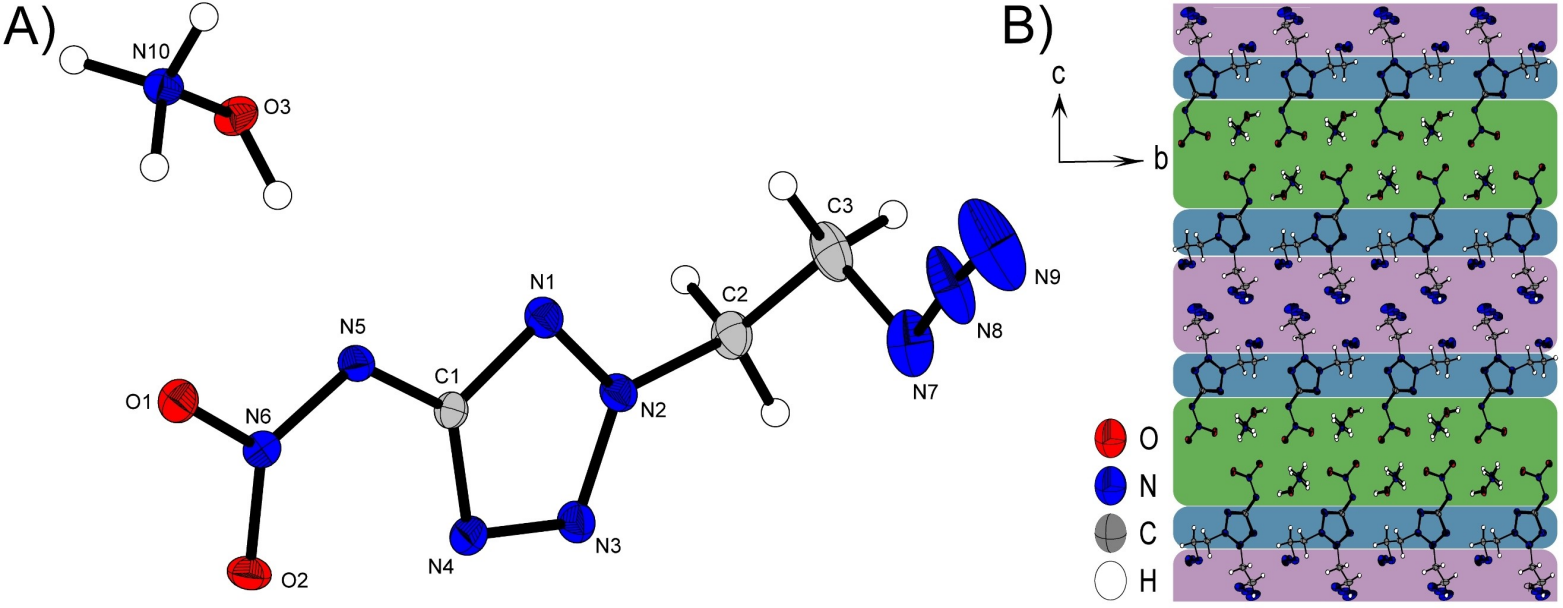
A) Asymmetric unit of **2** **b** with thermal ellipsoids drawn at the 50 % probability level. Selected bond distances (Å) and angles [°]: C1‐N5 1.392(3), C2‐N2 1.472(3), N7‐N8 1.256(3), N8‐N9 1.128(4), N10‐H10A⋅⋅⋅O4 2.22(3), N10‐H10B⋅⋅⋅O2 2.08(3), N10‐H10C⋅⋅⋅N11 2.13(3), N10‐H10C⋅⋅⋅O5 2.15(3), O3‐H3⋅⋅⋅N5 1.80(4), N7‐N8‐N9 173.5(4), N7‐C3‐C2‐N2 69.9(3), O1‐N6‐N5‐C1 0.2(2); B) Layer structure of **2** **b** with view along the *a* axis. Green, blue and red regions are labeled based on their different components for intermolecular bond formation.

The three‐dimensional configuration of the two salts **2** **a** and **2** **b** behave very similarly and can be schematically divided into three different parts depicted in Figure 3 B) Figure 4 B). The interactions in the red areas are exclusively weak van der Waals interactions between the C‐H protons of the ethyl groups and the azide functions. The shortest ones can be found in **2** **b** (C2‐H2 A*⋅⋅⋅*N19 2.65 Å) and are therefore negligible for stability discussion due to their weak character. In the blue region are mainly the tetrazole rings, which also loosely interact with each other through π–π‐interactions. But mainly this layer forms both, linking and buffer layer between non‐polar, weakly interacting azidoethyl sites and the strongly stabilizing, polar sites consisting of the respective cations and nitramine functions, highlighted in green. In this region of the two crystal structures of **2** **a** and **2** **b**, dominated by strong polar interactions, the overall strongest intermolecular, ionic interactions are found. These are formed through all protons of the cations with the respective nitrogen and oxygen atoms of the nitramine function (O1, O2 and N6) as well as with N1 of the tetrazole moiety. The average bond length *d*(D−H⋅⋅⋅A) is 2.06 Å with O3‐H3⋅⋅⋅N5 1.80(4) Å being the shortest hydrogen bridge and therefore clearly below the van der Waals radius for a strong interaction.

**Figure 5:**
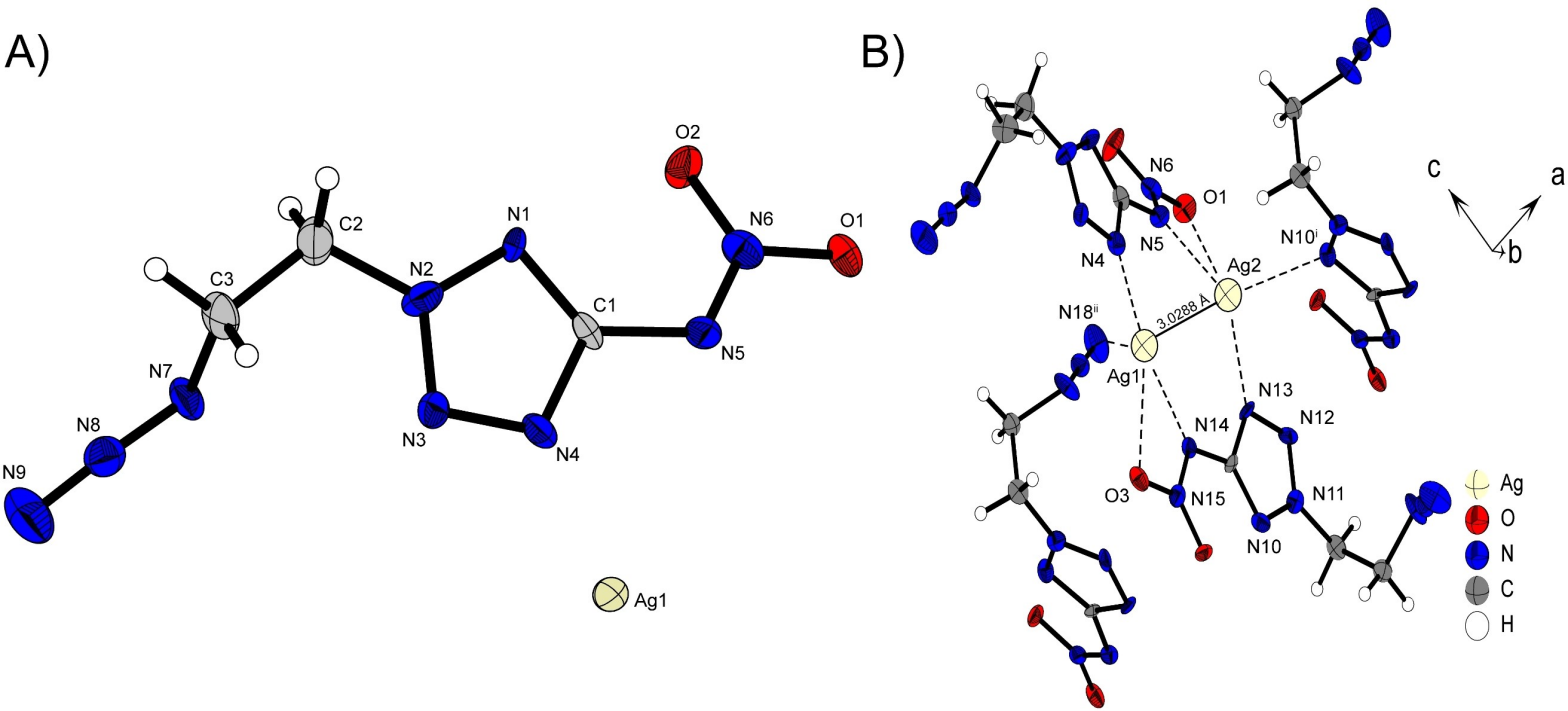
A) Asymmetric unit of **2** **d** with thermal ellipsoids drawn at the 50 % probability level. Selected bond distances (Å) and angles [°]: Ag1‐Ag2 3.0288(11), Ag1‐N14 2.126(7), Ag1‐O3 2.877(6), Ag1‐N4 2.137(7), Ag1‐N18^ii^ 3.154(10), Ag2‐N13 2.216(7), Ag2‐N5 2.201(7), Ag2‐O1 2.822(7), Ag2‐N10^i^ 2.489(7), N14‐Ag1‐N4 167.3(3), N13‐Ag2‐N5 144.8(3), N15‐N14‐C4‐N10 8.4(1), N6‐N5‐C1‐N1 29.3(1); B) Representation of the argenophilic interaction between Ag1 and Ag2 with anionic moieties involved in the stabilization; Symmetry codes: (i) 0.5+x, 0.5−y, 1−z, (ii) 1−x, 1−y, 1−z.

Compound **2** **d** crystalizes in the orthorhombic space group *Pbca* with a density of 2.362 g cm^−3^ at 298 K and 16 molecular moieties per unit cell. The structure is mainly characterized by the formation of a close silver‐silver contact. The neighboring anions arrange themselves around this structural motif in such manner that the formed argentophilic interaction can be interactively stabilized in the best possible way as depicted in Figure 5 B). Argentophilic interactions are van der Waals contract below a bond distance of 3.44 Å and can be observed in several Ag containing structures.^[17]^ Compared to the shortest ever measured Ag−Ag contact (2.7599(3) Å)^[18]^ the silver‐silver distance is with a length of 3.0288(11) Å clearly above, but can still be considered as argentophilic interaction.^[19]^ The Ag−Ag building block is stabilized by four anionic moieties forming several close contacts. These interactions originate from one nitrogen and one oxygen of the nitramine function, the tetrazole nitrogen atoms N1 and N4 as well as from the γ‐nitrogen of the azide functionality. These electron donating interactions of the complexing N and O atoms are observed in the range of 2.126(7) Å for Ag1‐N14 to 3.154(10) Å for Ag1‐N18^ii^. In order to optimize the assembly around the silver dimer, some unusual arrangements occur within the anion. For example, one nitramino group is clearly twisted out of the plane formed by the tetrazole (N6‐N5‐C1‐N1 29.3(1)°). However, this rotation improves the complexation of the silver. Nevertheless, this arrangement is unusual for 5‐nitraminotetrazoles, which normally bear 5‐substituted nitramino groups planar with the tetrazole ring.

**Figure 6:**
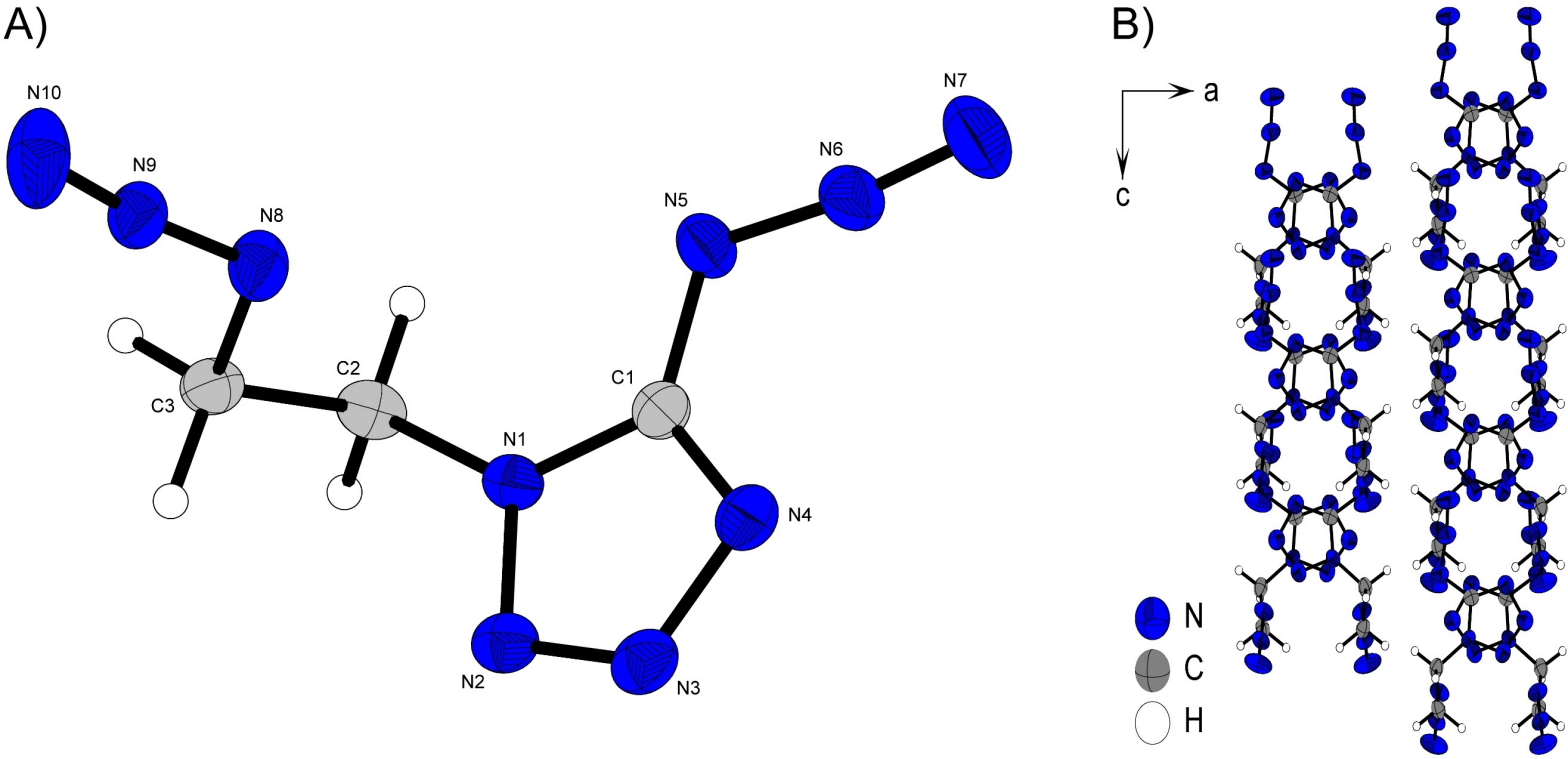
A) Molecular unit of **7** with thermal ellipsoids drawn at the 50 % probability level. Selected bond distances (Å) and angles [°]: C1‐N5 1.386(4), C2‐N1 1.458(4), N5‐N6 1.251(4), N6‐N7 1.114(4), N8‐N9 1.221(4), 1.127(4), N5‐N6‐N7 171.4(3), N8‐N9‐N10 172.9(3), N9‐N8‐C3‐C2 171.1(3); B) Layer structure of **9** with view along the *b* axis.

**Figure 7:**
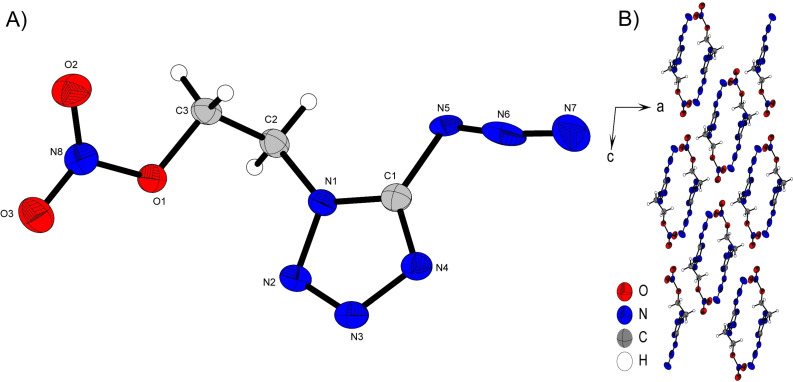
A) Molecular unit of **10** with thermal ellipsoids drawn at the 50 % probability level. Selected bond distances (Å) and angles [°]: C1‐N5 1.463(4), C2‐N1 1.458(4), N5‐N6 1.074(4), N6‐N7 1.207(4), C3‐O1 1.449(4), O1‐N8 1.388(3), N5‐N6‐N7 171.5(3), N1‐C2‐C3 112.6(2), N6‐N5‐C1‐N1 1.7(3); B) Layer structure of **10** with view along the *b* axis.

Ethyl substituted 5‐azidotetrazole derivatives crystallize in the orthorhombic space group *Fdd*2 (for **7**) and in the monoclinic space group *P*2_1_/*n* (for **10**). The molecular moieties are depicted in Figure 6 and Figure 7. The room temperature density of nitratoethyl compound **10** is clearly superior to that of the azidoethyl derivative (1.519 g cm^−3^ for **7**, 1.638 g cm^−3^ for **10**). This trend has also been observed in previous studies^[20]^, since the nitratoethyl unit offers more possibilities for intermolecular interactions on the one hand and contains more and heavier atoms on the other, which also arrange themselves better in space. Since there are no good opportunities for intermolecular interactions for the present nonpolar residues, the three‐dimensional structure of **7** is mainly based on the fact that the azido and azidoethyl substituents avoid each other with a as large spatial distance as possible in order to counteract possible destabilizing interaction (Figure 6 B)).

The three‐dimensional pattern of **10** in Figure 7 B) shows the assembly of two molecular units resulting in a linear chain structure of those pairs along *b*. Schematically, a molecular unit of **10** consists of two molecular planes that diverge from each other at an angle of about 112°. One plane is formed by the azidotetrazole backbone and the second by the ethyl group, in the linear direction of which the nitrate ester is connected (O3‐N8‐O1‐C3 179.5(3)°; N8‐O1‐C3‐C2 175.1(2)°). The molecular units within a pair always arrange themselves in an alternating manner, i. e. they are rotated 180 ° to each other and thus a nitratoethyl rest is always superimposed with the azido function of its partner molecule.

**Figure 8:**
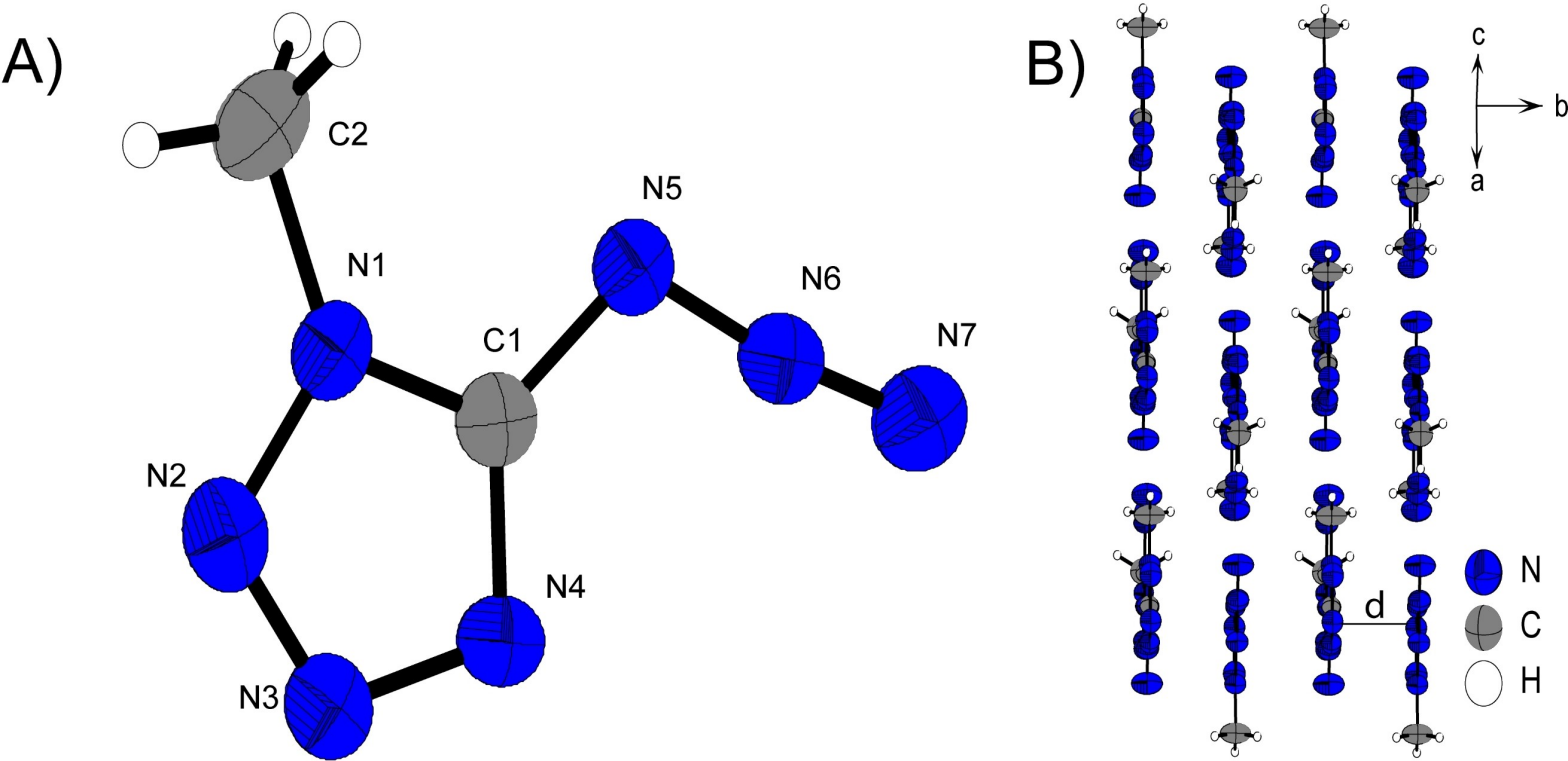
A) Molecular unit of **11** with thermal ellipsoids drawn at the 50 % probability level. Selected bond distances (Å) and angles [°]: C1‐N5 1.392(3), N5‐N6 1.253(2), N6‐N7 1.113(3), N1‐C2 1.455(3), N2‐N1‐C2 121.2(2), C1‐N1‐C2 131.4(2), N5‐N6‐N7 172.5(2), N6‐N5‐C1‐N4 0.0(2); B) Layer structure of **11**.

The 1‐ and 2‐methyl substituted 5‐azidotetrazoles both crystalize in monoclinic systems, *P*2_1_/*m* for **11** and *P*2_1_/*c* for **12**. This also results in similar three‐dimensional arrangements for both compounds. As shown in Figure 8 B) and Figure 7 B), layers are formed in the illustrated orientations. The alignment to layers is preferred firstly due to the planar character of **11** and **12**, and secondly as a result of the two hydrophobic azide and methyl substituents that do not form a good docking site for pronounced intermolecular interactions. The distances between the respective layers of the two 5‐azido‐methyltetrazoles are in the same range (d(**11**)=3.12 Å, d(**12**)=3.31 Å).

**Figure 9:**
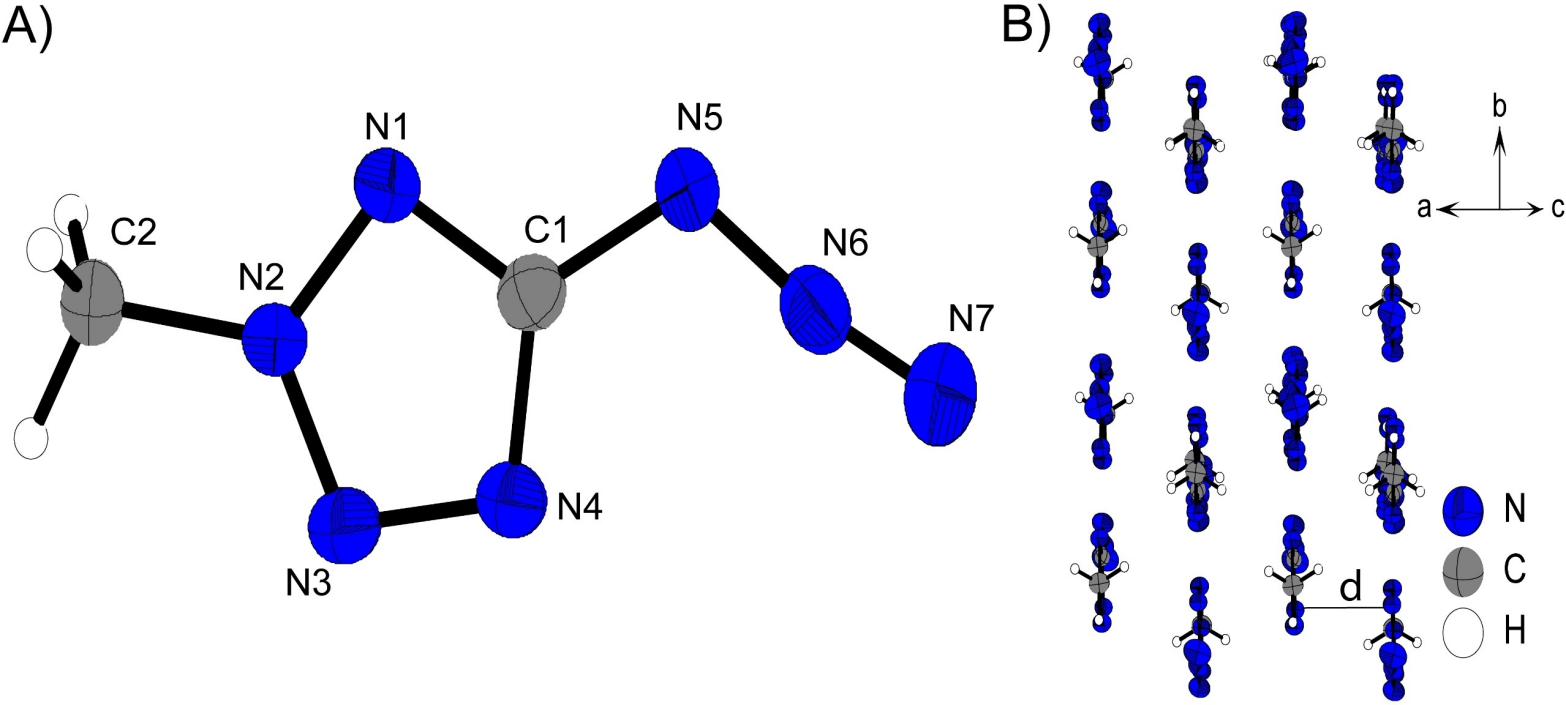
A) Molecular unit of **12** with thermal ellipsoids drawn at the 50 % probability level. Selected bond distances (Å) and angles [°]:C1‐N5 1.394(2), N5‐N6 1.259(2), N6‐N7 1.113(2), N2‐C2 1.448(2), N1‐N2‐C2 123.0(1), C2‐N2‐N3 123.1(1), N5‐N6‐N7 171.5(2), N6‐N5‐C1‐N4 1.6(2); B) Layer structure of **12**.

Despite the high structural conformity, the ordering of the molecular moieties within one layer changes as a result of the different substitution positions. **12** forms linear chains along the orientation depicted in Figure 9 B), with the azide group facing the methyl group with its γ‐nitrogen (N7), and is therefore tightly embraced by these protons. Since this configuration of molecular units in **12** is more space saving then in the molecular pattern of **11**, significant differences arise in the density of the isomers. **11** comes up with a density of 1.459 g cm^−3^ at 298 K whereas, **12** is about 0.08 g cm^−3^ more dense then its respective one substituted isomer (ρ(**12**)=1.542 g cm^−3^).

## NMR Spectroscopy

All compounds were characterized through ^1^H and ^13^C{^1^H} NMR spectroscopy and the respective resonances are listed in Table 1.

**Table 1:** ^1^H and ^13^C NMR resonances for compounds **2**, **2** **a**–**2** **d**, **7** and **10–12** measured in DMSO−D6, chemical shifts are reported in ppm with respect to TMS (Si(CH_3_)_4_).

Compound	*δ* [ppm]	
	^1^H	^13^C
**2**	9.69, 4.94, 3.97	157.3, 53.0, 48.9
**2** **a**	7.17, 4.69, 3.86	168.3, 51.6, 49.2
**2** **b**	10.03, 4.70, 3.87	168.0, 51.7, 49.2
**2** **c**	4.69, 3.87	169.0, 51.9, 49.6
**2** **d**	4.81, 3.93	166.5, 52.3, 49.0
**7**	4.20, 3.74	152.8, 49.1, 45.6
**10**	5.02, 4.68	152.9, 69.8, 43.8
**11**	3.77	152.9, 33.1
**12**	4.28	161.9, 40.7

Resonances for the azidoethyl function at position 2 in compounds **2** and **2** **a**–**2** **d** can be found in the range of 4.69‐4.94 ppm as multiplets (^1^H) and 51.6‐53.0 ppm (^13^C) representing the CH_2_‐group linked to the tetrazole ring and at 3.86‐3.97 ppm as multiplets (^1^H) and 48.9–49.6 ppm (^13^C) representing the CH_2_ protons bonded to N_α_. Additional proton signals for these compounds can be found for **2** (nitramine proton) as broad peak at *δ*=9.69 ppm and the cationic moieties for **2** **a** and **2** **b** at *δ*=7.17 and 10.03 ppm, respectively. The deprotonation of **2** results in a shift to deeper fields of the tetrazole carbon from 157.3 ppm for **2** by about 10 ppm for all ionic derivatives. For compounds **7** and **10** the tetrazole resonance is detected at 153 ppm in the ^13^C NMR. For the nitratoethyl function of **10** the resonance of the CH_2_ lined to the organic nitrate assigns at 5.02 ppm and the CH_2_ unit bonded to N1 at 4.68 ppm. The ethylene unit of **7** shows signals at 4.20 and 3.74 ppm.

For the methyl group signals of **11** (*δ*=3.77 ppm) and **12** (*δ*=4.28 ppm), the signal for the 2‐substitution is shifted to higher fields compared to the 1‐substituted one. The ^13^C shifts for **11** (δ(*C*N_4_)=152.9 ppm; δ(*C*H_3_)=33.1 ppm) and **12** (δ(*C*N_4_)=161.9 ppm; δ(*C*H_3_)=40.7 ppm) are in good accordance with other alkyl substituted 5‐azidotetrazoles.^[21]^

**Figure 10:**
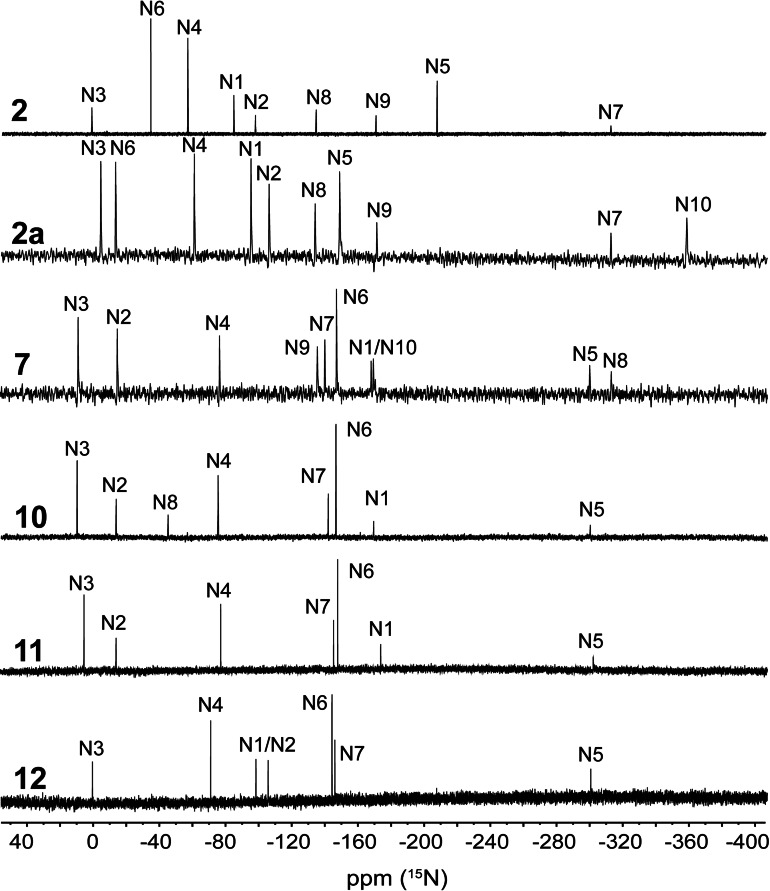
^15^N NMR spectra of compounds **2**, **2** **a**, **7** and **10–12**. Chemical shifts are reported in ppm with respect to MeNO_2_. Measurements of **2**, **2** **a** and **11–12** were performed in DMSO‐D_6_, for **7** and **10** in CDCl_3_ and in acetone‐D_6_, respectively.

Additionally, compounds **2**, **2** **a** (as representative for the 2‐(2‐azidoethyl)‐5‐nitraminotetrazolate anion), **7** and **10–12** were analyzed through proton coupled ^15^N spectroscopy. The spectra are illustrated in Figure 10. The nitrogen atoms are named as in the corresponding crystal structures. For the tetrazole moieties, signals for all four nitrogen atoms can be found in very similar ranges, depending on the substitution site (1‐ and 2‐substituted). For 1‐substituted tetrazoles **7**, **10** and **11** signals appear at around 0 ppm for N3, −13 ppm for N2, −75 ppm for N4 and −170 ppm for N1. For two substituted isomers **2**, **2** **a** and **12**, the chemical shifts for N3 and N4 do not differ hardly compared with 1‐substituted isomers. Values for N1 and N2 can be found around −90 ppm and −102 ppm, respectively.

The same applies to the azide functionalities. Here, however, a distinction must be made between aromatic and alkyl azides. Resonances for aromatic azides can be found in the spectra of compounds **7** and **10–12** in typical ranges of around −300 ppm for N_α_, −145 ppm for N_β_ and −143 ppm for N_γ_, whereas N_β_ having the highest intensity, followed by N_γ_ and N_α_ being the less intensive signal. Compared to the aromatic azides, alkyl azide resonances appear high‐field shifted at around −315 ppm for N_α_, −171 ppm for N_β_ and −135 ppm for N_γ_ with equal intensities. These signals are detectable for compounds **2**, **2** **a** and **7**. In the case of aromatic azides, the signals of N_β_ and N_γ_ appear relatively close together, whereas for alkyl azides, there is a clear gap of around 35 ppm between the two signals. N_γ_ always emerges at higher fields than N_β_.

Additional signals can be found in the spectra of **2**, **2** **a** and **10**. These are the nitro group of the protonated nitramine function (N6) of **2** at −34.7 ppm, the nitro group of the deprotonated nitramine function (N6) in **2** **a** at −13.9 ppm and the organic nitrate (N8) of **10** at −13.3 ppm.

## Physico‐chemical Properties

Compounds **2**, **2** **a**–**2** **d**, **7** and **10**–**12** were tested toward the behavior on certain external stimuli as there are impact, friction, electrostatic discharge and temperature (DTA or DSC). The resulting values are listed in Table 2. All investigated compounds can be declined as energetic materials, since they are more or less sensitive facing those external stimuli, according to UN Recommendations on the Transport of Dangerous Goods.^[22]^ The free acid of 2‐azidoethyl‐5‐nitraminotetrazole **2** is insensitive toward friction and shows an impact sensitivity of 10 J. The two nitrogen‐rich salts (**2** **a** and **2** **b**) are moderately sensitive, whereas the two metal‐containing salts (**2** **c** and **2** **d**) are already classified as extremely sensitive with **2d** being the most sensitive compound of the 2‐azidoethyl‐5‐nitramino derivatives (IS<1 J, FS=15 N). All 5‐azidotetrazole derivatives (**7**, **10**–**12**) show impact sensitivities less or equal to 1 J and friction sensitivities of 5 N (for **10**) or below for **7**, **11** and **12** and are therefore classified as sensitive explosives. Metal containing ionic derivatives **2** **c** and **2 d** are the thermally most stable compounds in this study decomposing at 180 °C and 181 °C, respectively. Nitrogen‐rich derivatives **2** **a** and **2** **b** decompose around 170 °C, whereby **2** **a** shows a smooth melting point at 113 °C. The free nitramine **2** is even liquid after long time storage −30 °C and explodes violently at 93 °C. 5‐Azidotetrazole derivatives **7** and **10**–**12** all undergo decomposition between 160 °C and 167 °C and possess a melting point in advance. Compound **7** is liquid at room temperature and melts at about −20 °C, **10**, **11** and **12** are solids at ambient temperatures and melt at 45 °C, 20 °C and 62 °C, respectively. Based on the densities and the calculated enthalpies of formation, the detonation parameters for all metal‐free were calculated using the EXPLO5 code. All investigated compounds show highly positive values for their heat of formation, with **9**, containing two azido moieties showing the overall highest heat of formation of Δ_f_H^0^=932.7 kJ mol^−1^. The calculated detonation velocity for **2** **b** is 8796 m s^−1^ and therefore in the range of RDX (*V*_det_=8801 m s^−1^). The performance parameters of the corresponding ammonium derivative as well as of the neutral compound are significantly lower, since they have both lower density and enthalpy of formation. Ethyl substituted 5‐azidotetrazoles **7** and **10** differ about 270 m s^−1^ is their detonation velocity, whereas nitratoethyl derivative **10** has the higher value with 8165 m s^−1^. This trend is in accordance with recently published ethyl substituted 5,5’‐azobistetrazoles.^[9a]^ In terms of detonation parameters, two methyl substituted 5‐azidotetrazole (**12**) is clearly superior to its one substituted isomer **11**. The detonation velocities are 7616 m s^−1^ for **11** and 7916 m s^−1^ for **12** and thus exactly 300 m s^−1^ apart, which is mainly attributed to the different densities. Unfortunately, the calculated detonation parameters fell short of expectations due to the low densities of the compounds. Both 5‐azidotetrazole backbones and azidoethyl substitutions yield enormous increases in the calculated formation detonation parameters, but the nonpolar properties of the substituents offer only limited opportunities for attractive interactions.

**Table 2:** Energetic properties and detonation parameters of **2**, **2** **a**–**2** **d**, **7** and **10–12**.

	**2**	**2** **a**	**2** **b**	**2** **c**	**2** **d**	7*	**10**	**11**	**12**
Formula	C_3_H_5_N_9_O_2_	C_3_H_8_N_10_O_2_	C_3_H_8_N_10_O_3_	C_3_H_4_N_9_O_2_K	C_3_H_4_N_9_O_2_Ag	C_3_H_4_N_10_	C_3_H_4_N_8_O_3_	C_2_H_3_N_7_	C_2_H_3_N_7_
M [g mol^−1^]	199.13	216.17	232.16	237.22	305.99	180.14	200.12	125.11	125.11
IS [J]^[a]^	10	30	9	1	<1	<1	1	<1	<1
FS [N]^[b]^	360	120	40	30	15	<0.1	5	<5	<5
ESD [mJ]^[c]^	–	100	100	25	13	–	10	50	80
[g cm^−3^]^[d]^	1.60*	1.575	1.624	1.93*	2.362	1.519	1.638	1.459	1.542
N [%]^[e]^	62.4	64.8	60.3	53.1	41.2	77.8	56.0	78.4	78.4
Ω [%]^[f]^	−28.1/−52.2	−37.0/−59.2	−27.6/−48.2	−20.2/−40.5	−15.7/−31.4	−44.4/−71.1	−16.0/−40.0	−44.8/−70.3	−44.8/−70.3
*T*_melt_/*T*_dec_ [°C]^[g]^	n.d./93	113/172	‐/171	126/180	‐/181	−20/167	45/166	20/160	62/162
Δ_f_ *H* ^0^ [kJ mol^−1^]^[h]^	647.1	649.1	705.5	–	–	932.7	514.5	594.5	566.9
Δ_f_ *U* ^0^[kJ mol^−1^]^[i]^	666.9	673.9	731.5	–	–	950.1	533.1	606.9	579.3
									
Explo5 V6.05.02									
−Δ_Ex_ *U* ^0^ [kJ kg^−1^]^[j]^	5282	5199	6281	–	–	5123	5409	4769	4563
*T*_det_ [K]^[k]^	3650	3321	3845	–	–	3535	3872	3383	3199
*V*_0_ [L kg^−1^]^[l]^	833	889	889	–	–	802	809	817	810
*P*_CJ_ [kbar]^[m]^	248	256	295	–	–	215	261	202	216
*V*_det_ [m s^−1^]^[n]^	8138	8430	8796	–	–	7898	8165	7616	7916

[a] Impact sensitivity (BAM drop hammer (1 of 6)). [b] Friction sensitivity (BAM friction tester (1 of 6)). [c] Electrostatic discharge device (OZM research). [d] From X‐Ray diffraction analysis recalculated to 298 K; *pycnometric measurement. [e] Nitrogen content. [f] Oxygen balance with respect to CO/CO2 [g] Decomposition temperature (DTA/DSC; β=5 °C min^−1^). [h] Calculated enthalpy of formation. [i] Calculated energy of formation. [j] Energy of explosion. [k] Detonation temperature. [l] Volume of detonation products (assuming only gaseous products). [m] Detonation pressure at Chapman–Jouguet point. [n] Detonation velocity. *Explo5 V6.05.02 calculation for the theoretical solid state at room temperature.

## Conclusion

By introducing azidoethyl, nitratoethyl and methyl groups to 5‐azidotetrazoles, the physico‐chemical properties could be specifically modified. Nitratoethyl shows a lower sensitivity and a higher melting point (45 °C) with better performance (8165 m s^−1^) then the respective azidoethyl homologue. Furthermore, the methyl functionalization at N2 not only outperforms its N1‐isomer in terms of physico‐chemical properties but also shows a much easier synthesis. The melting points of the isomers differ by 40 °C with the 2‐isomer melting at 62 °C. The decomposition temperatures of the 5‐azides are not altered by functionalization. The sensitivities are all in the area of primary explosives with 1‐(2‐azidoethyl)‐5‐azidotetrazole (**7**) being the most sensitive compound which is unusual because this compound is a liquid.

With the synthesis of 2‐(2‐azidoethyl)‐5‐nitraminotetrazole (**2**) and its nitrogen rich ionic derivatives (**2** **a**, **b**) new highly energetic secondary explosives were discovered. The hydroxylammonium salt **2** **b** owns the best explosive performance in this study with a detonation velocity of about 8800 m s^−1^. The metal salts (**2** **c**, **d**) are more sensitive explosives and as all ionic derivatives in this work stable up to 180 °C.

## Conflict of interest

The authors declare no conflict of interest.

1

## Supporting information

As a service to our authors and readers, this journal provides supporting information supplied by the authors. Such materials are peer reviewed and may be re‐organized for online delivery, but are not copy‐edited or typeset. Technical support issues arising from supporting information (other than missing files) should be addressed to the authors.

Supporting Information

## Data Availability

The data that support the findings of this study are available from the corresponding author upon reasonable request.
