# Supplementary material for: Tuning the Properties of 5‐Azido and 5‐Nitramino‐tetrazoles by Diverse Functionalization – General Concepts for Future Energetic Materials
Source: Chemistry. 2022 May 19;28(36):e202200772. doi: 10.1002/chem.202200772 (PMC9325492; doi:10.1002/chem.202200772)
Supplement: Supplementary file 1 — Supporting Information [file CHEM-28-0-s001.pdf]

Chemistry–A European Journal

Supporting Information

Tuning the Properties of 5-Azido and 5-Nitramino-tetrazoles by Diverse Functionalization – General Concepts for Future Energetic Materials

Maximilian Benz, Thomas M. Klapötke,* Tobias Lenz, and Jörg Stierstorfer

Supplementary Information

Table of Contents

1. Experimental part and general procedures
2. Thermal Analysis
3. X-ray diffraction
4. Computation
5. NMR Spectroscopy
6. References

1. Experimental part and general procedures

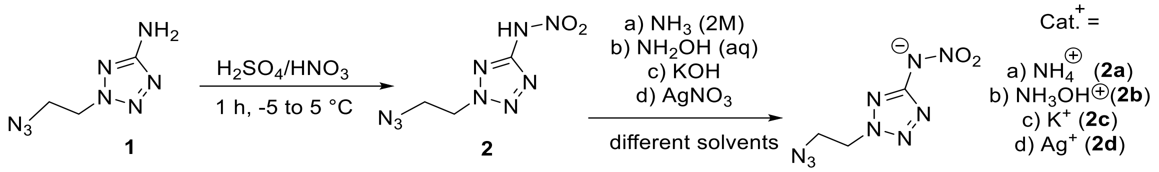

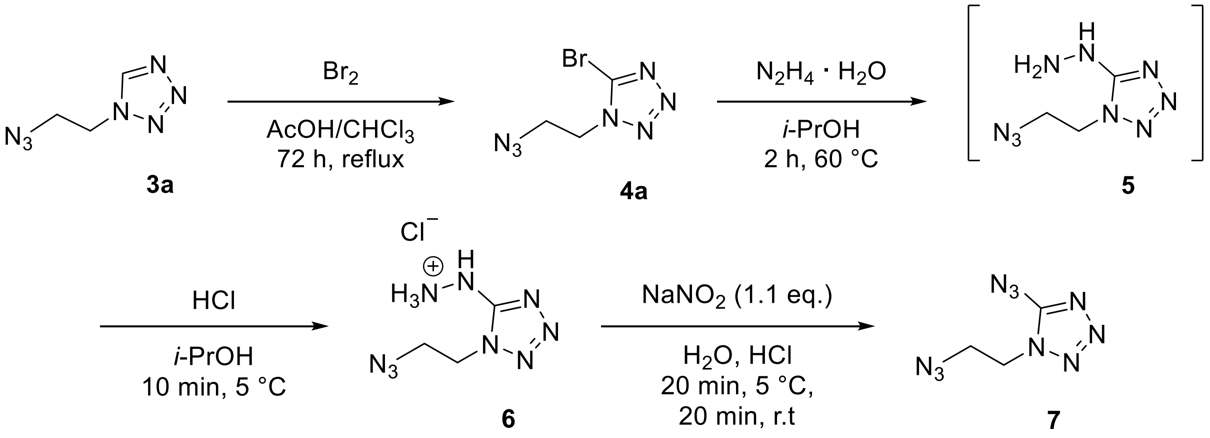

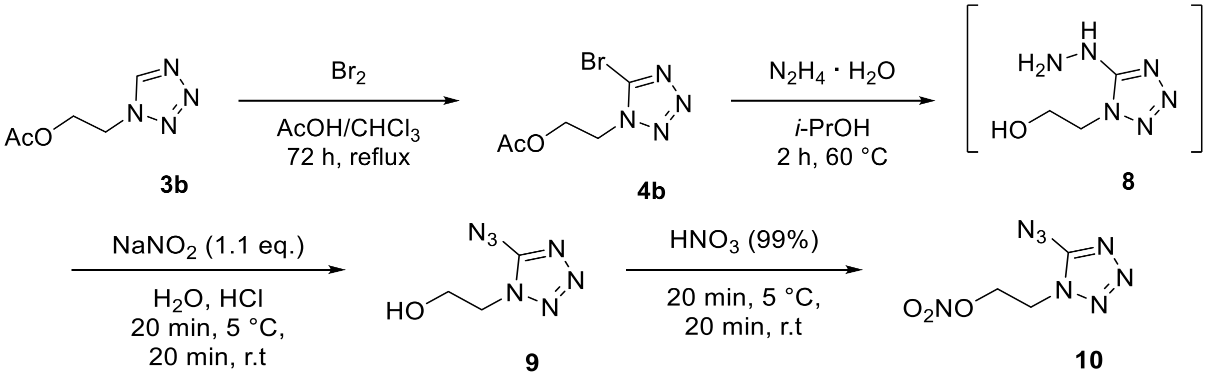

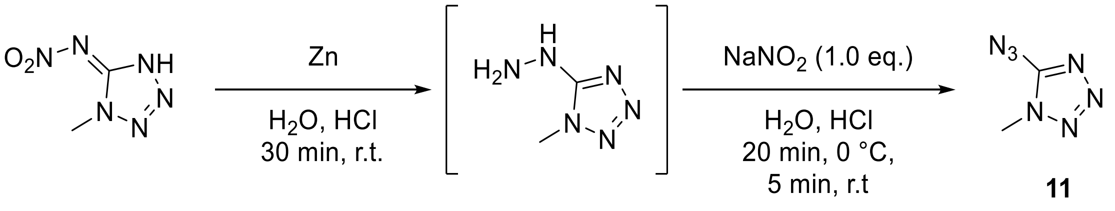

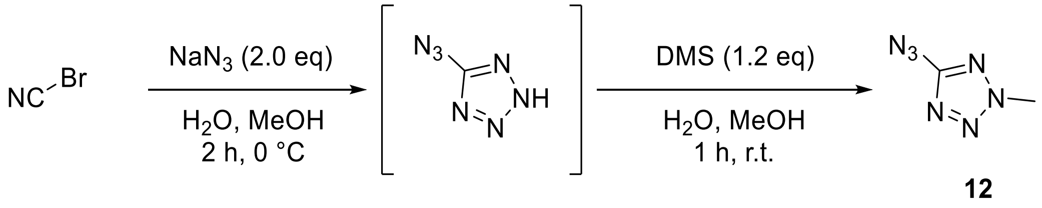

^{1}H, ^{13}C, ^{14}N and ^{15}N NMR spectra were recorded on *BRUKER AMX 400* instruments. Chemical shifts are referenced with respect to tetramethylsilane (^{1}H/^{13}C) and nitromethane (^{14}N/^{15}N). Infrared spectra (IR) were recorded in the region 4000-400 cm^{-1} on a *PERKIN ELMER Spectrum BX-59343* instrument with a *SMITHS DETECTION DuraSamplIR II Diamond ATR* sensor. The absorption bands are reported in wavenumbers (cm^{-1}). Decomposition temperatures were measured via differential thermal analysis (DTA) with an *OZM Research DTA 552-Ex* instrument at a heating rate of 5 °C/min and in a range of room temperature to 400 °C. All sensitivities toward impact (IS) and friction (FS) were determined according to BAM (Bundesanstalt für Materialforschung und Prüfung) standards using a BAM drop hammer and a BAM friction apparatus by applying the 1 of 6 method.[S1] All energetic compounds were tested for sensitivity towards electrical discharge using an *Electric Spark Tester ESD 2010 EN* from OZM. Energetic properties have been calculated with the EXPLO5 6.02 computer [S2] code using the RT converted X-ray density or the densities measured with a gas pycnometer at 298 K and calculated solid state heats of formation.

CAUTION! *All investigated compounds are potentially explosive materials. In particular compound **7**, 1-(2-azidoethyl)-5-azidotetrazole, is extremely sensitive and tends to explode during solidification. Safety precautions and equipment (such as wearing leather coat, face shield, Kevlar sleeves, Kevlar gloves, earthed equipment and ear plugs) must be used during all manipulations.*

2-(2-Azidoethyl)-5-nitraminotetrazole (2)[S3]

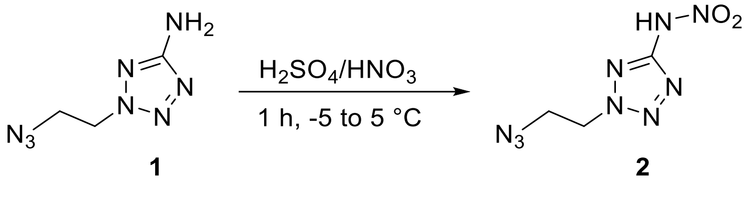

2-(2-Azidoethyl)-5-aminotetrazole (**1**) (1.00 g, 6.50 mmol, 1.0 eq) was dropwise added to a mixture of sulfuric acid (96%, 8.00 mL) and nitric acid (100%, 3.00 mL) keeping the temperature below 5 °C (*CAUTION!* By rising the temperature above 5 °C, the reaction mixture decomposes exothermically including the formation of large amount of nitrous gases, heat and flames in the reaction flask). The mixture was stirred for 1 h and was allowed to heat to 5 °C. The mixture was poured on ice water (150 mL) and extracted with diethyl ether (3 x 50 mL). After drying over anhydrous sodium sulfate,

the organic solvent was evaporated, the title compound **2** was obtained as slightly brownish liquid (1.16 g, 5.60 mmol, 86%).

DTA (5 °C min^{-1}): 93 °C (dec); Sensitivities: BAM drop hammer: 10 J (liquid), friction tester: 360 N (liquid), ESD: -; IR (ATR) $\tilde{\nu}$ (cm^{-1}) = 3023(w), 2102(s), 1700(w), 1610(s), 1488(m), 1440(m), 1299(vs), 1227(s), 1097(m), 1029(m), 893(m), 827(m), 758(m), 632(m), 554(m), 493(s), 445(m), 435(m), 428(m), 419(m), 412(m); Elem. Anal. ($C_3H_5N_9O_2$, 199.13 g mol^{-1}) calcd.: C 18.09, H 2.53, N 63.31 %. Found: C 17.94, H 2.82, N 62.37 %; ^{1}H NMR (DMSO-D$_6$, 400 MHz, ppm) δ = 9.69 (br s, 1H), 4.94 (m, 2H), 3.97 (m, 2H); ^{13}C NMR (DMSO-D$_6$, 101 MHz, ppm) δ = 157.3, 53.0, 48.9; ^{15}N NMR (DMSO-D$_6$, 41 MHz, ppm) δ = 1.0, −34.7, −57.1, −85.0, −98.1, −134.9, −171.2, −208.2, −313.5 ; HRMS (ESI) *m/z*: [M – H$^+$] Calcd for $C_3H_4N_9O_2$ 198.0493; Found: 198.0492.

Ammonium 2-(2-azidoethyl)-5-nitraminotetrazolate (2a)

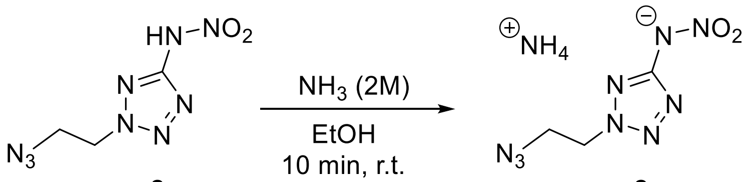

2 **2a**

2-(2-Azidoethyl)-5-nitraminotetrazole (**2**) (0.69 g, 3.47 mmol, 1.0 eq) was dissolved in ethanol (25 mL) and ammonia (2M dissolved in ethanol, 1.75 mL, 3.50 mmol, 1.0 eq) was added dropwise. The solvent was reduced and the precipitate was filtered to yield pure ammonium 2-(2-azidoethyl)-5-nitraminotetrazolate (**2a**) (0.66 g, 3.06 mmol, 88%) as brown solid.

DTA (5 °C min^{-1}): 113 °C (melt), 172 °C (dec); Sensitivities: BAM drop hammer: 30 J (≤ 500 µm), friction tester: 120 N (≤ 500 µm), ESD: 0.1 J (≤ 500 µm). IR (ATR) $\tilde{\nu}$ (cm^{-1}) = 3194(s), 2120(s), 1480(s), 1463(s), 1452(s), 1434(s), 1397(s), 1367(s), 1343(s), 1294(vs), 1240(s), 1204(s), 1168(s), 1097(s), 1059(m), 1036(s), 1013(s), 974(s), 953(m), 888(s), 833(s), 771(s), 757(s), 742(m), 699(s), 675(m), 646(m), 560(m), 512(s), 467(s), 448(m); Elem. Anal. ($C_3H_8N_{10}O_2$, 216.17 g mol^{-1}) calcd.: C 16.67, H 3.73, N 64.80 %. Found: C 16.75, H 3.80, N 63.48 %; ^{1}H NMR (DMSO-D$_6$, 400 MHz, ppm) δ = 7.17 (s, 4H), 4.69 (m, 2H), 3.86 (m, 2H); ^{13}C NMR (DMSO-D$_6$,

101 MHz, ppm) *δ* = 168.3, 51.6, 49.2; ^{15}N NMR (DMSO-D$_6$, 41 MHz, ppm) *δ* = −5.0, −13.9, −61.4, −95.6, −106.5, −134.3, −149.0, −171.4, −312.7, −358.4.

Hydroxylammonium 2-(2-azidoethyl)-5-nitraminotetrazolate (2b)

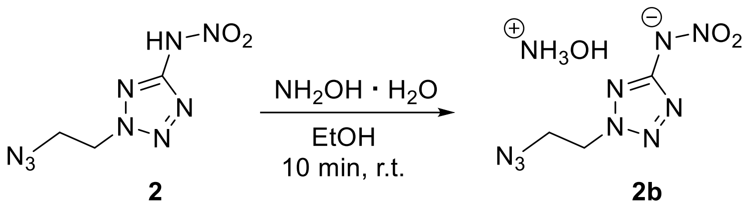

2 **2b**

2-(2-Azidoethyl)-5-nitraminotetrazole (**2**) (0.87 g, 4.47 mmol, 1.0 eq) was dissolved in ethanol (18 mL) and aqueous hydroxylamine solution (50% w/w, 0.28 mL, 4.70 mmol, 1.05 eq) was added dropwise. The solvent was reduced and the precipitate was filtered to yield pure hydroxylammonium 2-(2-azidoethyl)-5-nitraminotetrazolate (**2b**) (0.85 g, 3.66 mmol, 82%) as yellow solid.

DTA (5 °C min^{-1}): 171 °C (dec); Sensitivities: BAM drop hammer: 9 J (≤ 500 µm), friction tester: 40 N (≤ 500 µm), ESD: 0.1 J (≤ 500 µm). IR (ATR) $\tilde{\nu}$ (cm^{-1}) = 3112(m), 2691(m), 2141(s), 2102(s), 1485(s), 1437(s), 1406(s), 1358(s), 1341(vs), 1290(vs), 1238(s), 1213(s), 1169(vs), 1119(s), 1101(s), 1038(s), 1009(s), 953(m), 897(m), 884(m), 839(m), 775(s), 751(s), 695(m), 688(m), 669(s), 626(s), 555(s), 502(s), 456(s), 441(s); Elem. Anal. ($C_3H_8N_{10}O_3$, 232.16 g mol^{-1}) calcd.: C 15.52, H 3.47, N 60.33 %. Found: C 14.90, H 3.63, N 59.85 %; ^{1}H NMR (DMSO-D$_6$, 400 MHz, ppm) *δ* = 10.03 (br s, 4H), 4.70 (m, 2H), 3.87 (m, 2H); ^{13}C NMR (DMSO-D$_6$, 101 MHz, ppm) *δ* = 168.0, 51.7, 49.2; ^{14}N NMR (DMSO-D$_6$, 29 MHz, ppm) *δ* = −3, −13, −134, −173, −359.

Potassium 2-(2-azidoethyl)-5-nitraminotetrazolate (2c)

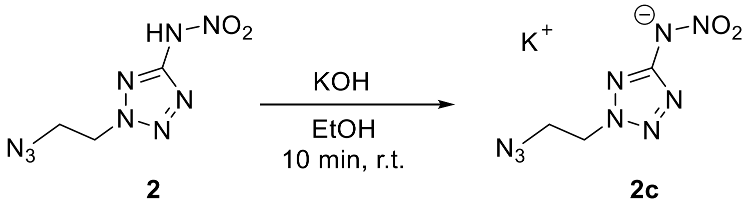

2 **2c**

2-(2-Azidoethyl)-5-nitraminotetrazole (**2**) (0.85 g, 4.24 mmol, 1.0 eq) was dissolved in ethanol (18 mL) and potassium hydroxide (0.24 g, 4.24 mmol, 1.0 eq) dissolved in ethanol (8 mL) was added in one portion. The immediately formed solid was filtered

and washed with little amount of cold ethanol to yield potassium 2-(2-azidoethyl)-5-nitraminotetrazolate (**2c**) (0.90 g, 3.80 mmol, 90%) as brownish solid.

DTA (5 °C min^{-1}): 126 °C (melt), 180 °C (dec); Sensitivities: BAM drop hammer: 1 J (≤ 500 µm), friction tester: 30 N (≤ 500 µm), ESD: 25 mJ (≤ 500 µm). IR (ATR) $\tilde{\nu}$ (cm^{-1}) = 2118(s), 2080(m), 1486(s), 1417(s), 1407(s), 1383(s), 1320(vs), 1299(vs), 1261(s), 1208(s), 1099(s), 1041(s), 1006(s), 971(m), 944(m), 883(m), 828(s), 777(s), 760(s), 696(m), 647(m), 557(m), 507(m), 463(m); Elem. Anal. ($C_3H_4N_8O_2K$, 237.22 g mol^{-1}) calcd.: C 15.19, H 1.70, N 53.14 %. Found: C 15.49, H 1.80, N 52.10 %; ^{1}H NMR (DMSO-D$_6$, 400 MHz, ppm) δ = 4.69 (m, 2H), 3.87 (m, 2H); ^{13}C NMR (DMSO-D$_6$, 101 MHz, ppm) δ = 169.0, 51.9, 49.6; ^{14}N NMR (DMSO-D$_6$, 29 MHz, ppm) δ = −12, −135, −173.

Silver 2-(2-azidoethyl)-5-nitraminotetrazolate (2d)

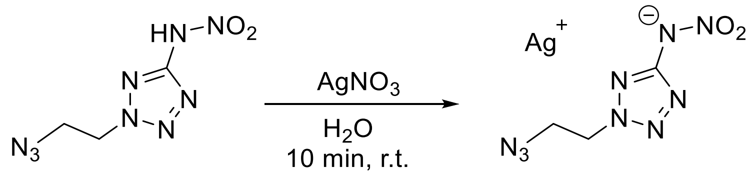

2-(2-Azidoethyl)-5-nitraminotetrazole (**2**) (0.29 g, 1.43 mmol, 1.0 eq) was dissolved in ethanol (5 mL) and silver nitrate (0.25 g, 1.45 mmol, 1.0 eq) dissolved in water (5 mL) was added in one portion. The immediately formed solid was filtered and washed with little amount of cold water to obtain silver 2-(2-azidoethyl)-5-nitraminotetrazolate (**2d**) (0.43 g, 1.40 mmol, 98%) in quantitative yield as beige powder.

DTA (5 °C min^{-1}): 181 °C (dec); Sensitivities: BAM drop hammer: <1 J (≤ 500 µm), friction tester: 15 N (≤ 500 µm), ESD: 13 mJ (≤ 500 µm). IR (ATR) $\tilde{\nu}$ (cm^{-1}) = 2138(s), 2101(s), 1509(s), 1438(s), 1406(s), 1357(s), 1343(s), 1286(vs), 1213(s), 1170(s), 1120(m), 1101(m), 1038(s), 1010(s), 952(m), 884(m), 752(m), 743(m), 694(m), 689(m), 670(s), 626(m), 555(m), 503(m), 456(m), 441(m); Elem. Anal. ($C_3H_4N_8O_2Ag$, 305.99 g mol^{-1}) calcd.: C 11.78, H 1.32, N 41.20 %. Found: C 11.77, H 1.51 N 40.29 %; ^{1}H NMR (DMSO-D$_6$, 400 MHz, ppm) δ = 4.81 (m, 2H), 3.93 (m, 2H); ^{13}C NMR (DMSO-D$_6$, 101 MHz, ppm) δ = 166.5, 52.3, 49.0; ^{14}N NMR (DMSO-D$_6$, 29 MHz, ppm) δ = −16, −135.

1-(2-Azidoethyl)-5-bromotetrazole (4a)

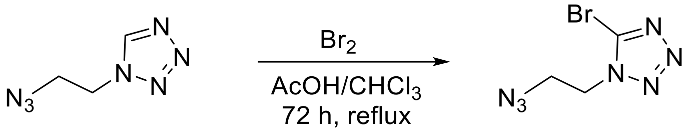

3a **4a**

To a solution of 1-(2-azidoethyl)-tetrazole (**3a**) (5.00 g, 35.9 mmol, 1.0 eq) in acetic acid (35 mL) and chloroform (70 mL) was added bromine (11.5 g, 71.8 mmol, 2.0 eq) in chloroform (15 mL). After 72 h at reflux the solvent was evaporated and excess saturated sodium bicarbonate solution was added to the residue. The mixture was extracted with ethyl acetate (3 x 100 mL), the solvent was dried over sodium sulfate and removed to yield 1-(2-azidoethyl)-5-bromotetrazole (**4a**) (6.60 g, 30.3 mmol, 84%) as yellow liquid.

Sensitivities: BAM drop hammer: 40 J (liquid), friction tester: >360 N (liquid); IR (ATR) $\tilde{\nu}$ (cm^{-1}) = 2099(vs), 1455(m), 1429(s), 1414(s), 1398(s), 1352(m), 1286(m), 1248(m), 1228(m), 1180(s), 1123(m), 981(w), 826(w), 663(m), 647(m), 631(m), 494(m); Elem. Anal. ($C_3H_4N_7Br$, 218.02 g mol^{-1}) calcd.: C 16.53, N 44.97, H 1.85%. Found: C 16.40, N 43.27, H 2.00%; ^{1}H NMR (DMSO-D_6, 400 MHz, ppm) δ = 4.63 – 4.59 (m, 2H), 3.93 – 3.86 (m, 2H).^{13}C NMR (DMSO-D_6, 101 MHz, ppm) δ = 135.0, 49.1, 42.5; HR-MS (ESI, 70 eV): [$C_3H_5N_7Br_2$] calcd.: 297.8879 [M – Br^-], found: 297.8882.

5-Bromo-1-(2-acetoxyethyl)-tetrazole (4b)[S4]

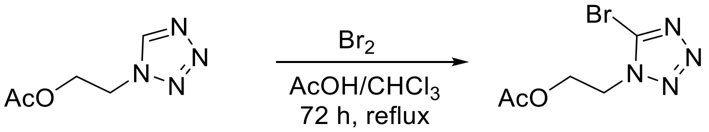

3b **4b**

To a solution of 1-(2-acetoxyethyl)-tetrazole (**3b**) (4.1 g, 26 mmol, 1.0 eq) in acetic acid (25 mL) and chloroform (50 mL) was added bromine (2.7 ml, 8.4 g, 52 mmol, 2.0 eq) in chloroform (10 mL). After 72 h at reflux the solvent was evaporated and excess saturated sodium bicarbonate solution was added to the residue. The mixture was extracted with ethyl acetate (3 x 50 mL), the solvent was dried over sodium sulfate and removed to yield 5-bromo-1-(2-chloroethyl)-tetrazole (**4b**) (5.8 g, 25 mmol, 95%) as yellow liquid.

^{1}H NMR (DMSO-D$_6$, 400 MHz, ppm) δ = 4.69 (m, 2H), 4.44 (m, 2H), 1.96 (s, 3H); ^{13}C NMR (DMSO-D$_6$, 101 MHz, ppm) δ = 170.0, 135.0, 61.2, 47.4, 20.4;

1-(2-Azidoethyl)-5-hydraziniumtetrazole chloride (6)

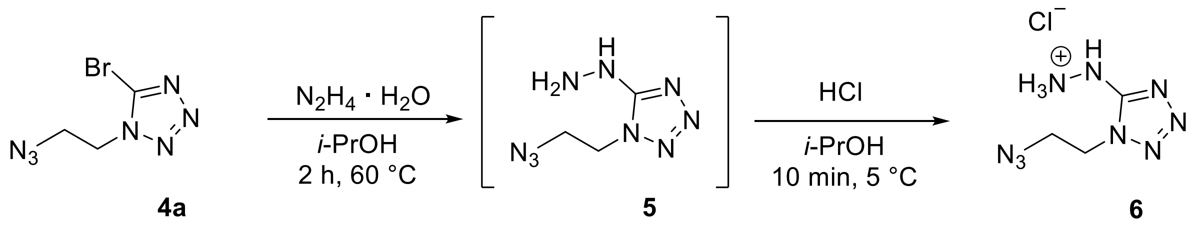

To a solution of 1-(2-azidoethyl)-5-bromotetrazole (**4a**) (1.00 g, 4.57 mmol, 1.0 eq.) in 2-propanole (20 mL) was added hydrazine hydrate (100%, 0.66 ml, 13.70 mmol, 3.0 eq.). After 2 h at 60 °C the solvent was removed and the residue was dissolved in water (50 mL) and extracted with ethyl acetate (3 x 50 mL). The organic phase was dried over sodium sulfate and evaporated to yield a crude oil of 1-(2-azidoethyl)-5-hydrazineyltetrazole (**5**) that forms colorless crystals over time. (^{1}H NMR (DMSO-D$_6$, 400 MHz, ppm) δ = 8.14 (s, 1H), 4.48 (s, 2H), 4.44 – 4.37 (m, 2H), 3.78 – 3.68 (m, 2H). ^{13}C NMR (DMSO-D$_6$, 101 MHz, ppm) δ = 158.3, 48.9, 44.9; HR-MS (ESI, 70 eV): [$C_3H_8N_9$] calcd.: 170.0898 [M – H$^+$], found: 170.0899. The residue was then dissolved in 2-propanol (10 mL) and an excess of a solution of HCl in 2-propanol (4-6 N, 3 mL) was added. After full precipitation and crystallization at 5 °C over night the product was filtered and washed with ether to yield white solid 1-(2-azidoethyl)-5-hydraziniumtetrazol chloride (**6**) (498 mg, 2.42 mmol, 53%).

Sensitivities: BAM drop hammer: >40 J (≤ 500 µm), friction tester: >360 N (≤ 500 µm); IR (ATR) $\tilde{\nu}$ (cm^{-1}) = 3204(m), 2788(s), 2646(s), 2593(s), 2138(s), 2096(vs), 1599(s), 1570(vs), 1548(s), 1494(s), 1444(s), 1421(s), 1370(m), 1343(s), 1331(m), 1300(s), 1278(s), 1253(m), 1184(m), 1125(m), 1102(s), 1063(m), 1009(s), 971(m), 827(s), 732(s), 667(m), 607(s), 534(s), 486(s); Elem. Anal. ($C_3H_8N_9Cl$, 205.61 g mol^{-1}) calcd.: C 17.52, H 3.92, N 61.31 %. Found: C 18.01, H 4.09, N 60.24 %; ^{1}H NMR (D_2O, 400 MHz, ppm) δ = 4.52 – 4.26 (m, 2H), 3.98–3.54 (m, 2H).^{13}C NMR (DMSO-D$_6$, 101 MHz, ppm) δ = 154.6, 48.9, 46.1.; ^{14}N NMR (DMSO-D$_6$, 29 MHz, ppm) δ = −135.4, −168.6; HR-MS (ESI, 70 eV): [$C_3H_7N_9Cl$] calcd.: 204.0518 [M – Cl$^-$], found: 204.05179.

1-(2-Azidoethyl)-5-azidotetrazole (7)

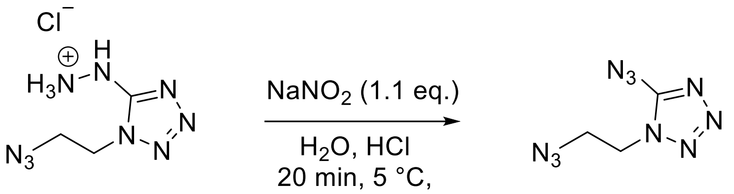

6 7

To a solution of 1-(2-azidoethyl)-5-hydraziniumtetrazol chloride (**6**) (200 mg, 0.97 mmol, 1.0 eq) in water (10 mL) and aqueous hydrochloric acid (2N, 2 mL) was added slowly sodium nitrite (74 mg, 1.07 mmol, 1.1 eq.) at 5 °C. After 20 min at 5 °C the solution was stirred at room temperature for further 20 min. Then the reaction was extracted with ether (3 x 20 mL) (using a plastic separation funnel and plastic vessels!). The organic layer was washed with water, dried over sodium sulfate and evaporated under a nitrogen stream to yield a yellowish oil of 5-azido-1-(2-azidoethyl)-tetrazole (**7**). The oil was purified by column chromatography (EtOAc/Ihex; 8/2; Rf: 0.35) to yield white **7** (265 mg, 1.71 mmol, 40%) (166 mg, 0.92 mmol, 95%) as highly sensitive colorless oil.

(*CAUTION!* The reaction and workup have to be performed very carefully with personal protection and blast shield as the product is highly sensitive. A violent detonation occurred while handling the azido tetrazole with a glass pipette. Therefore, plastic equipment is recommended.)

DTA (5 °C min^{-1}): 167 °C (dec.); Sensitivities: BAM drop hammer: <1 J (liquid), friction tester: <0.1 N (liquid). IR (ATR) $\tilde{\nu}$ (cm^{-1}) = 2156(s), 2101(s), 1531(vs), 1470(m), 1445(m), 1352(w), 1326(m), 1294(m), 1252(m),1226(m), 1181(m), 1125(w), 1089(m), 827(w), 723(m), 654(m), 636(w), 555(w), 528(m), 502(w), 436(w), 426(w); Elem. Anal. ($C_3H_4N_{10}$, 180.14 g mol^{-1}) calcd.: C 20.00, H 2.24, N 77.76 %. Found: not determinable; ^{1}H NMR (DMSO-D_6, 400 MHz, ppm) δ = 4.20 (m, 2H), 3.74 (m, 2H).^{13}C NMR (DMSO D_6, 101 MHz, ppm) δ 152.8, 49.1, 45.6; ^{15}N NMR ($CDCl_3$, 41 MHz, ppm) δ = 9.9, −13.9, −76.1, −135.7, −140.2, −147.3, −168.4, −169.7, −301.3, −314.4; HR-MS (ESI, 70 eV): [$C_3H_5N_{10}$] calcd.: 181.0694 [M – H^+], found: 181.0695.

5-Azido-1-(2-hydroxyethyl)-tetrazole (9)

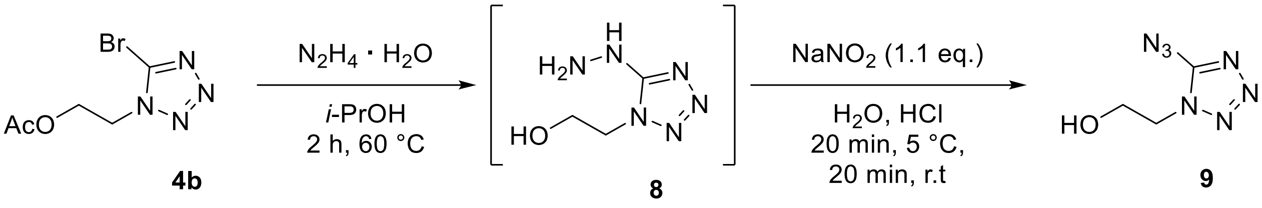

To a solution of 5-bromo-1-(2-acetoxyethyl)-tetrazole (**4b**) (1.00 g, 4.25 mmol, 1.0 eq.) in 2-propanole (25 mL) was added hydrazine hydrate (100%, 0.83 ml, 17.00 mmol, 4.0 eq.). After 72 h at ambient temperature, the precipitated crystals were removed by filtration. The solvent was evaporated to yield a crude mixture of 5-hydrazineyl-1-(2-hydroxyethyl)-tetrazole (**8**) and acetohydrazide. Without further purification the mixture was dissolved in hydrochloric acid (2M, 75 mL) and cooled to 0 – 5 °C. Slowly sodium nitrite (2.65 g, 38.2 mmol, 9.0 eq) in few water was added. After 20 min at 5 °C the solution was allowed to come to room temperature and was then extracted with ethyl acetate (3x50 mL). The organic layer was washed with water, dried over sodium sulfate and evaporated under a nitrogen stream to yield a crude oil of 5-azido-1-(2-hydroxyethyl)-tetrazole (**9**). The oil was purified by column chromatography (EtOAc/Ihex; 8/2; Rf: 0.35) to yield white crystalline **9** (265 mg, 1.71 mmol, 40%)

DTA (5 °C min^{-1}): 45 °C (melt.), 177 °C (dec.); IR (ATR) $\tilde{\nu}$ (cm^{-1}) = 2944(w), 2156(s), 2134(s), 2101(s), 1531(vs), 1470(m), 1445(m), 1352(w), 1326(m), 1252(m), 1226(m), 1181(m), 1125(w), 1089(m), 827(w), 808(w), 723(m), 654(m), 636(w), 555(w), 528(m); Elem. Anal. ($C_3H_5N_7O$, 155.12 g mol^{-1}) calcd.: C 23.23, H 3.25, N 63.21 %. Found: C 23.88, H 3.37, N 60.17 %.; ^{1}H NMR (DMSO-D_6, 400 MHz, ppm) δ = 5.05 (t, J = 5.7 Hz, 1H), 4.19 (dd, J = 5.7, 4.9 Hz, 2H), 3.74 (q, J = 5.5 Hz, 2H). ^{13}C NMR (DMSO-D_6, 101 MHz, ppm) δ = 152.6, 58.5, 49.2; ^{14}N NMR (DMSO-D_6, 29 MHz, ppm) δ = −133.8; HR-MS (EI, 70 eV): [$C_3H_5N_7O$] calcd.: 155.0556 [M^+], found: 155.0550.

5-Azido-1-(2-nitratoethyl)-tetrazole (10)

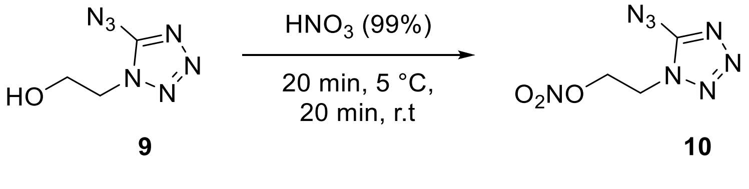

5-Azido-1-(2-nitratoethyl)-tetrazole (**9**) (150 mg,1.03 mmol) was added to white fuming nitric acid (99%, 2 mL) at 5°C. After 20 min the reaction was allowed to come to room

temperature and after further 20 min the reaction was quenched on ice. After extraction with ethyl acetate (3x20 mL) and neutralization with saturated sodium bicarbonate solution the organic layer was washed with water, dried over sodium sulfate and evaporated under a nitrogen stream to yield a crude oil of 5-azido-1-(2-nitratoethyl)-tetrazole (**10**). For purification the oil was dissolved in a small amount of methanol (5-10% water) and left for crystallization at 5°C to yield white crystalline **10** (173 mg, 0.87 mmol, 84%)

DTA (5 °C min^{-1}): 45 °C (melt.), 166 °C (dec.); Sensitivities: BAM drop hammer: <1 J, friction tester: 5 N; IR (ATR) $\tilde{\nu}$ (cm^{-1}) = 2967(w), 2923(w), 2158(s), 1745(w), 1635(s), 1534(vs), 1471(m), 1429(m), 1331(m), 1279(vs), 1185(m), 1090(m), 1027(m), 1006(m), 885(s), 840(vs), 754(m), 724(m), 705(m), 659(m), 633(m), 528(m); Elem. Anal. ($C_3H_4N_8O_3$, 200.12 g mol^{-1}) calcd.: C 18.01, H 2.01, N 55.99 %. Found: C 18.41, H 2.36, N 54.57 %.; ^{1}H NMR (Acetone-D_6, 400 MHz, ppm) δ = 5.02 (m, 2H), 4.68 (m, 2H); ^{13}C NMR (Acetone-D_6, 101 MHz, ppm) δ = 152.9, 69.8, 43.8; ^{15}N NMR (Acetone-D_6, 41 MHz, ppm) δ = 10.5, −13.3, −44.9, −75.3, −142.3, −147.0, −169.9, −301.7; HR-MS (EI, 70 eV): [$C_3H_5N_8O_3$] calcd.: 200.0406 [M^+], found: 200.0404.

5-Azido-1-methyltetrazole (11)

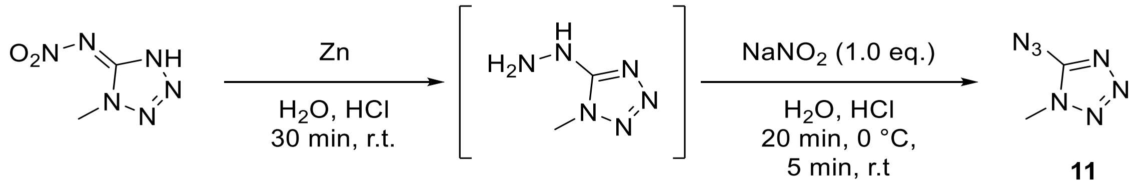

11

1-Methyl-5-nitriminotetrazole (1.44 g, 10.0 mmol, 1.0 eq) was dissolved in water (10 mL). To this, an excess of zinc powder (2.00 g) was added and the mixture was cooled using an ice bath. Hydrochloric acid (30 mL, 2 M) was added in drops and the mixture was stirred for 30 min at room temperature. After the remained zinc powder was removed by filtration a solution of sodium nitrite (0.65 g, 10.0 mmol, 1.0 eq) in water (10 mL) was added dropwise at temperatures below 0 °C until a clear formation of NO_2 was observed. After stirring the solution for further 5 min, the product was extracted DCM (3 x 20 mL). The organic phases were combined, washed once with hydrochloric acid (2 M) and two times with a concentrated solution of Na_2CO_3. The organic phase was dried using sodium sulfate and evaporated. Liquid **11** (0.78 g, 6.30 mmol, 63%) was remained which could be solidified by cooling in a freezer.

DSC (5 °C min^{-1}): 20 °C (melt.), 160 ° (dec.); Sensitivities: BAM drop hammer: <1 J; friction tester: <5 N; ESD: 50 mJ; EA ($C_2H_3N_7$, 125.11) calcd.: C 19.20, H 2.42, N 78.38 %; Found: C 19.30, H 2.81, N 78.00; IR (ATR) $\tilde{\nu}$ (cm^{-1}) = 2962 (w), 2160 (vs), 1630 (w), 1547 (s), 1476 (s), 1447 (m), 1413 (m), 1377 (w), 1304 (s), 1259 (s), 1221 (s), 1166 (w), 1092 (m), 1036 (w), 971 (w), 816 (w), 723 (m), 693 (w), 673 (s), 528 (w); ^{1}H NMR (DMSO-D$_6$, 400 MHz, ppm) δ = 3.77 (CH_3); ^{13}C NMR (DMSO-D$_6$, 101 MHz, ppm) δ = 152.9, 33.1; ^{15}N NMR (DMSO-D$_6$, 41 MHz, ppm) δ = 7.2 (N3), –12.2 (N2, q, $^3J_{NH}$ = 1.6 Hz), –75.6 (N4), –144.0 (N7), –146.5 (N6), –172.6 (N1, q, $^2J_{NH}$ = 2.0 Hz), –301.6 (N5); *m/z* (DEI): 83 (1), 58 (13), 53 (8), 43 (42), 42 (3), 40 (4), 32 (2), 28 (13), 15 (10).

5-Azido-2-methyltetrazole (12)

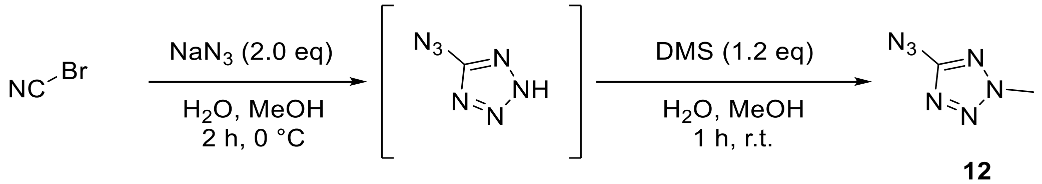

12

Cyanogen bromide (0.53 g, 5.00 mmol, 1.0 eq) was dissolved in a mixture of cold water (16 mL) and MeOH (4 mL). To this, a solution of sodium azide (0.65 g, 10.0 mmol, 2.0 eq) was added drop wise while cooling in an ice bath. After 2 h, dimethyl sulfate (0.28 mL, 3.0 mmol, 1.2 eq) was added slowly and the solution was allowed to come to room temperature. After one hour, the precipitate formed was filtered off and washed with a small amount of cold water. The analytically pure **12** (0.29 g, 2.30 mmol, 46%) can be recrystallized from hot water.

DSC (5 °C min^{-1}): 62 °C (melt), 162 °C (dec); Sensitivities: BAM drop hammer: <1 J friction sensitivity: <5 N; ESD: 80 mJ; EA ($C_2H_3N_7$, 125.11) calcd.: C 19.20, H 2.42, N 78.38 %; Found: C 19.19, H 2.21, N 77.41; IR (ATR) $\tilde{\nu}$ (cm^{-1}) = 3038 (w), 2956 (w), 2417 (w), 2285 (w), 2160 (vs), 1725 (w), 1588 (m), 1550 (s), 1506 (s), 1476 (s), 1421 (m), 1396 (m), 1305 (m), 1259 (w), 1218 (m), 1182 (m), 1091 (w), 1050 (w), 1026 (w), 794 (m), 739 (m), 721 (w), 674 (m), 527 (m); ^{1}H NMR (DMSO-D$_6$, 400 MHz, ppm): δ = 4.28 (CH_3); ^{13}C NMR (DMSO-D$_6$, 101 MHz, ppm): δ = 161.9, 40.7; ^{15}N NMR (DMSO-D$_6$, 41 MHz, ppm): δ = 0.4 (N1, q, $^3J_{NH}$ = 2.0 Hz), –71.0 (N4), –98.3 (N1, q, $^3J_{NH}$ = 1.9 Hz), –105.7 (N2, q, $^2J_{NH}$ = 2.3 Hz), –144.2 (N6), –146.0 (N7), –300.5 (N5); *m/z* (DEI):

125 (43), 83 (1), 69 (12), 57 (1), 55 (3), 54 (23), 53 (12), 43 (100), 40 (11), 29 (11), 28 (29), 27 (12), 26 (14), 31 (8), 28(29), 18 (14), 15 (48).

2. Thermal Analysis

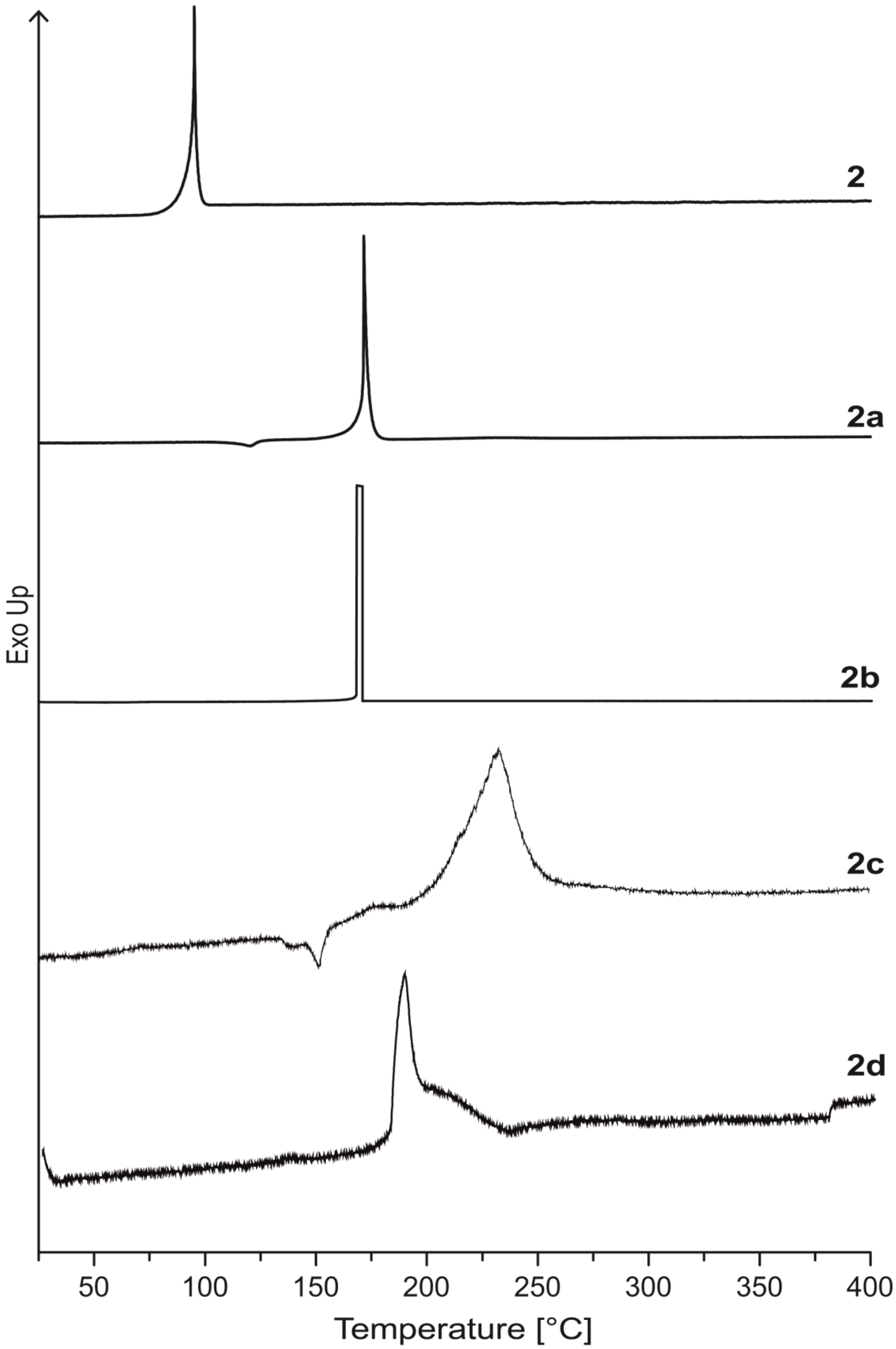

Figure S1. Thermal analysis through DTA of compounds **2** and **2a-2d**.

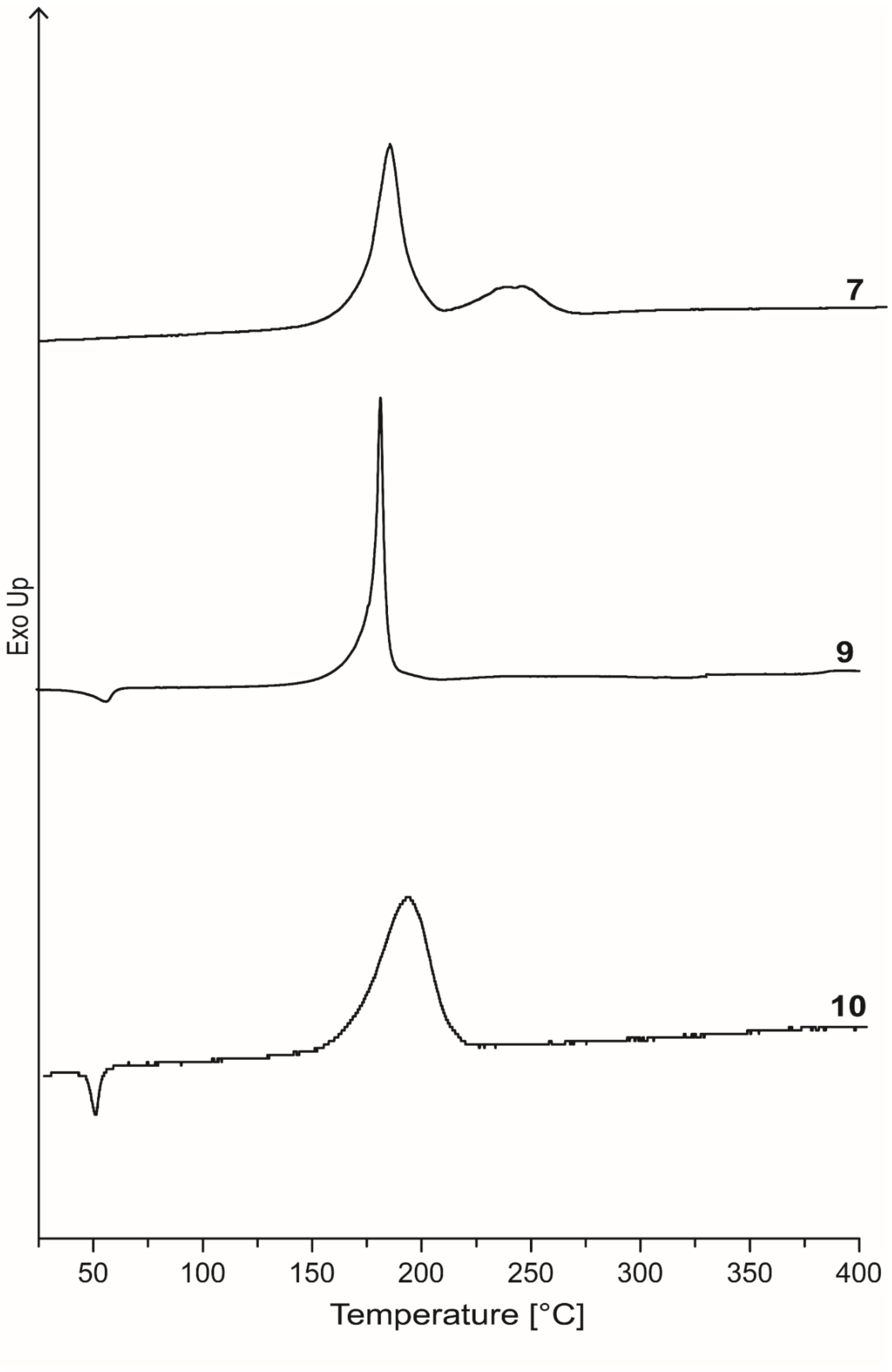

Figure S2. Thermal analysis through DTA of compounds **7** and **9**-**10**.

3. X-ray diffraction

Crystal structure data were obtained from an Oxford Xcalibur3 diffractometer with a Spellman generator (voltage 50 kV, current 40 mA) and a Kappa CCD area for data collection using Mo-*Kα* radiation (*λ* = 0.71073 Å). The data collection was performed using the CRYSTALIS RED software.[S5] The solution of the structure was performed by direct methods and refined by full-matrix least-squares on F2 (SHELXT)[S6] implemented in the OLEX2[S7] software suite. The non-hydrogen atoms were refined anisotropically and the hydrogen atoms were located and freely refined. The absorption correction was carried out by a SCALE3 ABSPACK multiscan method.[S8] The DIAMOND2 plots shown with thermal ellipsoids at the 50% probability level and hydrogen atoms are shown as small spheres of arbitrary radius. The SADABS program embedded in the Bruker APEX3 software was used for multi-scan absorption corrections in all structures.[S9]

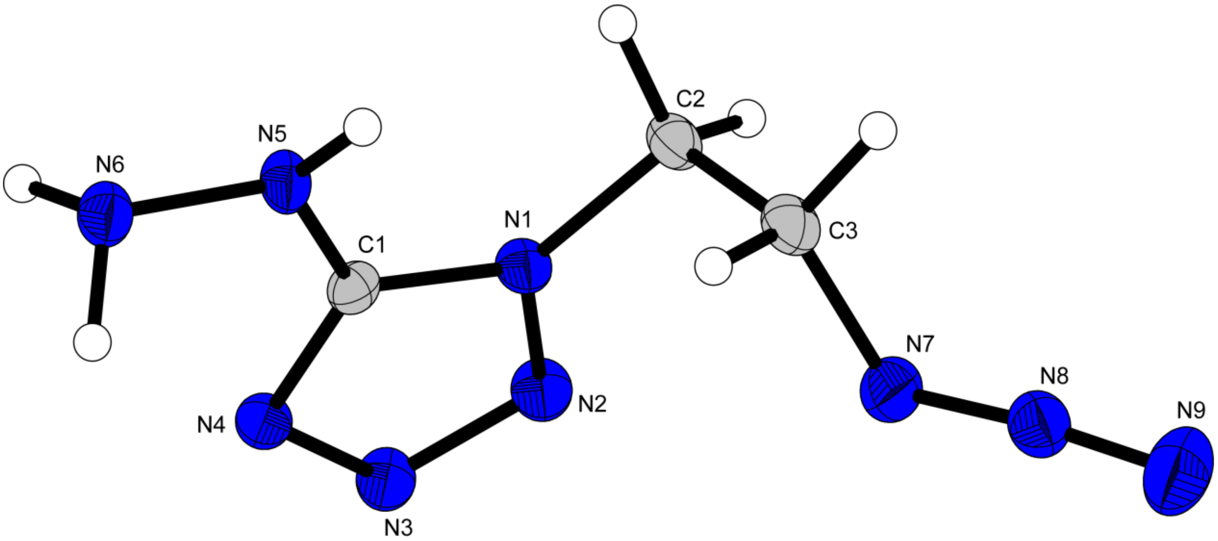

Figure S3. Representation of the molecular unit of **5**, showing the atom-labeling scheme. Thermal ellipsoids represent the 50% probability level and hydrogen atoms are shown as small spheres of arbitrary radius.

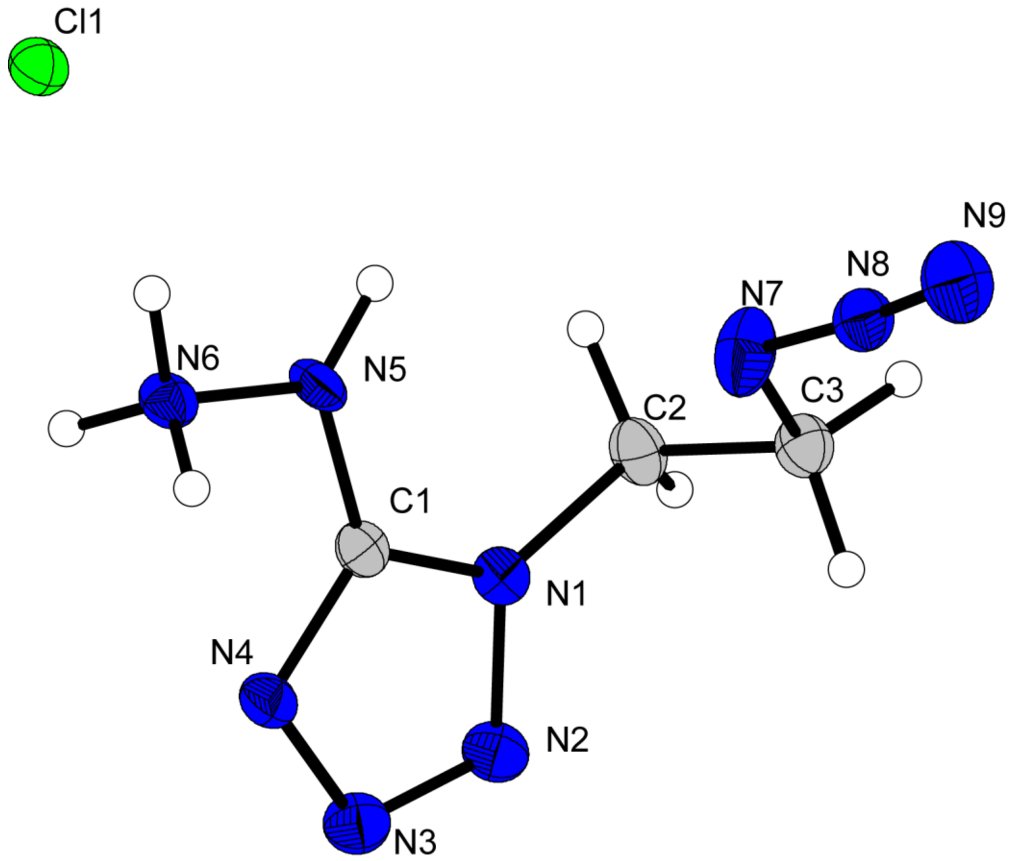

Figure S4. Representation of the molecular unit of **6**, showing the atom-labeling scheme. Thermal ellipsoids represent the 50% probability level and hydrogen atoms are shown as small spheres of arbitrary radius.

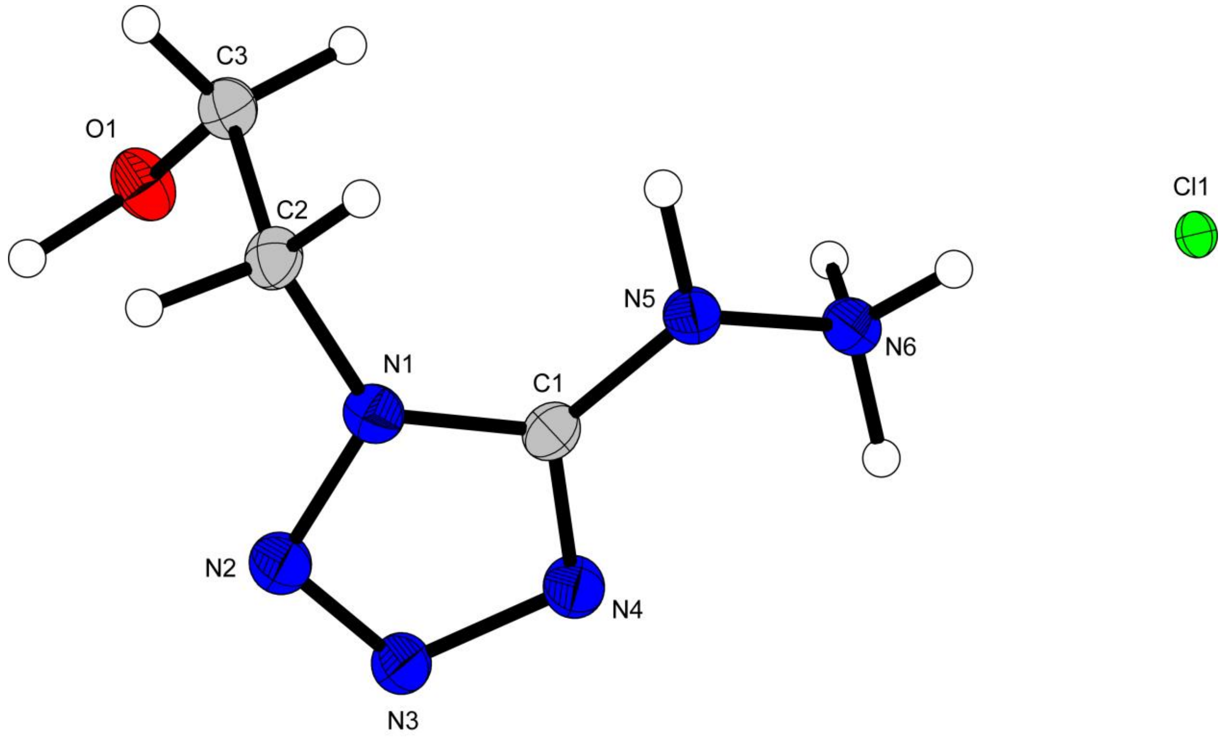

Figure S5. Representation of the molecular unit of **8** · HCl, showing the atom-labeling scheme. Thermal ellipsoids represent the 50% probability level and hydrogen atoms are shown as small spheres of arbitrary radius.

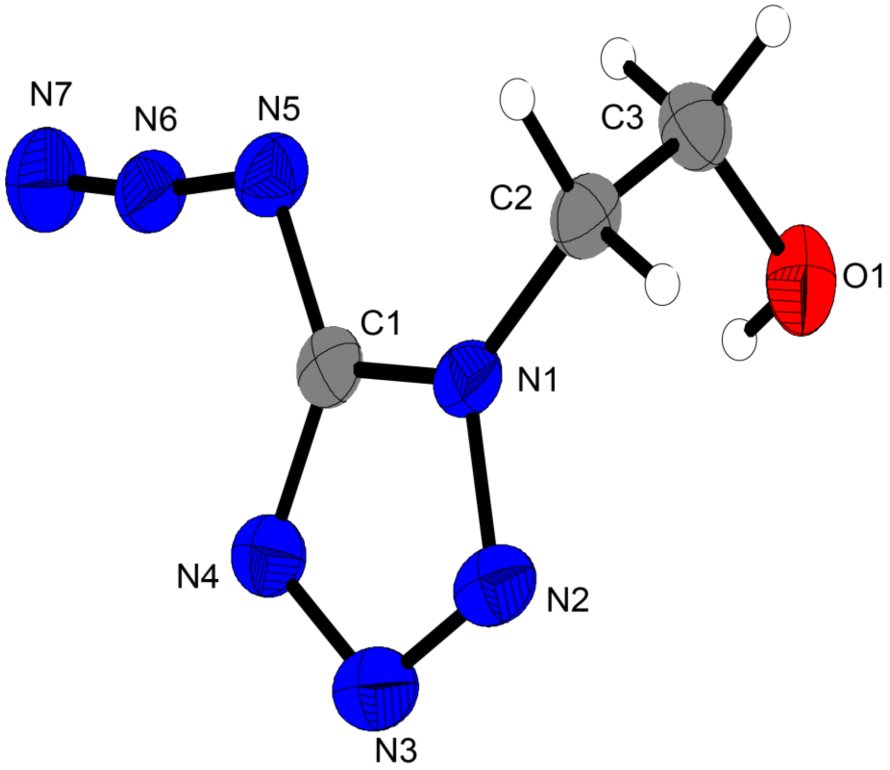

Figure S6. Representation of the molecular unit of **9**, showing the atom-labeling scheme. Thermal ellipsoids represent the 50% probability level and hydrogen atoms are shown as small spheres of arbitrary radius.

Table S1. Crystallographic data and structure refinement details for the prepared compounds **2a**, **2b** and **2d**.

	**2a**	**2b**	**2d**
Formula	$C_3H_4N_9O_2$, NH_4	$C_3H_4N_9O_2$, NH_3OH	$Ag_2C_6H_8N_{18}O_4$
FW [g mol^{-1}]	216.19	232.19	612.04
Crystal system	triclinic	triclinic	orthorhombic
Space group	*P*−1 (No. 2)	*P*−1 (No. 2)	*Pbca* (No. 61)
Color / Habit	colorless plate	colorless block	colorless platelet
Size [mm]	0.01 x 0.30 x 0.30	0.30 x 0.30 x 0.50	0.10 x 0.15 x 0.25
a [Å]	4.3043(3)	7.2416(6)	8.9776(6)
b [Å]	6.2716(5)	7.3297(8)	11.1520(12)
c [Å]	16.6686(13)	18.1018(18)	33.395(3)
α [°]	83.082(6)	89.358(9)	
β [°]	88.293(6)	79.717(8)	
γ [°]	84.288(6)	77.681(8)	
V [Å^3]	444.40(6)	923.26(16)	3343.5(5)
Z	2	4	8
$\rho_{calc.}$ [g cm^{-3}]	1.616	1.671	2.432
μ [mm^{-1}]	0.135	0.144	2.411
F(000)	224	480	2368
$\lambda_{MoK\alpha}$ [Å]	0.71073	0.71073	0.71073
T [K]	123	105	293
θ Min-Max [°]	2.5, 26.4	2.3, 26.4	2.4, 26.4
Dataset	-5: 5 ; -7: 7 ; -20: 20	-9: 9 ; -9: 7 ; -20: 22	-11: 11 ; -13: 13 ; -41: 41
Reflections collected	6814	7565	28293
Independent refl.	1812	3765	3406
R_{int}	0.035	0.032	0.166
Observed reflections	1427	2433	2254
Parameters	168	353	271
R_1 (obs)[a]	0.0363	0.0485	0.0650
wR_2 (all data)[b]	0.0869	0.1216	0.1368
S [c]	1.02	1.03	1.09
Resd. dens [e Å^{-3}]	-0.19, 0.21	-0.24, 0.30	-0.85, 1.58
Device type	Xcalibur Sapphire3	Xcalibur Sapphire3	Xcalibur Sapphire3
Solution	SIR-92	SIR-92	SIR-92
Refinement	SHELXL-2013	SHELXL-2013	SHELXL-2013
Absorption correction	multi-scan	multi-scan	multi-scan
CCDC	2157201	2157198	2157203

[a]$R_1 = \Sigma||F_0|-|F_c||/\Sigma|F_0|$; [b]$wR_2 = [\Sigma[w(F_0^2-F_c^2)^2]/\Sigma[w(F_0)^2]]^{1/2}$; $w = [\sigma c^2(F_0^2)+(xP)^2+yP]^{-1}$ and $P=(F_0^2+2F_c^2)/3$; [c]$S = (\Sigma[w(F_o^2-F_c^2)^2]/(n-p))^{1/2}$ (n = number of reflections; p = total number of parameters).

Table S2. Crystallographic data and structure refinement details for the prepared compounds **5**-**7**.

	**5**	**6**	**7**
Formula	$C_3H_7N_9$	$C_3H_8N_9Cl$	$C_3H_4N_{10}$
FW [g mol^{-1}]	169.18	205.63	180.16
Crystal system	monoclinic	monoclinic	orthorhombic
Space group	$P2_1/c$ (No. 14)	P21/c (No. 14)	*Fdd*2 (No. 43)
Color / Habit	colorless block	colourless block	colorless block
Size [mm]	0.50 x 0.50 x 0.50	0.10 x 0.50 x 0.50	0.05 x 0.25 x 0.50
a [Å]	6.0070(5)	12.7917(11)	25.526(2)
b [Å]	10.9792(10)	7.7802(6)	12.6485(10)
c [Å]	10.9160(11)	8.8236(6)	9.4801(9)
α [°]	90	90	
β [°]	95.467(7)	101.731(6)	
γ [°]	90	90	
V [Å^3]	716.66(11)	859.80(12)	3060.8(4)
Z	4	4	16
$\rho_{calc.}$ [g cm^{-3}]	1.568	1.589	1.564
μ [mm^{-1}]	0.120	0.416	0.122
F(000)	352	424	1472
$\lambda_{MoK\alpha}$ [Å]	0.71073	0.71073	0.71073
T [K]	101	101	102
θ Min-Max [°]	2.6, 29.2	3.1, 26.4	2.8, 26.4
Dataset	-7: 7 ; -13: 5 ; -14: 12	-12: 15 ; -7: 9 ; -10: 11	-31: 31 ; -15: 15 ; -11: 11
Reflections collected	3304	6302	11967
Independent refl.	1627	1755	1568
R_{int}	0.022	0.042	0.062
Observed reflections	1285	1369	1343
Parameters	137	119	135
R_1 (obs)[a]	0.0420	0.0438	0.0366
wR_2 (all data)[b]	0.0985	0.1159	0.0758
S [c]	1.04	1.06	1.05
Resd. dens [e Å^{-3}]	-0.19, 0.24	-0.67, 0.62	-0.12, 0.17
Device type	Xcalibur Sapphire3	Xcalibur Sapphire3	Xcalibur Sapphire3
Solution	SIR-92	SIR-92	SIR-92
Refinement	SHELXL-2013	SHELXL-2013	SHELXL-2013
Absorption correction	multi-scan	multi-scan	multi-scan
CCDC	2157204	2157196	2157199

[a] $R_1 = \Sigma||F_0|-|F_c||/\Sigma|F_0|$; [b] $wR_2 = [\Sigma[w(F_0^2-F_c^2)^2]/\Sigma[w(F_0)^2]]^{1/2}$; $w = [\sigma c^2(F_0^2)+(xP)^2+yP]^{-1}$ and $P=(F_0^2+2F_c^2)/3$; [c] $S = (\Sigma[w(F_o^2-F_c^2)^2]/(n-p))^{1/2}$ (n = number of reflections; p = total number of parameters).

Table S3. Crystallographic data and structure refinement details for the prepared compounds **8** · HCl-**10**.

	**8 · HCl**	**9**	**10**
Formula	$C_3H_9N_6OCl$	$C_3H_5N_7O$	$C_3H_4N_8O_3$
FW [g mol^{-1}]	180.61	155.14	200.14
Crystal system	monoclinic	monoclinic	monoclinic
Space group	$P2_1/c$ (No. 14)	$P2_1/n$ (No. 14)	$P2_1/n$ (No. 14)
Color / Habit	colorless block	colorless block	colorless rod
Size [mm]	0.20 x 0.50 x 0.50	0.08 x 0.12 x 0.15	0.10 x 0.20 x 0.50
a [Å]	10.738(3)	8.3335(6)	9.5705(13)
b [Å]	6.5212(11)	8.3390(6)	6.7210(7)
c [Å]	11.244(2)	9.5255(7)	12.3677(16)
α [°]	90	90	90
β [°]	100.26(2)	98.792(7)	97.633(12)
γ [°]	90	90	90
V [Å^3]	774.8(3)	654.18(8)	788.48(17)
Z	4	4	4
$\rho_{calc.}$ [g cm^{-3}]	1.548	1.575	1.686
μ [mm^{-1}]	0.448	0.127	0.148
F(000)	376	320	408
$\lambda_{MoK\alpha}$ [Å]	0.71073	0.71073	0.71073
T [K]	104	200	101
θ Min-Max [°]	3.6, 26.4	4.3, 26.0	2.5, 25.7
Dataset	-13: 12 ; -8: 8 ; -14: 14	-10: 10 ; -10: 10 ; -11: 11	-9: 11 ; -8: 8 ; -15: 13
Reflections collected	5599	3070	5590
Independent refl.	1577	1284	1494
R_{int}	0.039	0.022	0.033
Observed reflections	1309	924	1215
Parameters	136	120	143
R_1 (obs)[a]	0.0492	0.0302	0.0595
wR_2 (all data)[b]	0.1418	0.0691	0.1736
S [c]	1.09	0.91	1.12
Resd. dens [e Å^{-3}]	-0.42, 0.91	-0.18, 0.15	-0.37, 0.60
Device type	Xcalibur Sapphire3	Xcalibur Sapphire3	Xcalibur Sapphire3
Solution	SIR-92	SIR-92	SIR-92
Refinement	SHELXL-2013	SHELXL-2013	SHELXL-2013
Absorption correction	multi-scan	multi-scan	multi-scan
CCDC	2157197	2157202	2157200

[a]$R_1 = \Sigma||F_0|-|F_c||/\Sigma|F_0|$; [b]$wR_2 = [\Sigma[w(F_0^2-F_c^2)^2]/\Sigma[w(F_0)^2]]^{1/2}$; $w = [\sigma c^2(F_0^2)+(xP)^2+yP]^{-1}$ and $P=(F_0^2+2F_c^2)/3$; [c]$S = (\Sigma[w(F_o^2-F_c^2)^2]/(n-p))^{1/2}$ (n = number of reflections; p = total number of parameters).

Table S4. Crystallographic data and structure refinement details for the prepared compounds **11** and **12**.

	**11**	**12**
Formula	$C_2H_3N_7$	$C_2H_3N_7$
FW [g mol^{-1}]	125.11	125.11
Crystal system	monoclinic	monoclinic
Space group	$P2_1/m$ (No. 11)	$P2_1/c$ (No. 14)
Color / Habit	colorless needle	colorless block
Size [mm]	0.06 x 0.10 x 0.26	0.10 x 0.12 x 0.13
a [Å]	8.7382(4)	6.6219(5)
b [Å]	6.2408(4)	8.7588(7)
c [Å]	10.2986(6)	9.6392(8)
α [°]	90	90
β [°]	90.722(4)	108.858(7)
γ [°]	90	90
V [Å^3]	561.57(6)	529.06(8)
Z	4	4
$\rho_{calc.}$ [g cm^{-3}]	1.480	1.571
μ [mm^{-1}]	0.115	0.123
F(000)	256	256
$\lambda_{MoK\alpha}$ [Å]	0.71073	0.71073
T [K]	200	173
θ Min-Max [°]	3.8, 26.0	4.0, 26.0
Dataset	-10: 8 ; -7: 7 ; -12: 8	-5: 8 ; -10: 8 ; -10: 11
Reflections collected	2919	2662
Independent refl.	1206	1037
R_{int}	0.037	0.036
Observed reflections	729	674
Parameters	112	94
R_1 (obs)[a]	0.0320	0.0331
wR_2 (all data)[b]	0.0926	0.0854
S [c]	0.97	0.90
Resd. dens [e Å^{-3}]	-0.18, 0.16	-0.16, 0.18
Device type	Xcalibur Sapphire3	Xcalibur Sapphire3
Solution	SIR-92	SIR-92
Refinement	SHELXL-2013	SHELXL-2013
Absorption correction	multi-scan	multi-scan
CCDC	707542	707543

[a]$R_1 = \Sigma||F_0|-|F_c||/\Sigma|F_0|$; [b]$wR_2 = [\Sigma[w(F_0^2-F_c^2)^2]/\Sigma[w(F_0)^2]]^{1/2}$; $w = [\sigma c^2(F_0^2)+(xP)^2+yP]^{-1}$ and $P=(F_0^2+2F_c^2)/3$; [c]$S = (\Sigma[w(F_o^2-F_c^2)^2]/(n-p))^{1/2}$ (n = number of reflections; p = total number of parameters).

4. Computation

Heat of Formation Computation

All quantum chemical calculations were carried out using the Gaussian G09 program package.[S10] The enthalpies (H) and free energies (G) were calculated using the complete basis set (CBS) method of Petersson and co-workers in order to obtain very accurate energies. The CBS models are using the known asymptotic convergence of pair natural orbital expressions to extrapolate from calculations using a finite basis set to the estimated CBS limit. CBS-4 starts with an HF/3-21G(d) geometry optimization; the zero-point energy is computed at the same level. It then uses a large basis set SCF calculation as a base energy, and an MP2/6- 31+G calculation with a CBS extrapolation to correct the energy through second order. A MP4(SDQ)/6-31+ (d,p) calculation is used to approximate higher order contributions. In this study, we applied the modified CBS-4M.

Heats of formation of the synthesized ionic compounds were calculated using the atomization method (equation E1) using room temperature CBS-4M enthalpies, which are summarized in Table S5.[S11, S12]

$$\Delta_f H^\circ_{(g,\ M,\ 298)} = H_{(Molecule,\ 298)} - \sum H^\circ_{(Atoms,\ 298)} + \sum \Delta_f H^\circ_{(Atoms,\ 298)} \qquad (E1)$$

Table S5. CBS-4M electronic enthalpies for atoms C, H, N and O and their literature values for atomic $\Delta H^\circ{}_f{}^{298}$ / kJ mol^{-1}

	$-H^{298}$ [a.u.]	NIST[S13]
H	0.500991	218.2
C	37.786156	717.2
N	54.522462	473.1
O	74.991202	249.5

For neutral compounds the sublimation enthalpy, which is needed to convert the gas phase enthalpy of formation to the solid state one, was calculated by the *Trouton* rule.[S14] For ionic compounds, the lattice energy (U_L) and lattice enthalpy (ΔH_L) were calculated from the corresponding X-ray molecular volumes according to the equations provided by *Jenkins* and *Glasser*.[S15] With the calculated lattice enthalpy the gas-phase enthalpy of formation was converted into the solid state (standard conditions)

enthalpy of formation. These molar standard enthalpies of formation (ΔH_m) were used to calculate the molar solid state energies of formation (ΔU_m) according to equation E2.

$$\Delta U_m = \Delta H_m - \Delta n\ RT \qquad \text{(E2)}$$

(Δn being the change of moles of gaseous components)

The calculation results are summarized in Table S6.

Table S6. Calculation results.

	$-H^{298}$ [a] [a.u.]	$\Delta_f H°$(g,M) [kJ mol^{-1}] [b]	V_M [Å^3] [c]	ΔU_L; ΔH_L [d] [kJ mol^{-1}]	$\Delta_f H°$(s) [e] [kJ mol^{-1}]	Δn [f]	$\Delta_f U$(s) [g] [kJ mol^{-1}]
**A^- (2)**	758.815212	506.6					
**NH_4^+**	56.796608	635.3					
**NH_3OH^+**	112.630523	773.4					
**2**	759.333150	680.1	-	-	647.1	−8.0	666.9
**2a**	-	-	228.0	487.8; 492.8	649.1	−10.0	673.9
**2b**	-	-	238.0	482.6; 487.6	705.5	−10.5	731.5
**7**	663.165046	980.3	-	-	932.7	−7.0	950.1
**10**	779.173027	574.3	-	-	514.5	−7.5	533.1
**11**	460.540365	649.6	-	-	594.5	−5.0	606.9
**12**	460.547855	630.0	-	-	566.9	−5.0	579.3

[a] CBS-4M electronic enthalpy; [b] gas phase enthalpy of formation; [c] molecular volumes taken from X-ray structures and corrected to room temperature; [d] lattice energy and enthalpy (calculated using Jenkins and Glasser equations); [e] standard solid state enthalpy of formation; [f] Δn being the change of moles of gaseous components when formed; [g] solid state energy of formation.

5. NMR Spectroscopy

^{1}H-NMR (400 MHz, DMSO-D$_6$, ppm)

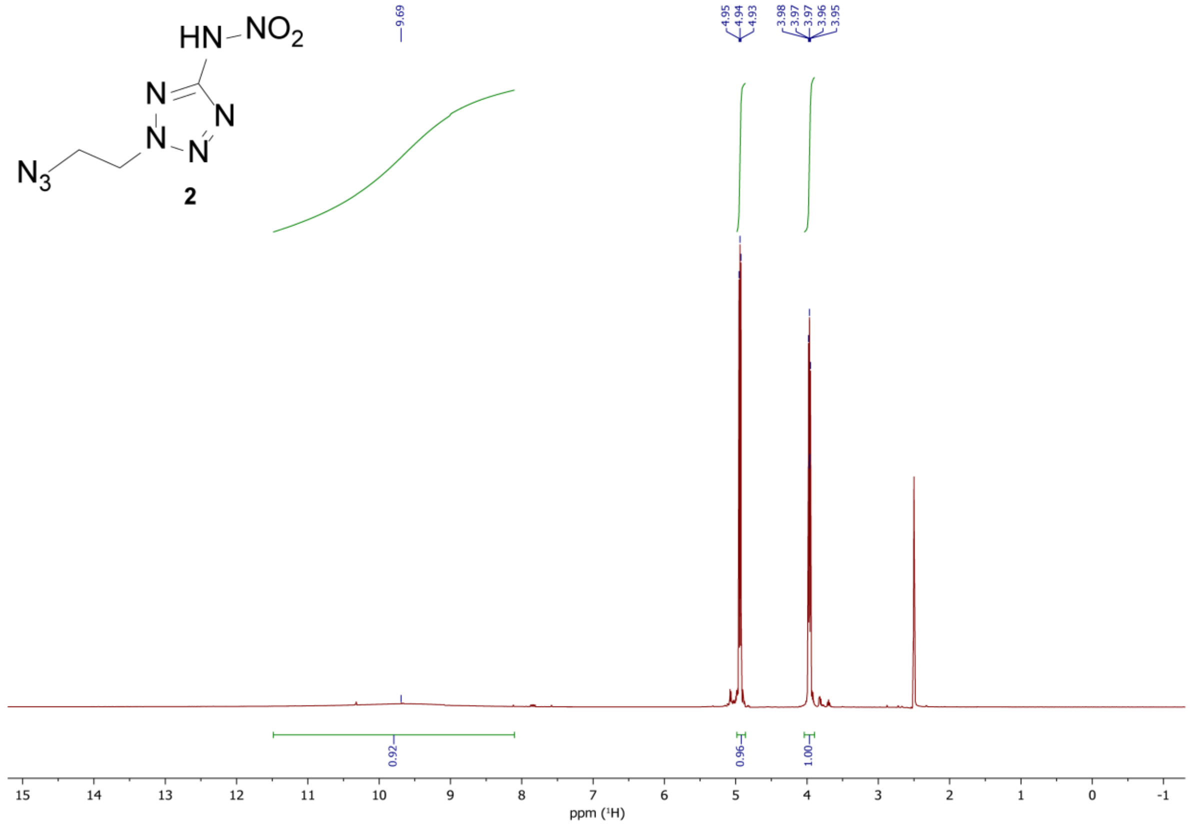

^{13}C-NMR (101 MHz, DMSO-D$_6$, ppm)

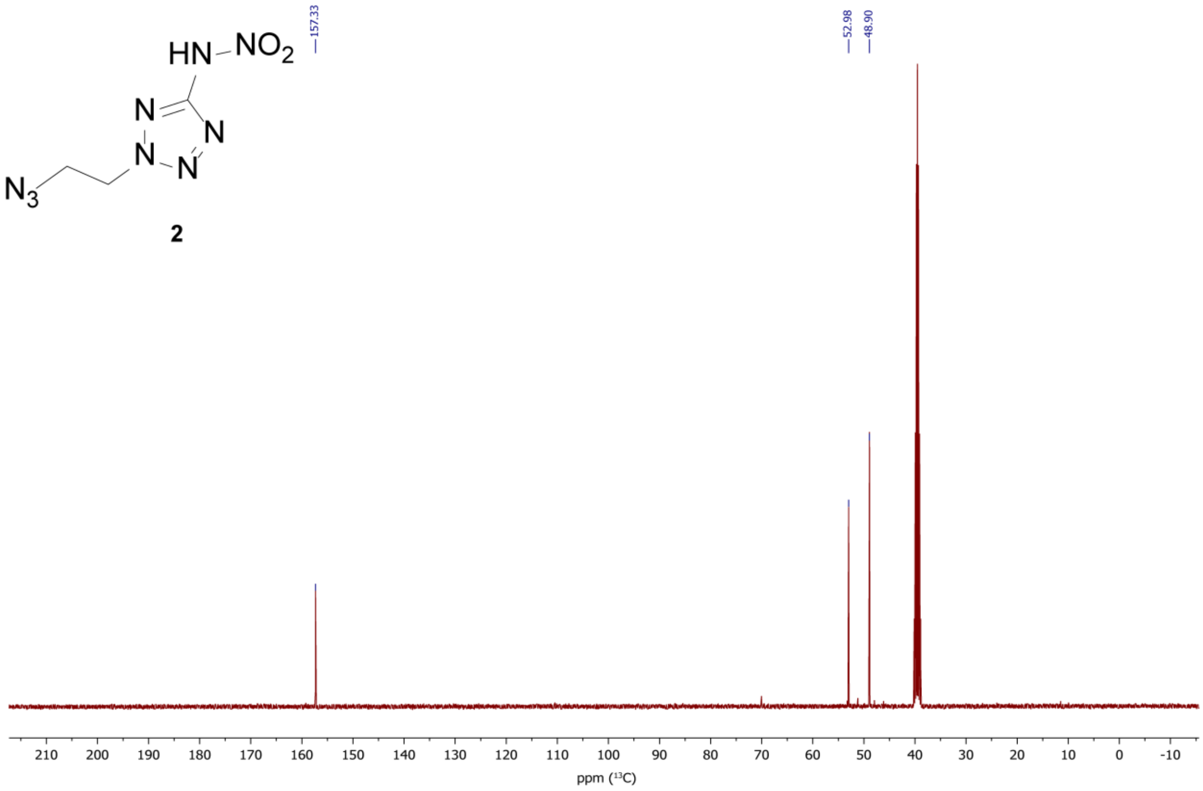

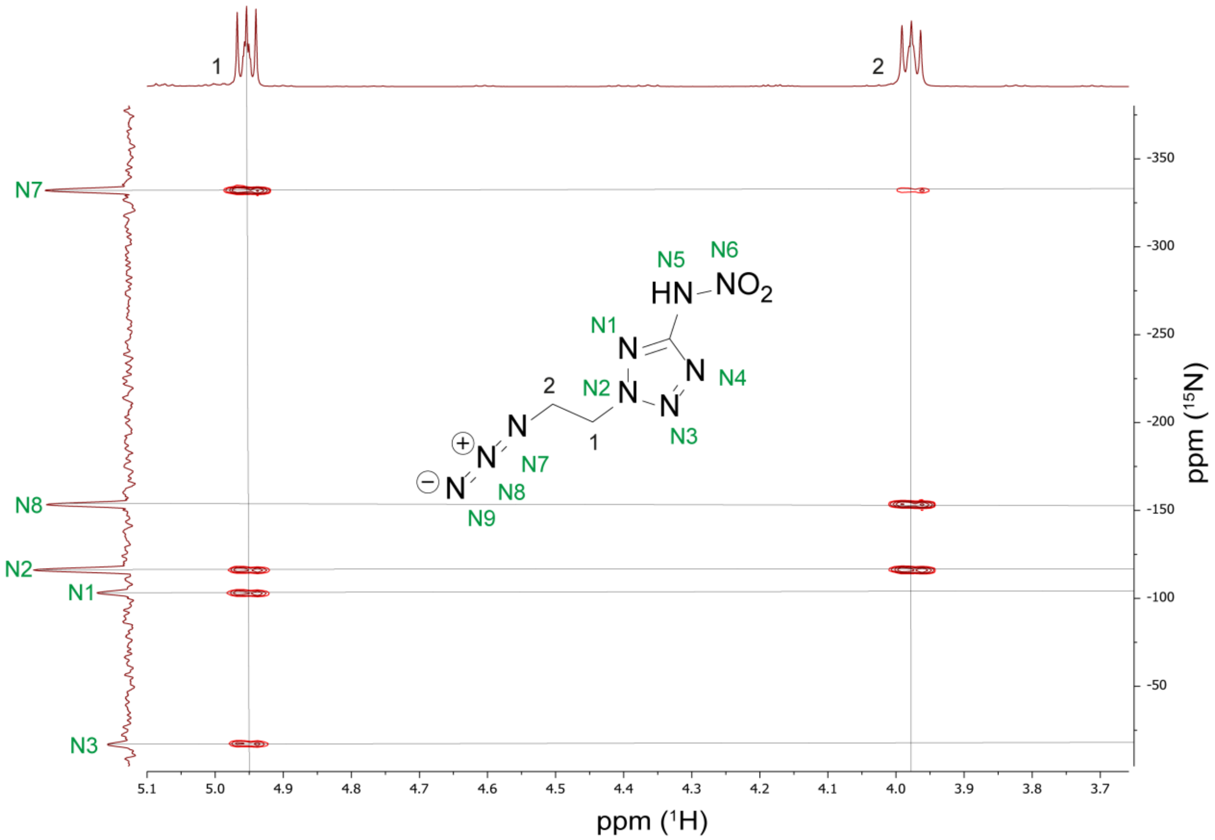

1
2
N7
N8
N2
N1
N3
N5
N6
HN
NO2
N1
N4
N2
N3
N7
N8
N9
-350
-300
-250
-200
-150
-100
-50
ppm (15N)
5.1
5.0
4.9
4.8
4.7
4.6
4.5
4.4
4.3
4.2
4.1
4.0
3.9
3.8
3.7
ppm (1H)

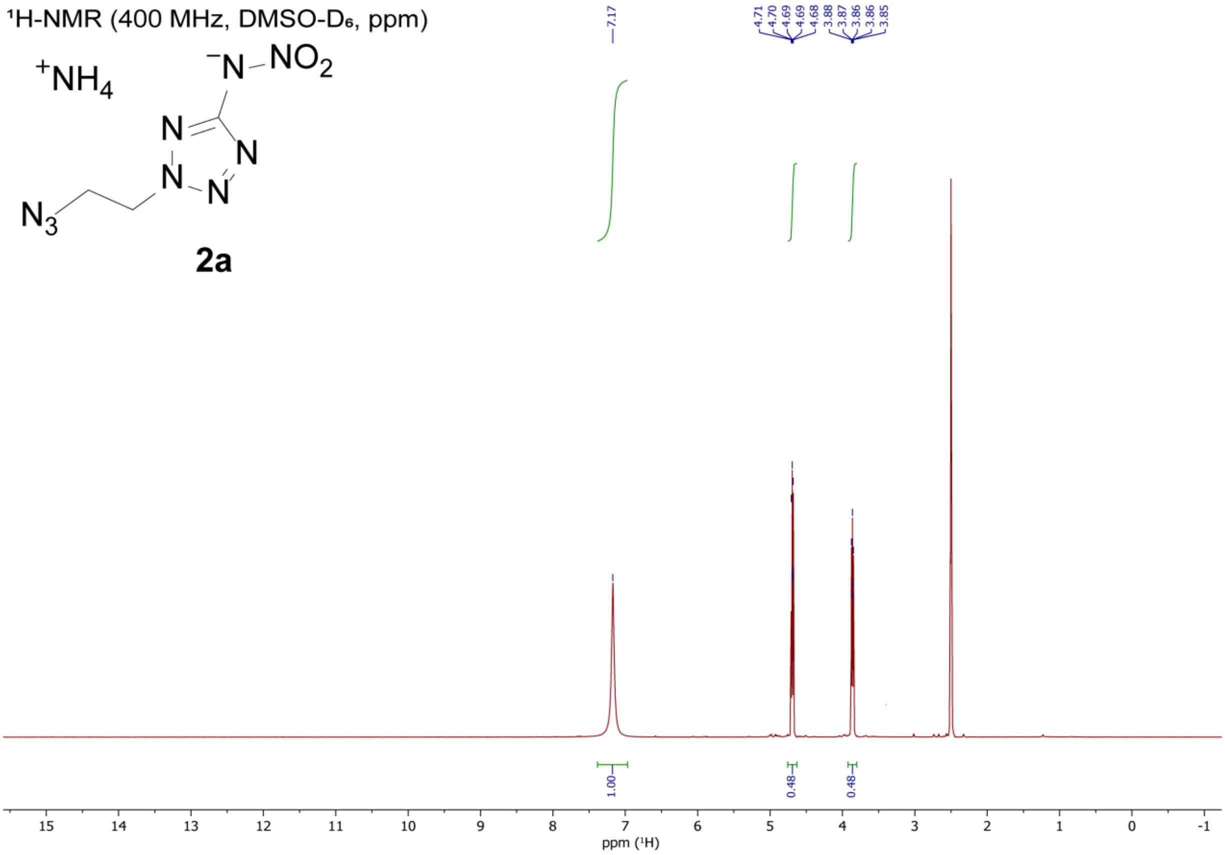

1H-NMR (400 MHz, DMSO-D6, ppm)
+NH4
–N
NO2
N3
2a
7.17
4.71
4.70
4.69
4.69
4.68
3.88
3.87
3.86
3.86
3.85
1.00
0.48
0.48
15
14
13
12
11
10
9
8
7
6
5
4
3
2
1
0
-1
ppm (1H)

^{13}C-NMR (101 MHz, DMSO-D$_6$, ppm)

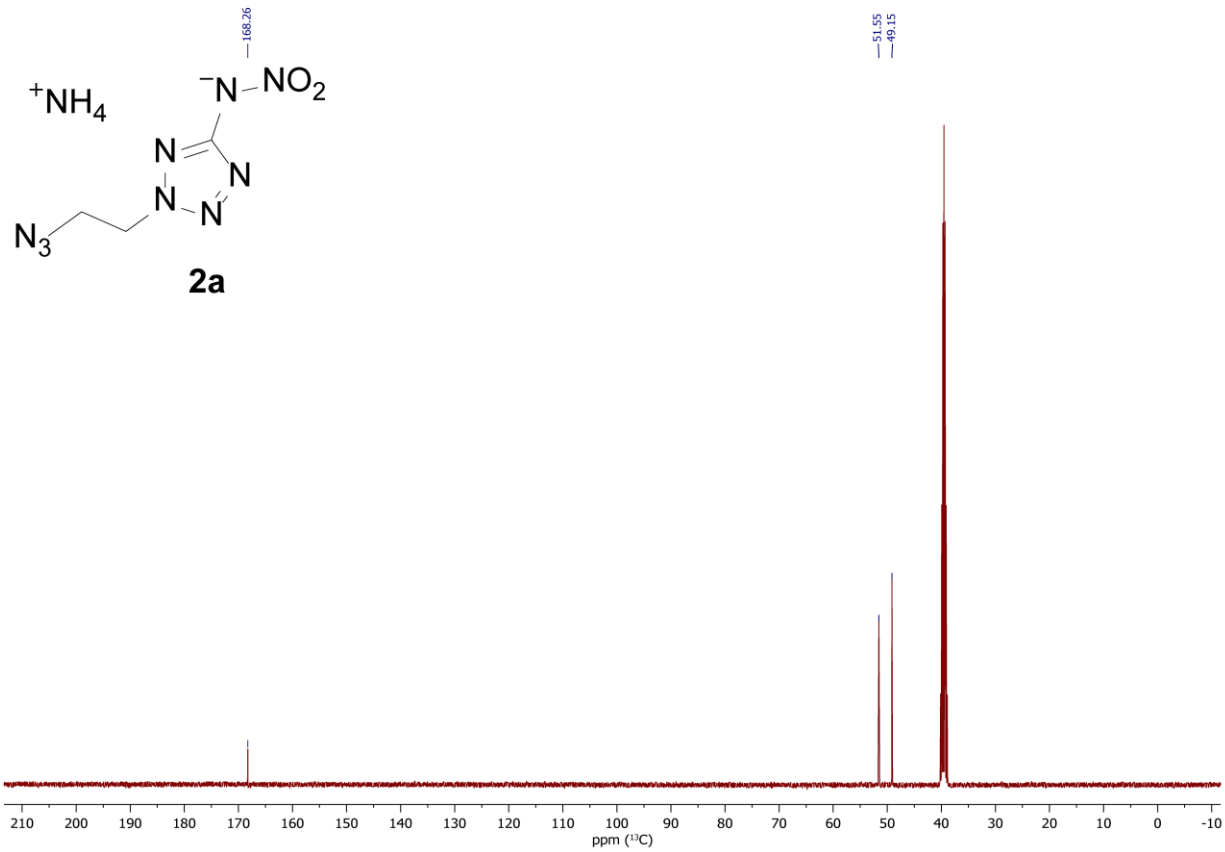

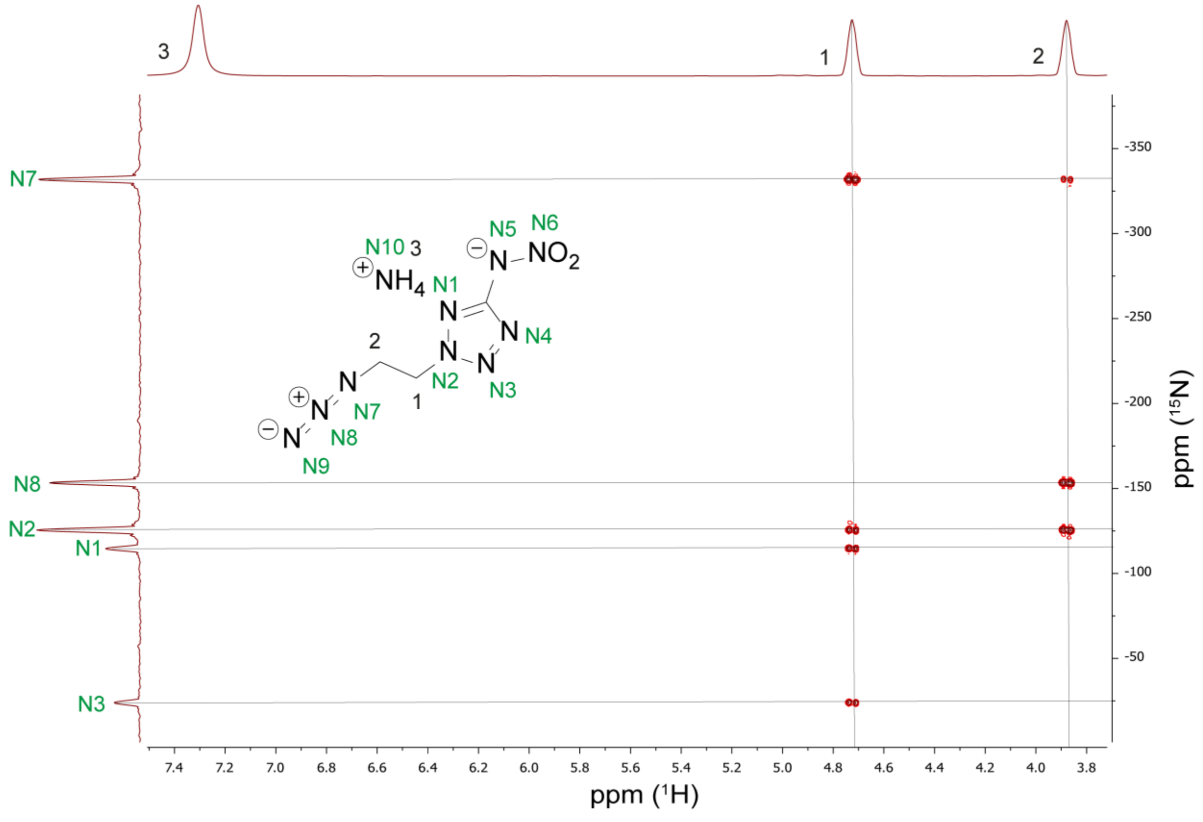

^{1}H-NMR (400 MHz, DMSO-D$_6$, ppm)

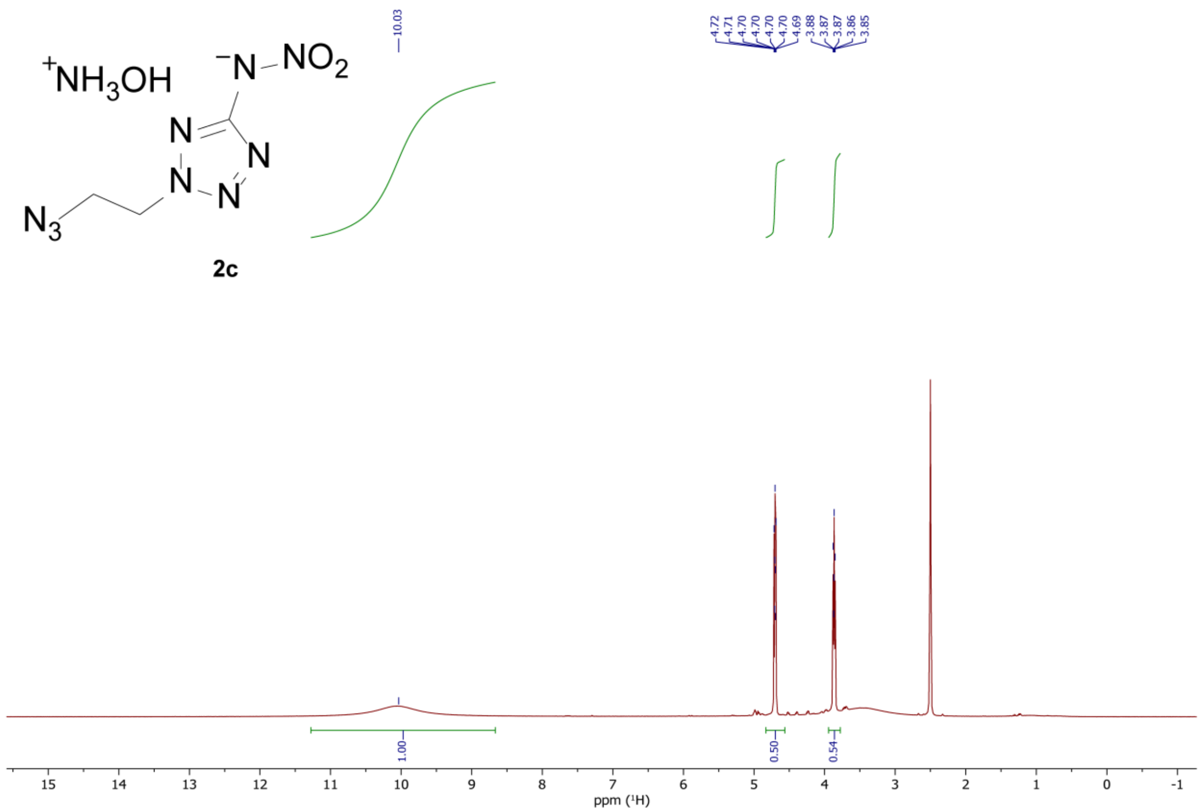

^{13}C-NMR (101 MHz, DMSO-D$_6$, ppm)

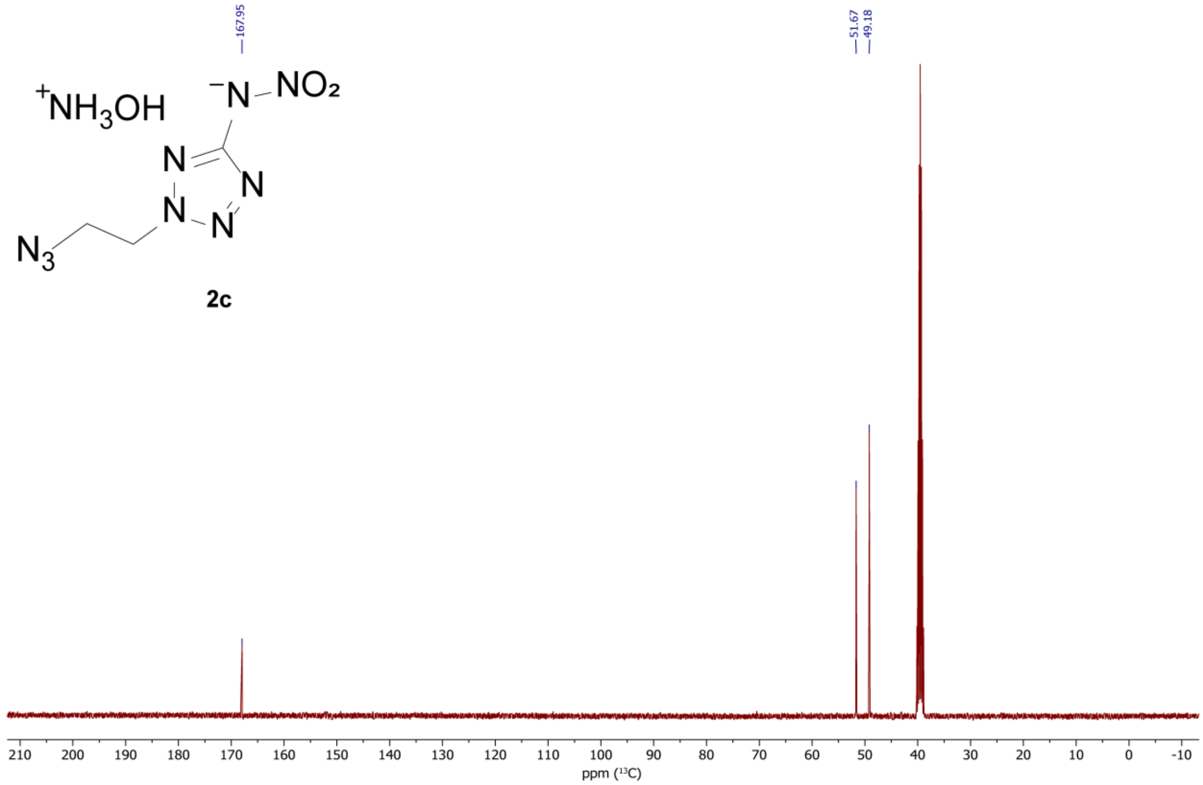

^{1}H-NMR (400 MHz, DMSO-D$_6$, ppm)

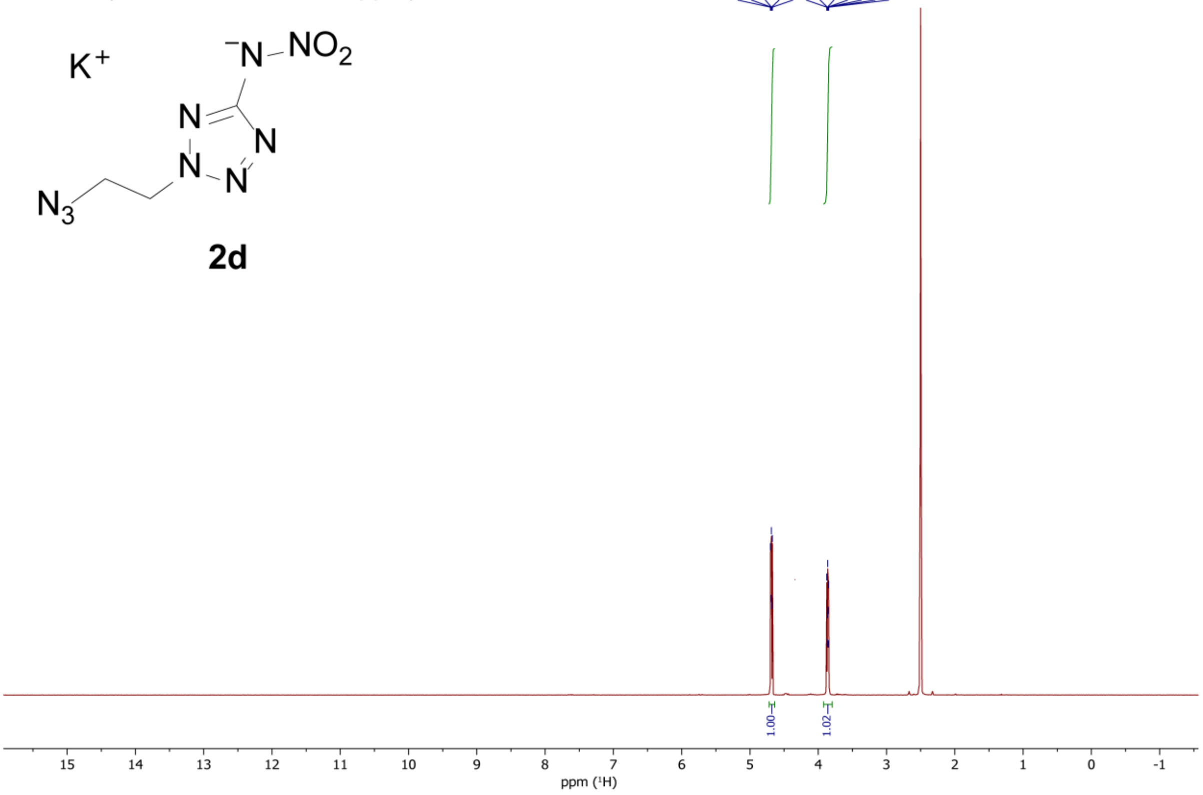

^{13}C-NMR (101 MHz, DMSO-D$_6$, ppm)

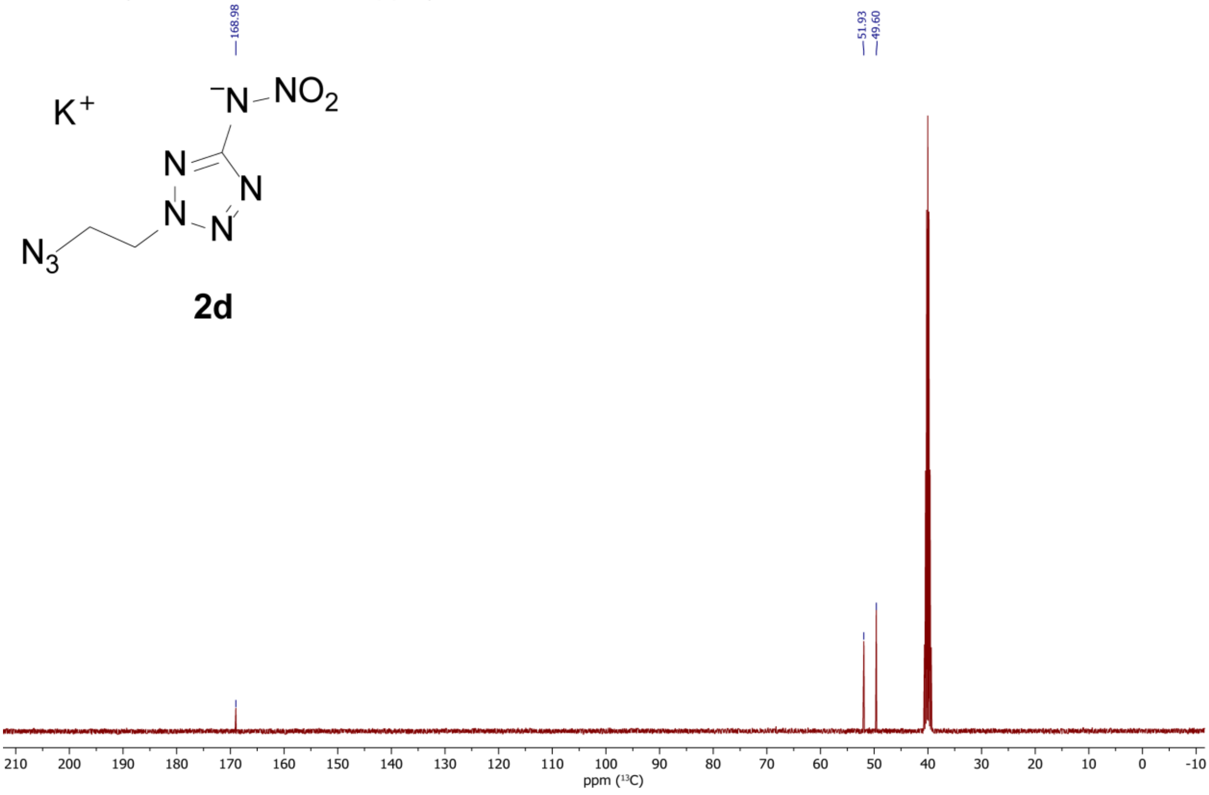

^{1}H-NMR (400 MHz, DMSO-D_6, ppm)

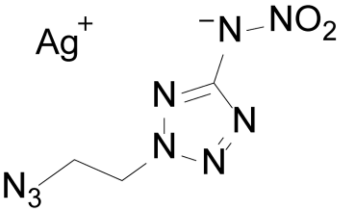

2e

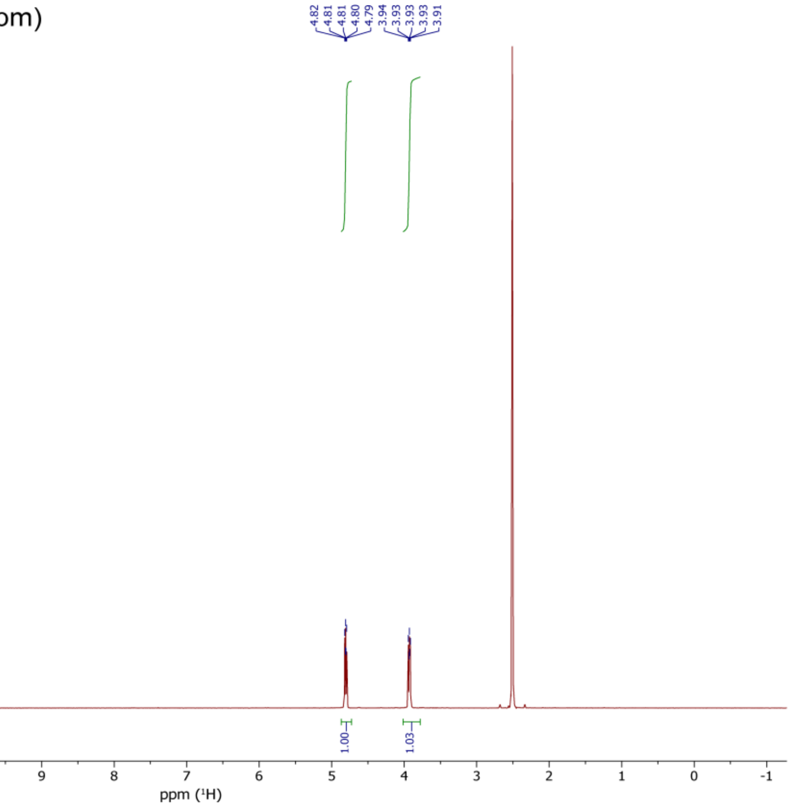

^{13}C-NMR (101 MHz, DMSO-D_6, ppm)

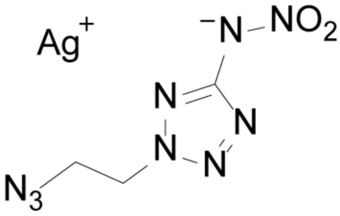

2e

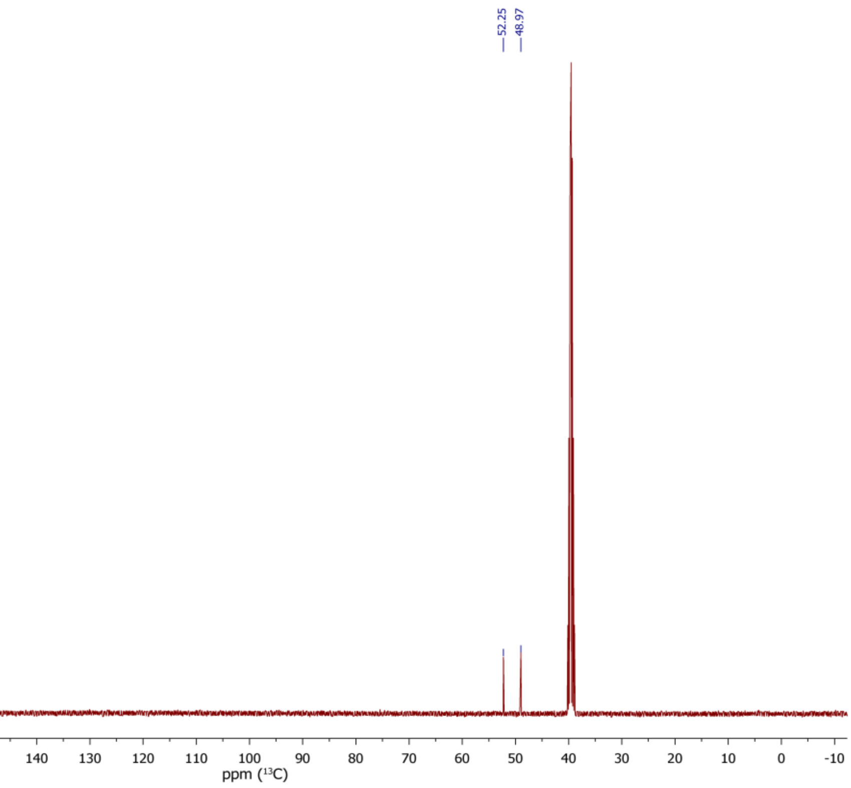

^{1}H NMR (400 MHz, DMSO-D₆, ppm)

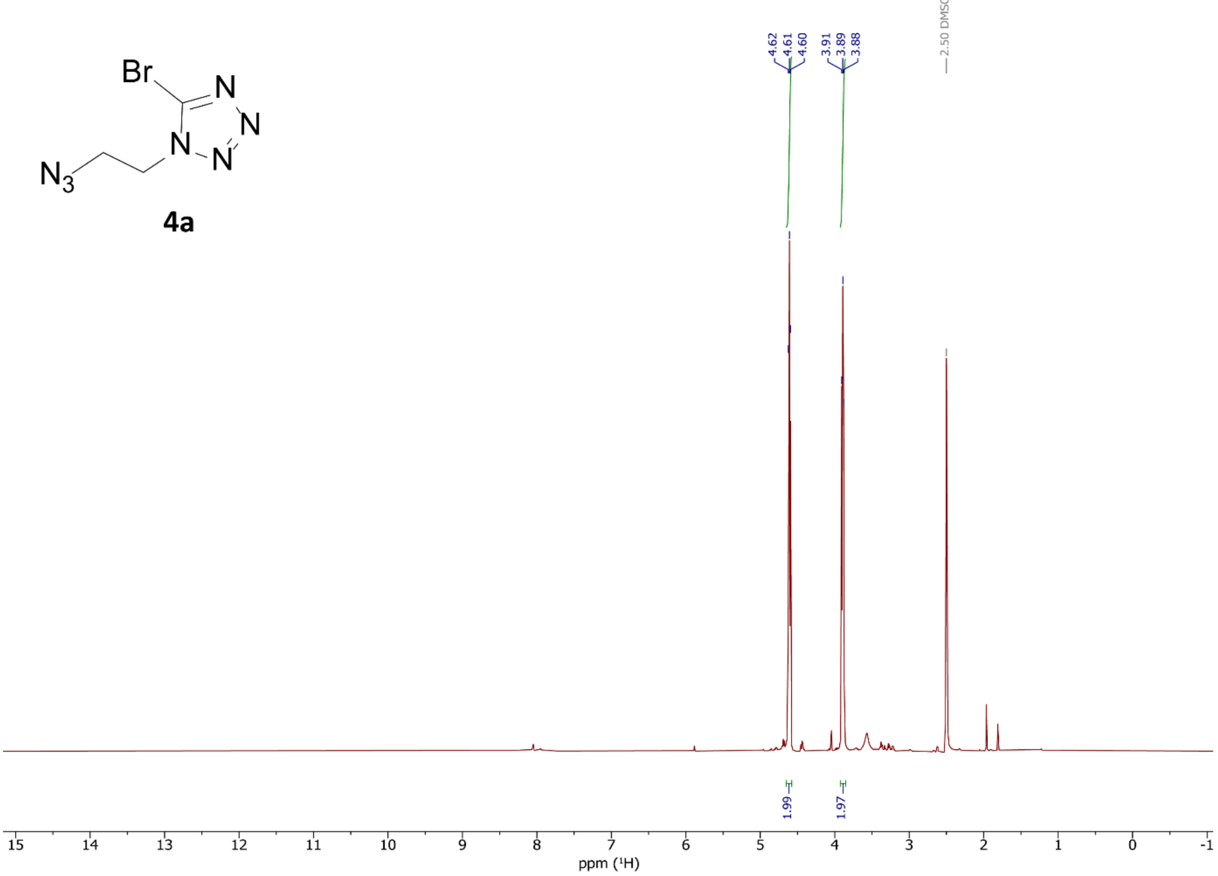

^{13}C NMR (101 MHz, DMSO-D₆, ppm)

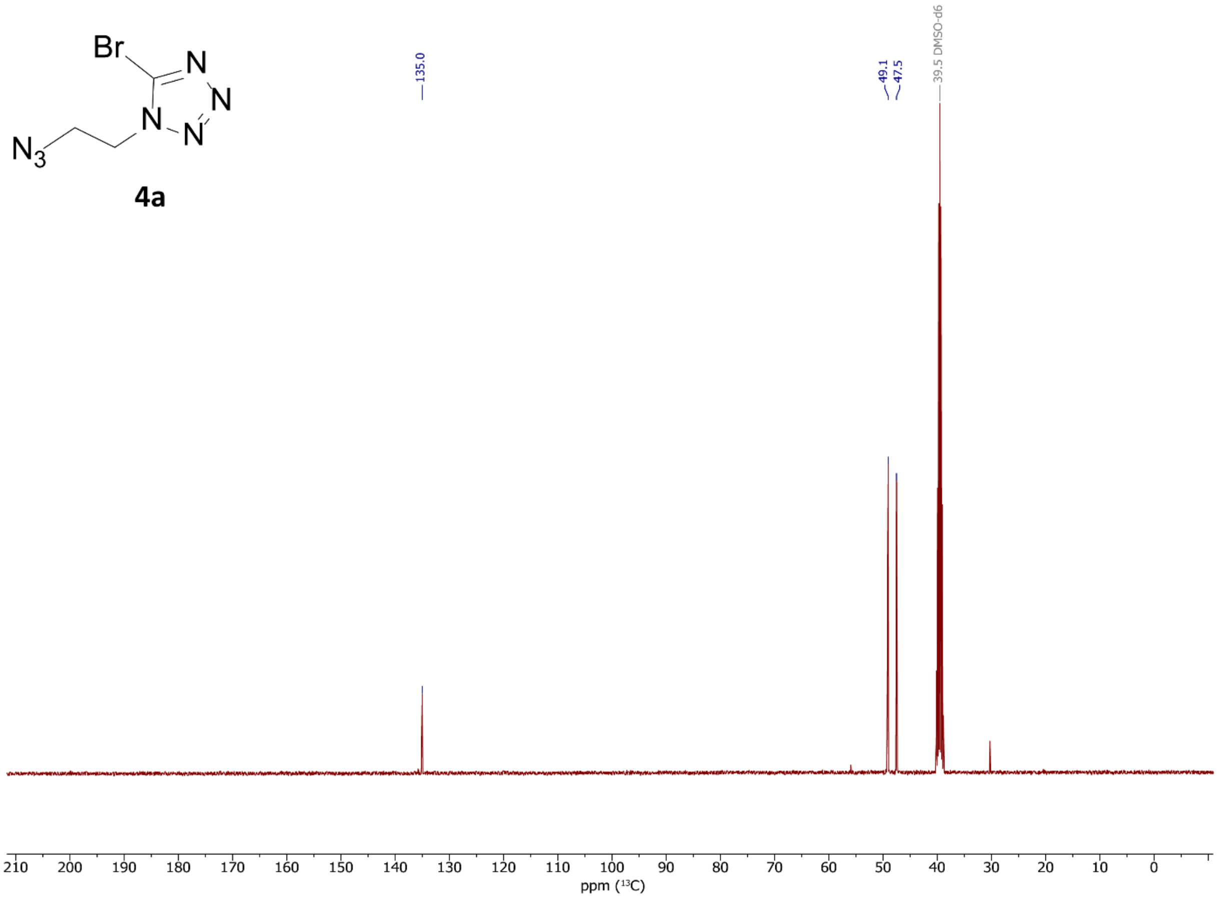

^{1}H NMR (400 MHz, DMSO-D$_6$, ppm)

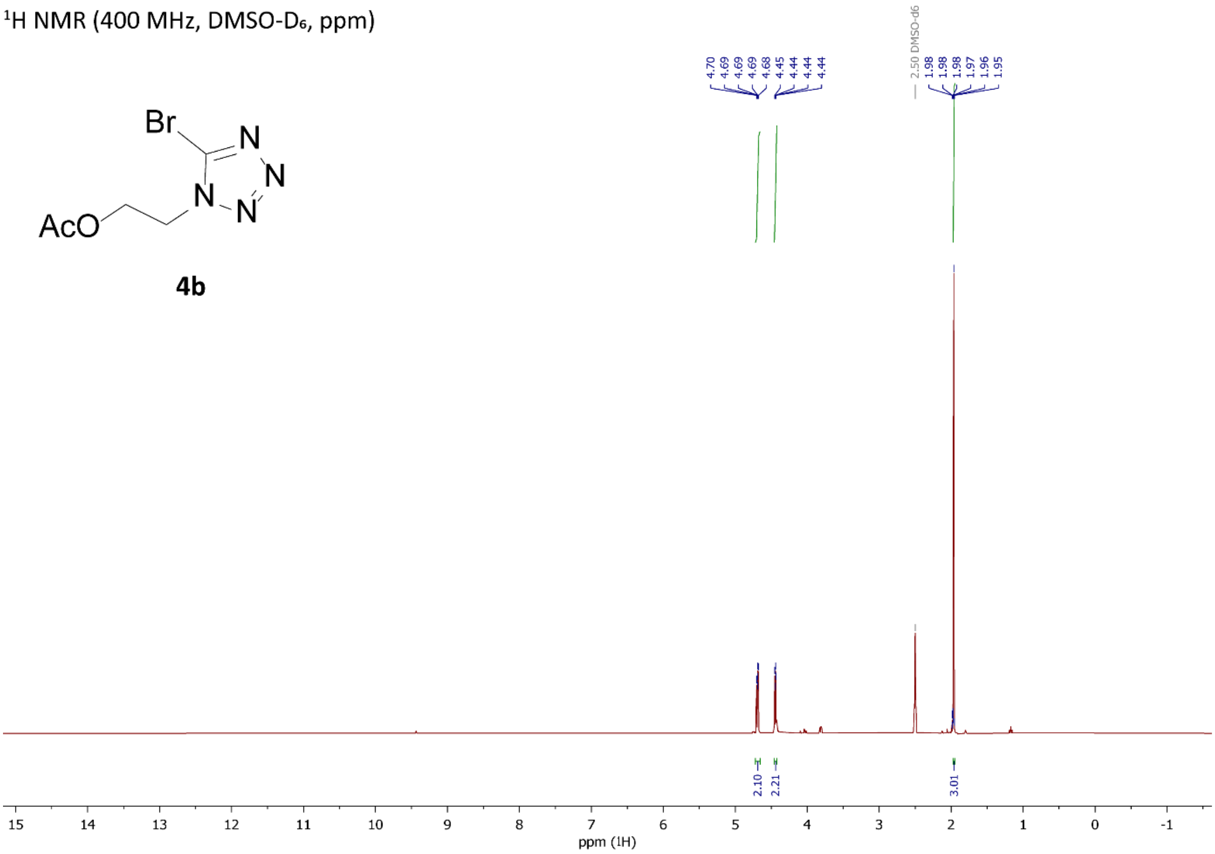

^{13}C NMR (101 MHz, DMSO-D$_6$, ppm)

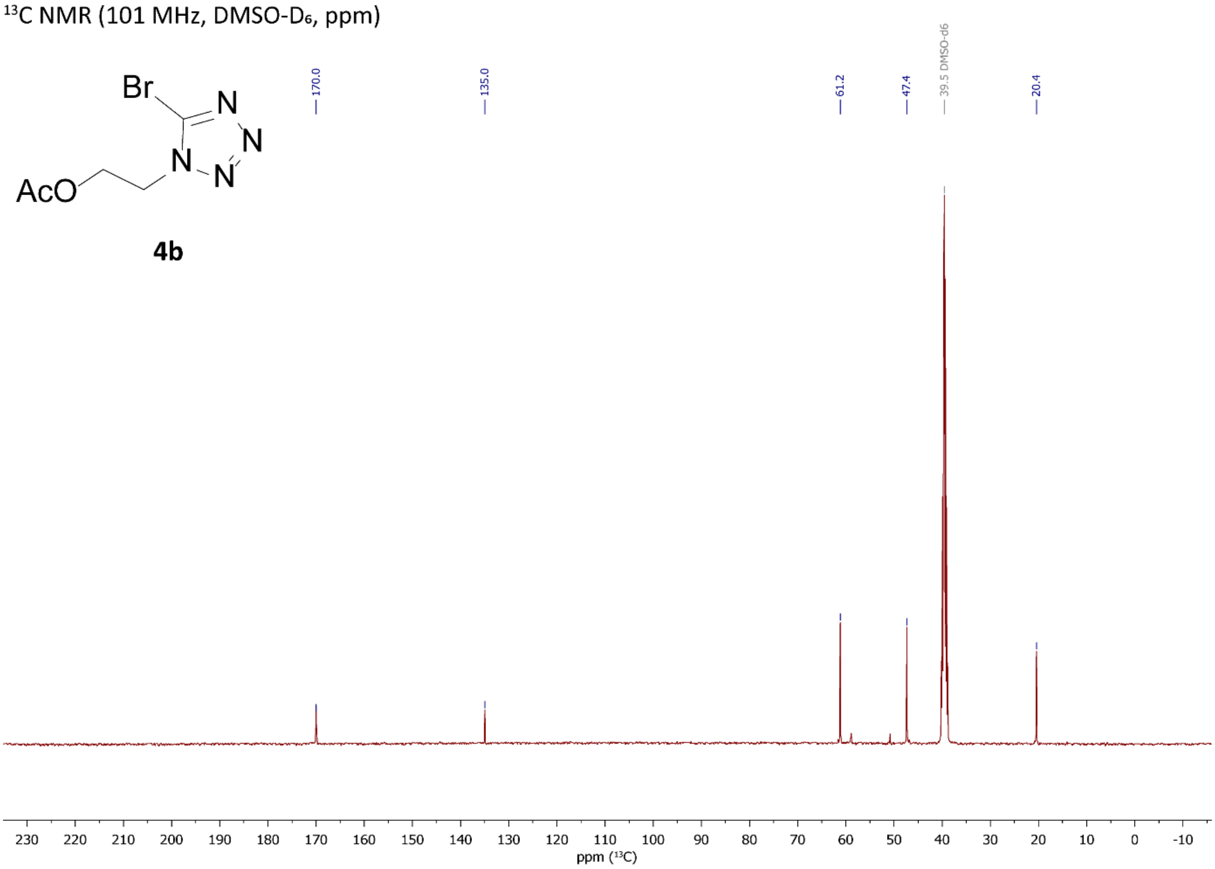

^1H NMR (400 MHz, DMSO-D₆, ppm)

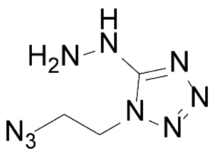

5

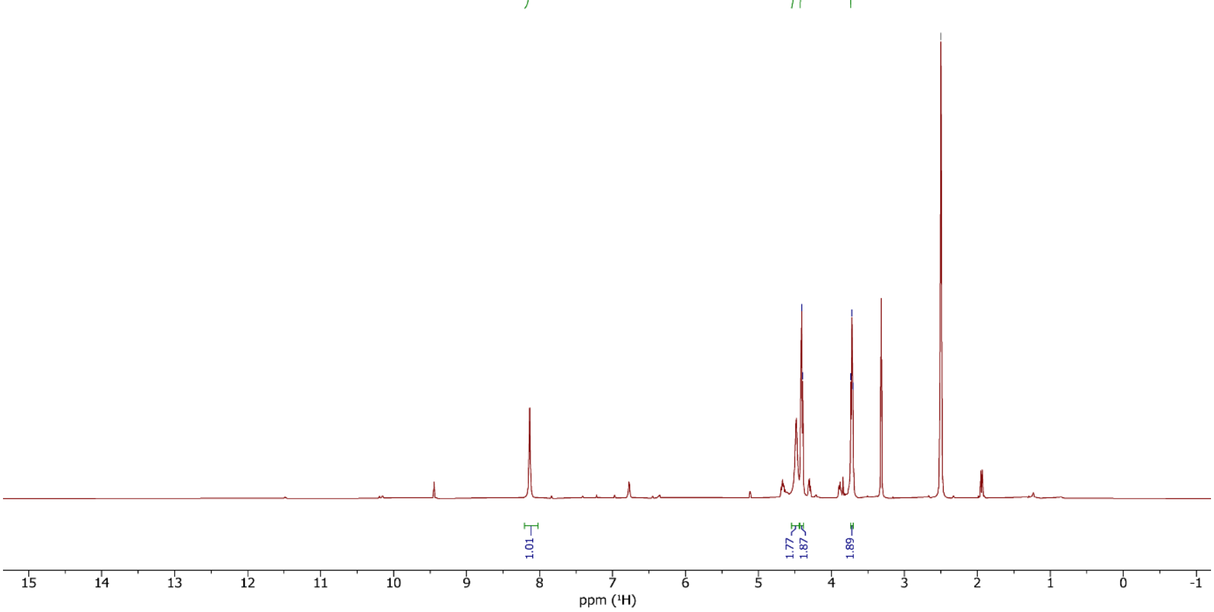

^{13}C NMR (101 MHz, DMSO-D₆, ppm)

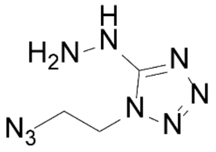

5

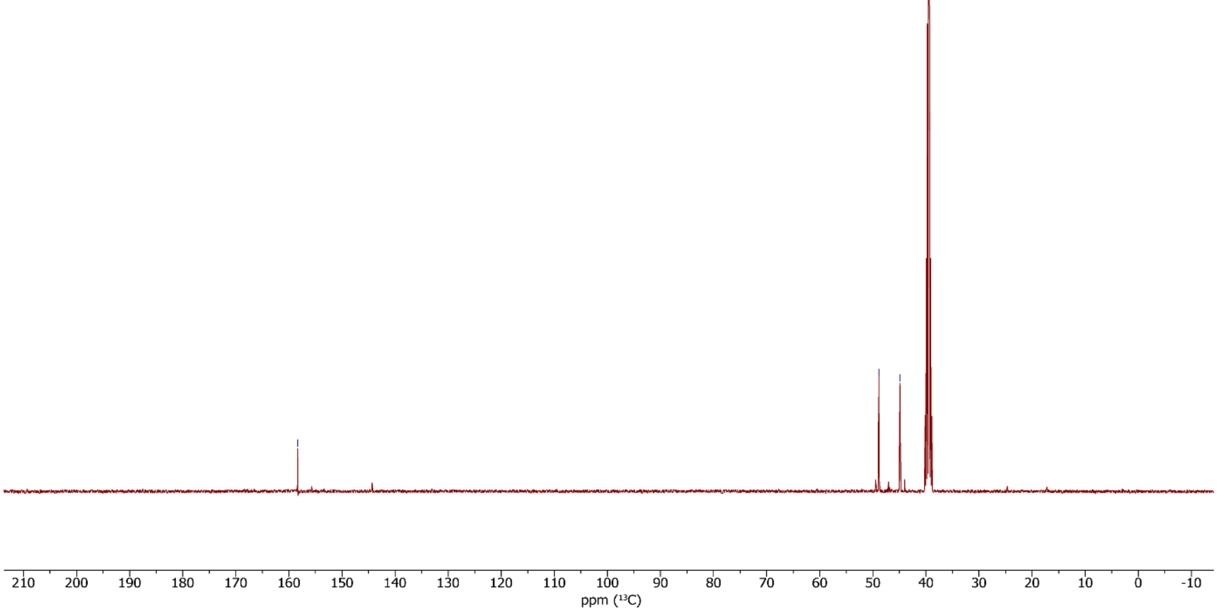

^{1}H NMR (400 MHz, D_2O, ppm)

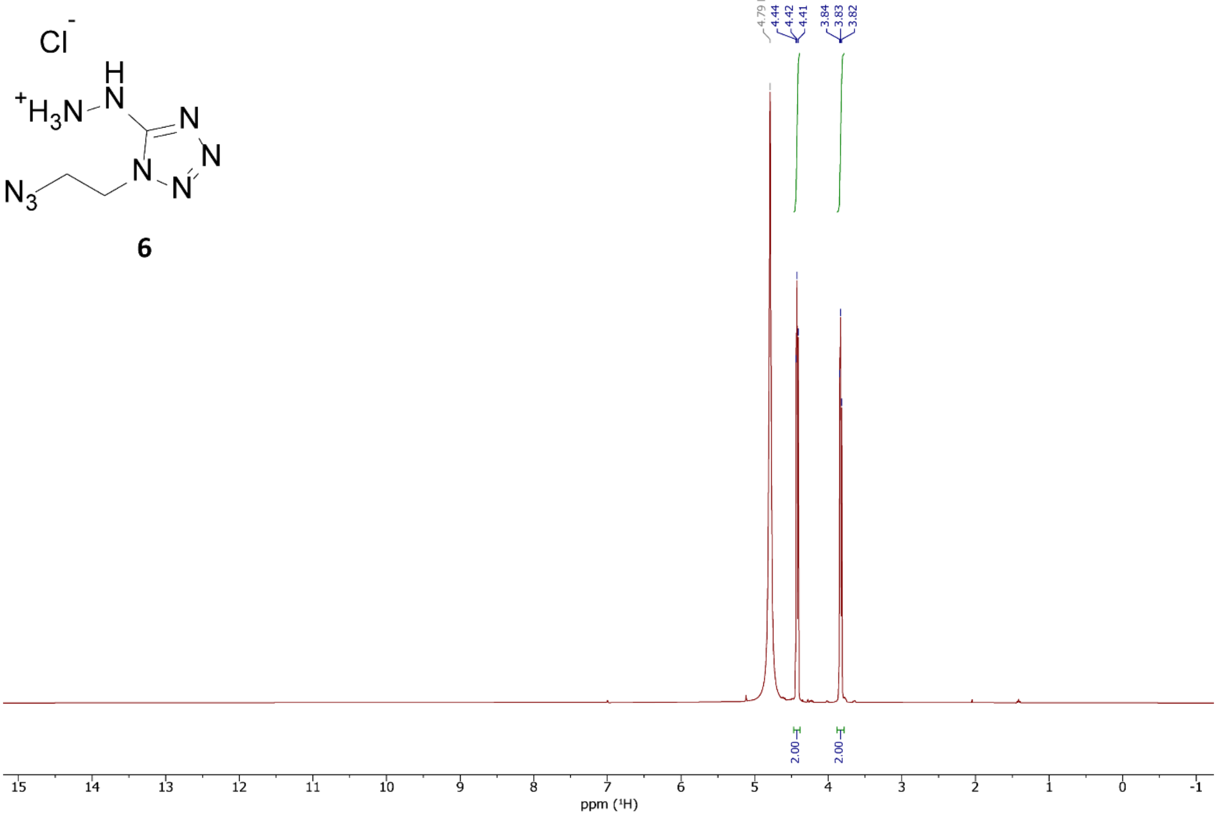

6

^{13}C NMR (101 MHz, D_2O, ppm)

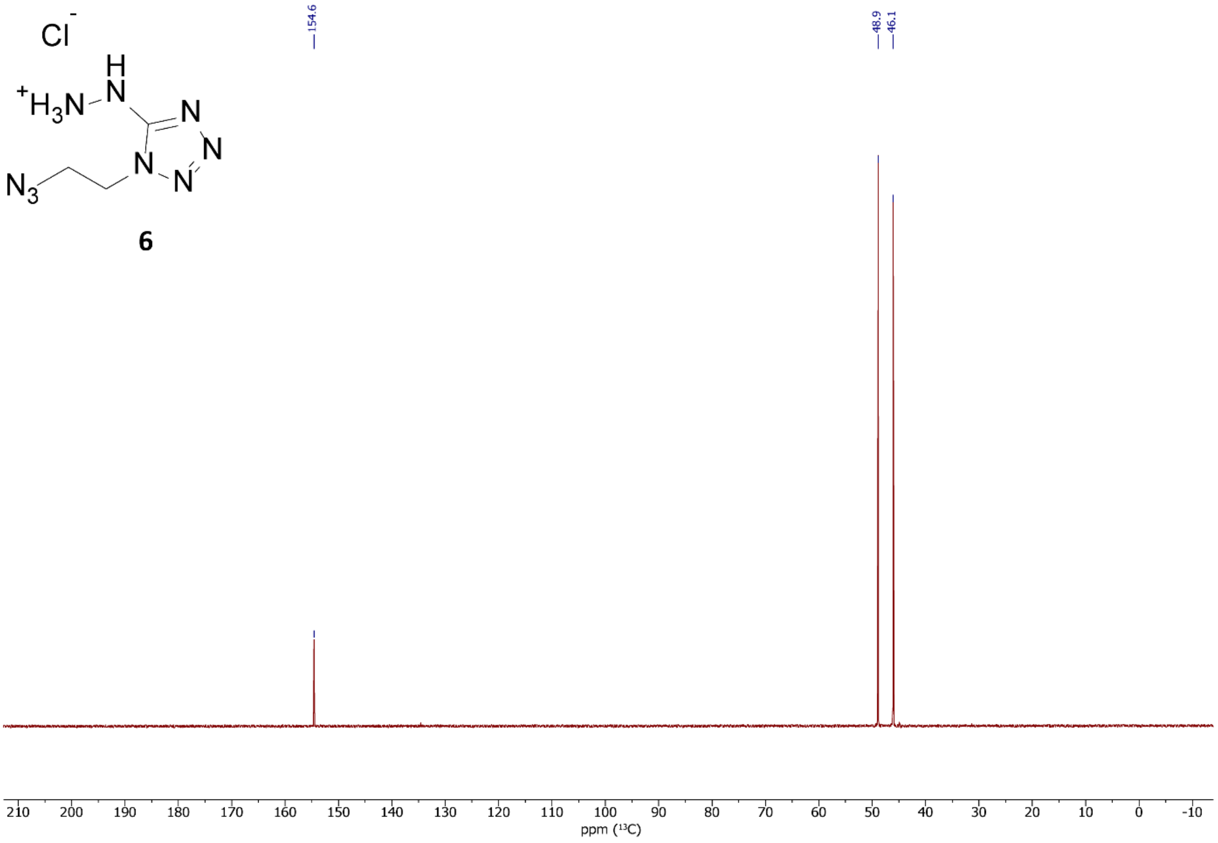

6

^{1}H NMR (400 MHz, DMSO-D₆, ppm)

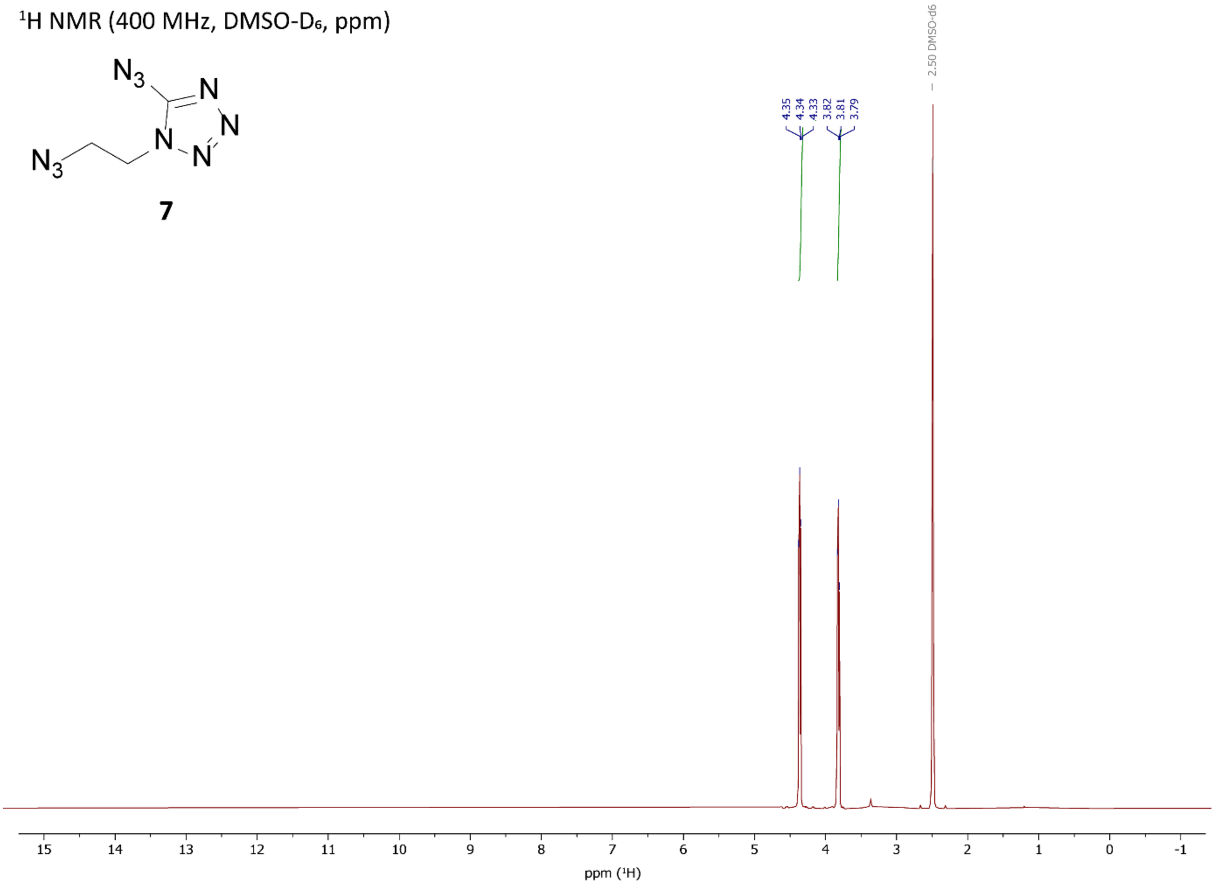

7

^{13}C NMR (101 MHz, DMSO-D₆, ppm)

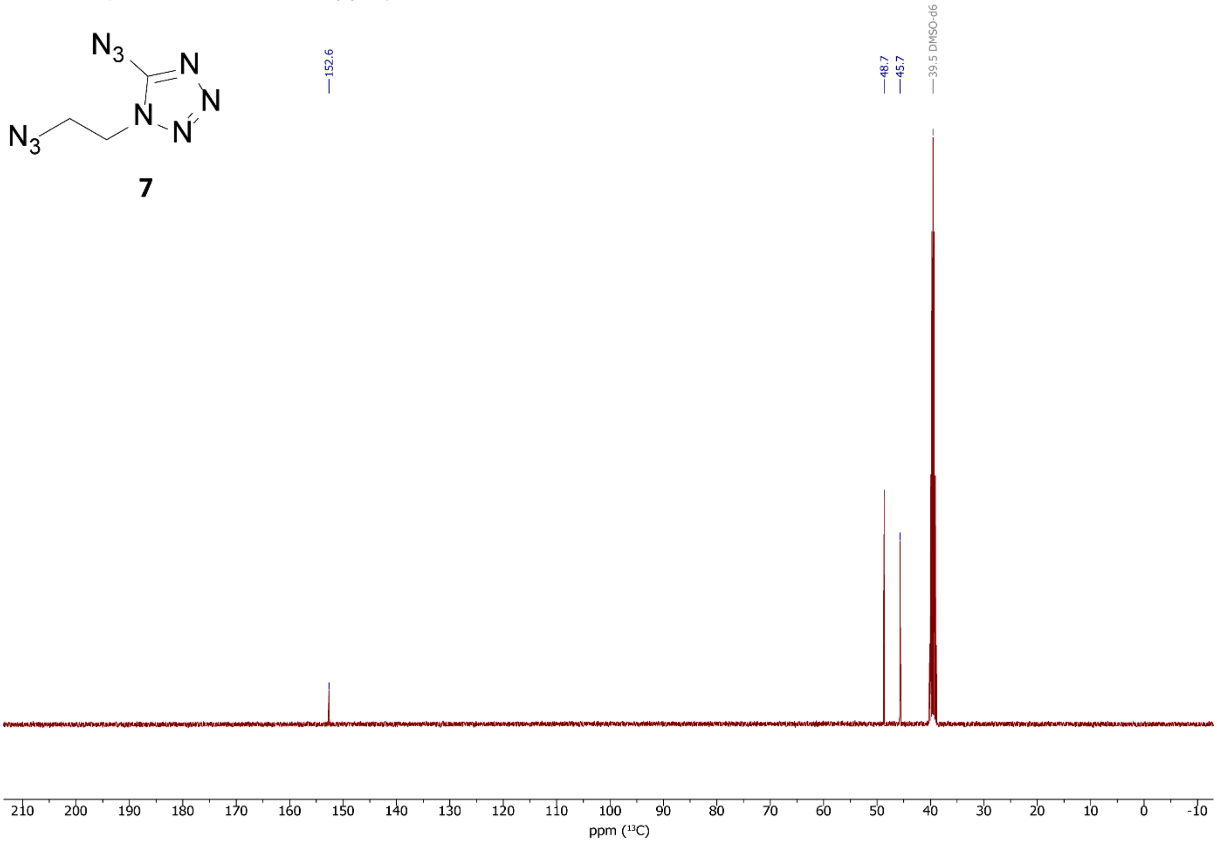

7

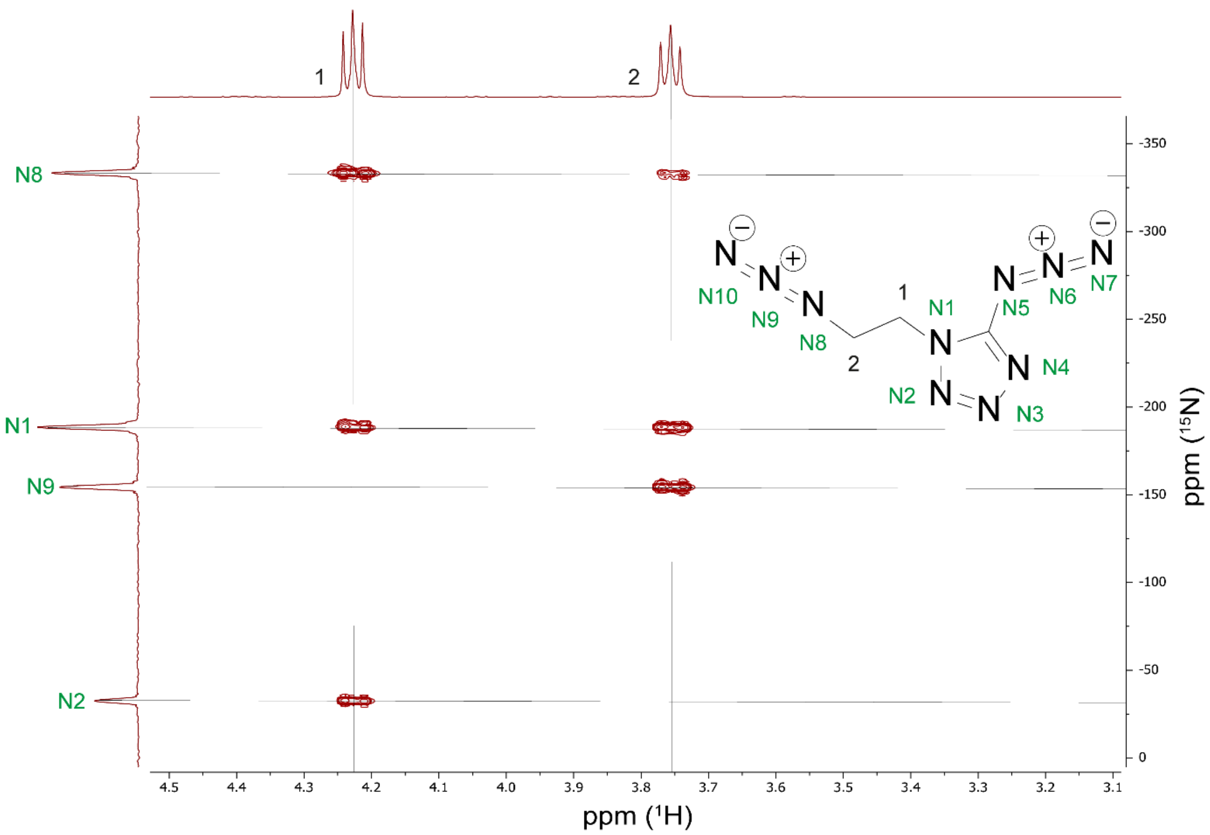

¹H NMR (400 MHz, DMSO-D₆, ppm)

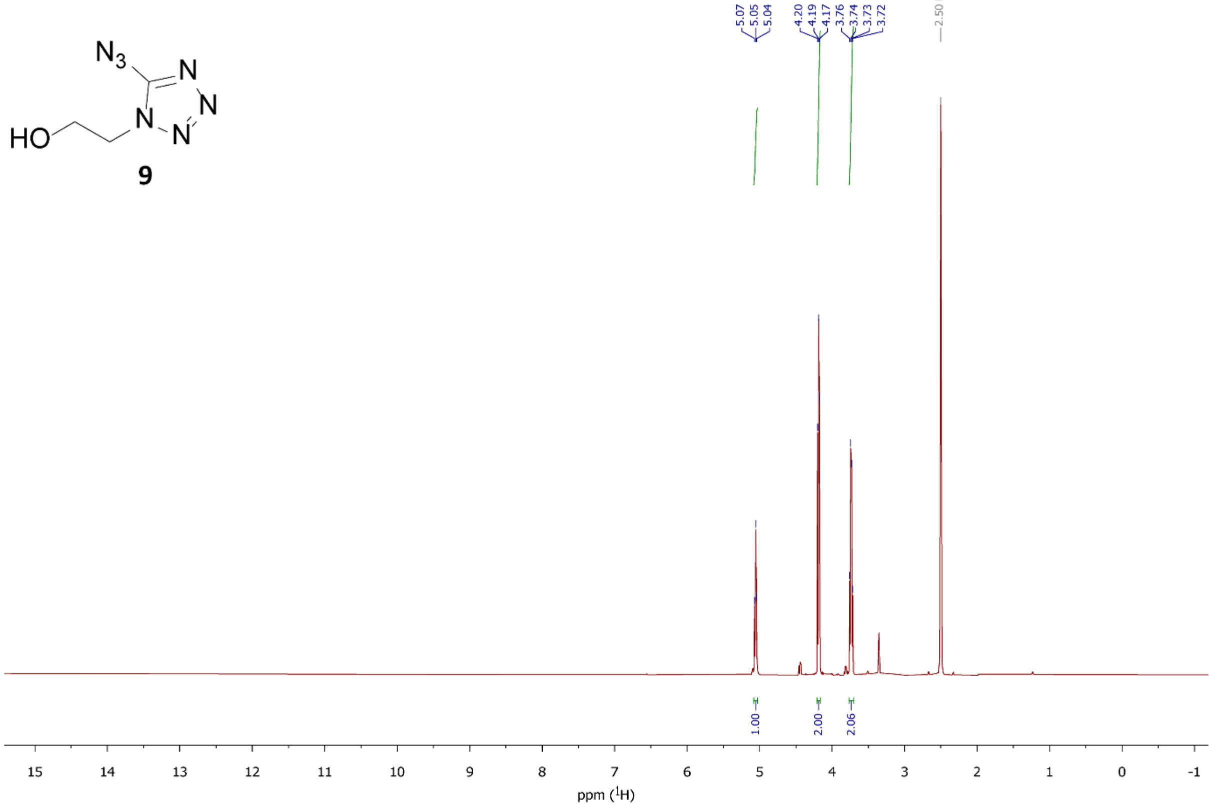

^{13}C NMR (101 MHz, DMSO-D₆, ppm)

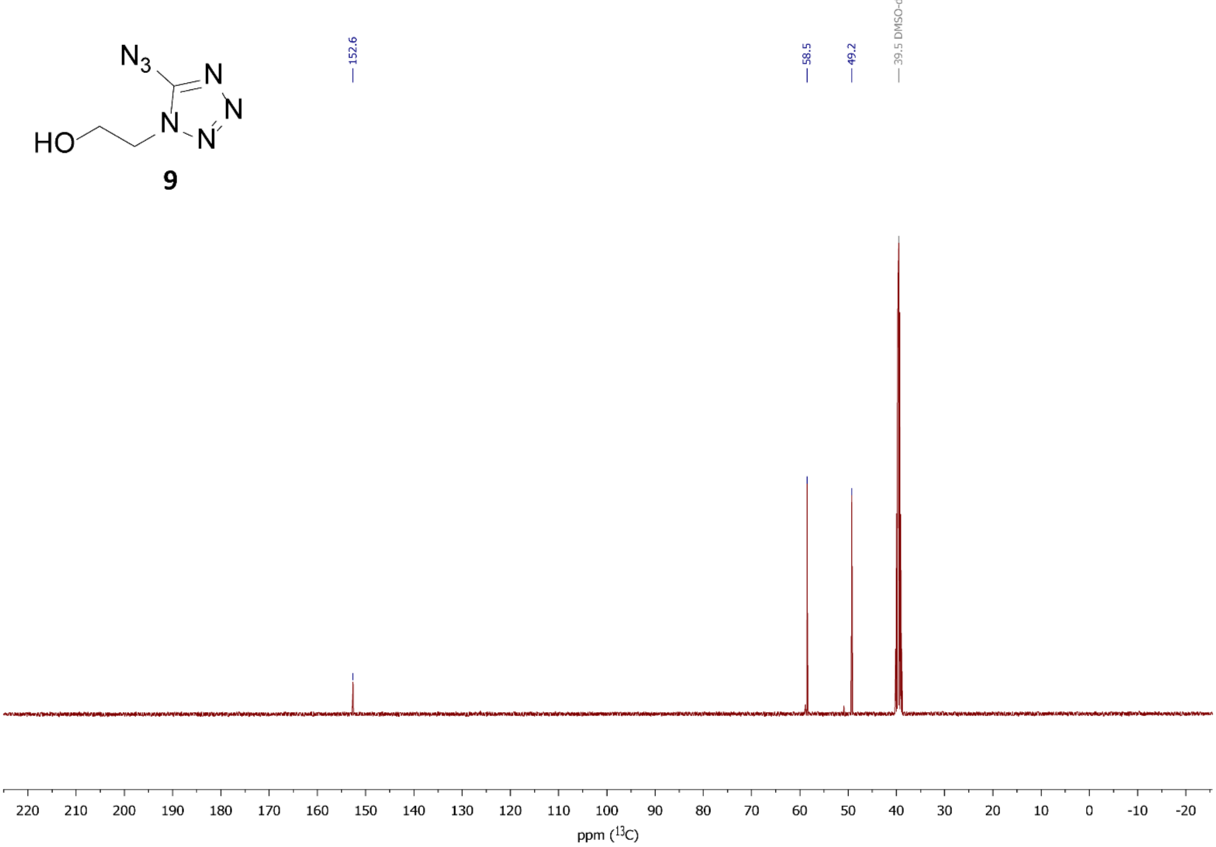

^{1}H NMR (400 MHz, Acetone-D₆, ppm)

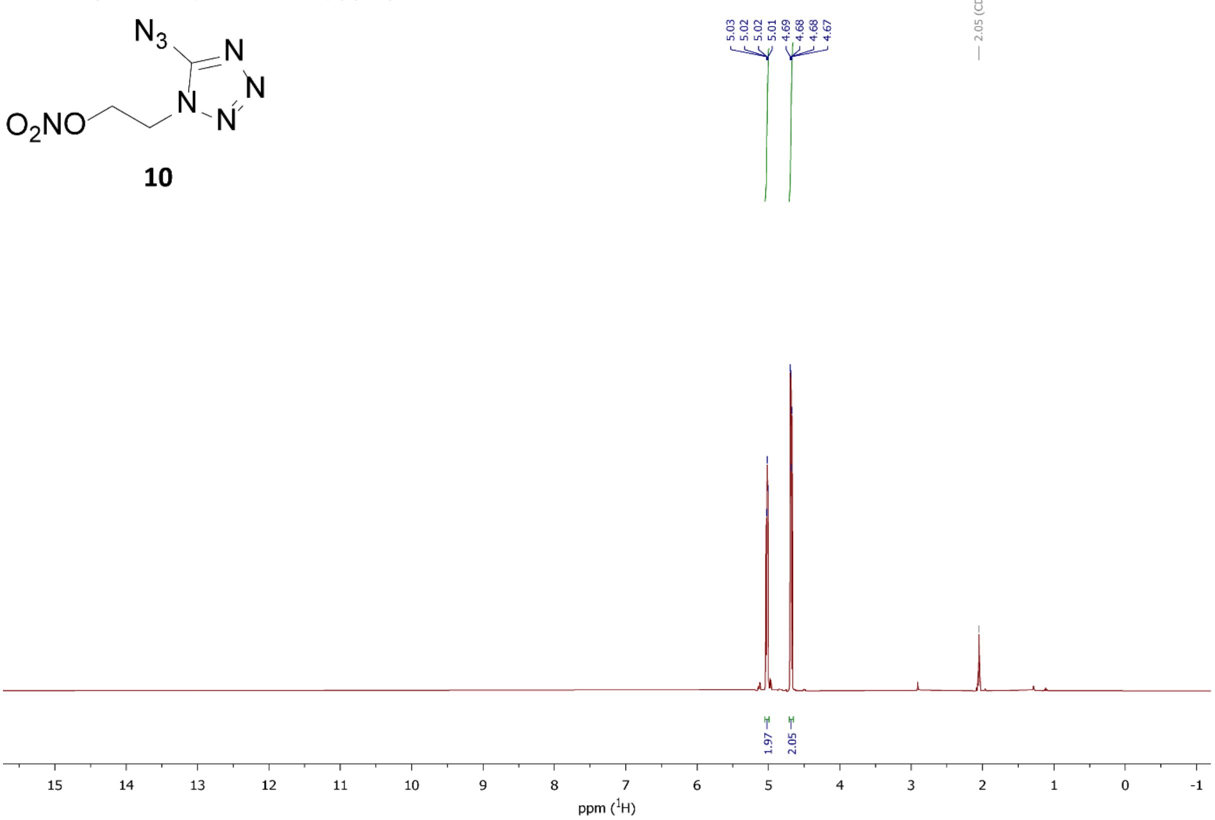

^{13}C NMR (101 MHz, Acetone-D₆, ppm)

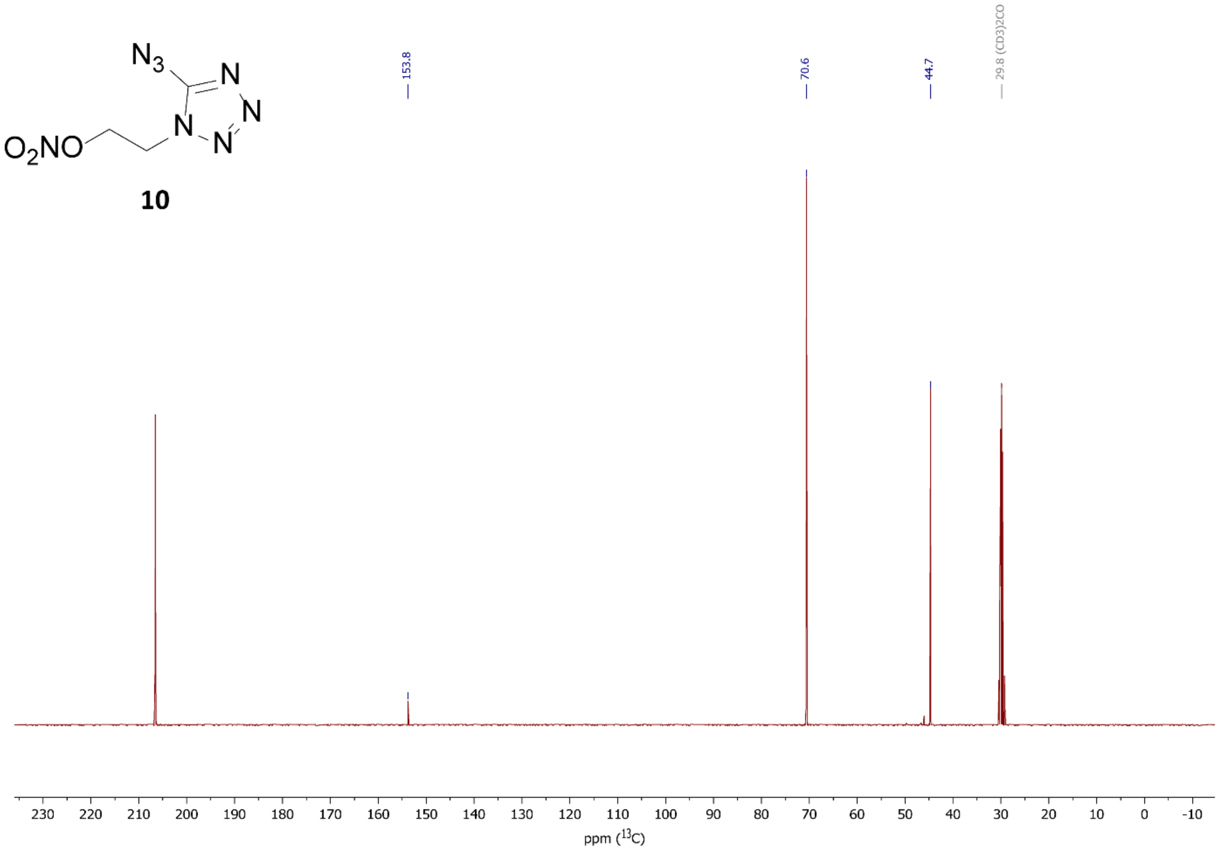

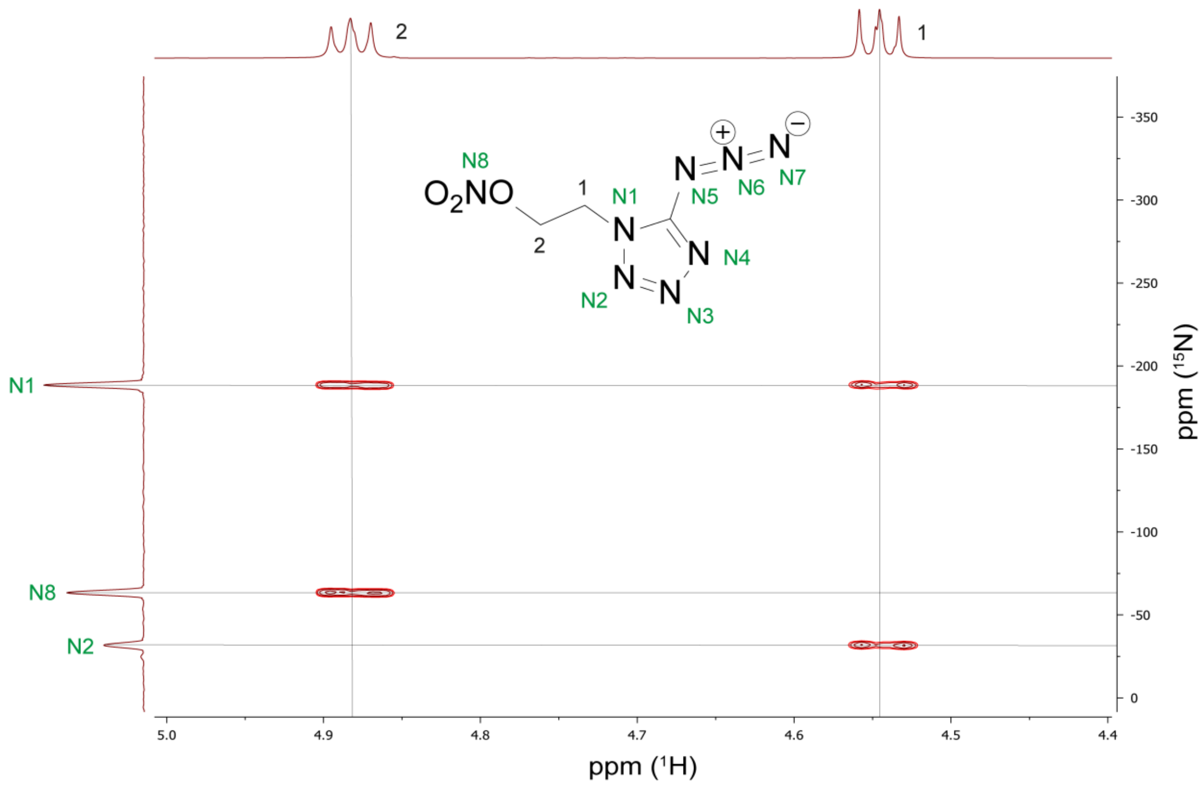

6. References

[S1] a) Reichel & Partner GmbH, http://www.reichelt-partner.de; b) Test methods according to the UN Recommendations on the Transport of Dangerous Goods, *Manual of Test and Criteria*, fourth revised edition, United Nations Publication, New York and Geneva, **2003**, ISBN 92–1-139087–7, Sales No. E.03.VIII.2; 13.4.2 Test 3 a (ii) BAM Fallhammer.

[S2] M. Sućeska, EXPLO5 V6.02 program, Brodarski Institute, Zagreb, Croatia, **2014**.

[S3] R. Yang, Z. Dong, Z. Ye, *ChemistrySelect* **2019**, *4*, 14208-14213.

[S4] D. E. Bayes (Glaxo Group Limited), EP0117368A1, **1982**.

[S5] *CrysAlisPro*, Oxford Diffraction Ltd. *version 171.33.41*, **2009**.

[S6] G. M. Sheldrick, *Acta Cryst.* **2015**, *A71*, 3–8.

[S7] O. V. Dolomanov, L. J Bourhis, R. J. Gildea, J. A. K. Howard, H. Puschmann, *J. Appl. Cryst.* **2009**, *42*, 339–341.

[S8] *SCALE3 ABSPACK – An Oxford Diffraction program* (1.0.4, gui: 1.0.3), Oxford Diffraction Ltd., **2005**.

[S9] *APEX3*. Bruker AXS Inc., Madison, Wisconsin, USA.

[S10] M. J. Frisch, G. W. Trucks, H. B. Schlegel, G. E. Scuseria, M. A. Robb, J. R. Cheeseman, G. Scalmani, V. Barone, B. Mennucci, G. A. Petersson, H. Nakatsuji, M. Caricato, X. Li, H.P. Hratchian, A. F. Izmaylov, J. Bloino, G. Zheng, J. L. Sonnenberg, M. Hada, M. Ehara, K. Toyota, R. Fukuda, J. Hasegawa, M. Ishida, T. Nakajima, Y. Honda, O. Kitao, H. Nakai, T. Vreven, J. A. Montgomery, Jr., J. E. Peralta, F. Ogliaro, M. Bearpark, J. J. Heyd, E. Brothers, K. N. Kudin, V. N. Staroverov, R. Kobayashi, J. Normand, K. Raghavachari, A. Rendell, J. C. Burant, S. S. Iyengar, J. Tomasi, M. Cossi, N. Rega, J. M. Millam, M. Klene, J. E. Knox, J. B. Cross, V. Bakken, C. Adamo, J. Jaramillo, R. Gomperts, R. E. Stratmann, O. Yazyev, A. J. Austin, R. Cammi, C. Pomelli, J. W. Ochterski, R. L. Martin, K. Morokuma, V. G. Zakrzewski, G. A. Voth, P. Salvador, J. J. Dannenberg, S. Dapprich, A. D. Daniels, O. Farkas, J.B. Foresman, J. V. Ortiz, J. Cioslowski, D. J. Fox, Gaussian 09 A.02, Gaussian, Inc., Wallingford, CT, USA, **2009**.

[S11] a) J. W. Ochterski, G. A. Petersson, and J. A. Montgomery Jr., *J. Chem. Phys.* **1996**, *104*, 2598–2619; b) J. A. Montgomery Jr., M. J. Frisch, J. W. Ochterski G. A. Petersson, *J. Chem. Phys.* **2000**, *112*, 6532–6542.

[S12] a) L. A. Curtiss, K. Raghavachari, P. C. Redfern, J. A. Pople, *J. Chem. Phys.* **1997**, *106*, 1063–1079; b) E. F. C. Byrd, B. M. Rice, *J. Phys. Chem*. A **2006**, *110*, 1005–1013; c) B. M. Rice, S. V. Pai, J. Hare, *Comb. Flame* **1999**, *118*, 445–458.

[S13] P. J. Lindstrom, W. G. Mallard (Editors), NIST Standard Reference Database Number 69, http://webbook.nist.gov/chemistry/ (accessed June **2020**).

[S14] M. S. Westwell, M. S. Searle, D. J. Wales, D. H. Williams, *J. Am. Chem. Soc*. **1995**, *117*, 5013–5015; b) F. Trouton, *Philos. Mag*. **1884**, *18*, 54–57.

[S15] a) H. D. B. Jenkins, H. K. Roobottom, J. Passmore, L. Glasser, *Inorg. Chem.* **1999,** *38,* 3609–3620; b) H. D. B. Jenkins, D. Tudela, L. Glasser, *Inorg. Chem.* **2002**, *41*, 2364–2367.
